# Beyond proximity: how food quality in dollar and grocery stores influences health

**DOI:** 10.1186/s12889-026-28145-y

**Published:** 2026-06-18

**Authors:** Ryan Bruellman, Jessica Finlay, Ilana J. Bennett, Chandra A. Reynolds

**Affiliations:** 1 Genetics, Genomics and Bioinformatics, University of California Riverside, 1140 Batchelor Hall, CA Riverside, USA; 2 Department of Geography, University of Colorado, Boulder, CO USA; 3 Institute of Behavioral Science, University of Colorado, Boulder, CO USA; 4 Institute for Behavioral Genetics, University of Colorado, Boulder, CO USA; 5 Department of Psychology, University of California Riverside, Riverside, CA USA; 6 Department of Psychology and Neuroscience, University of Colorado, Boulder, CO USA

**Keywords:** Cardiometabolic health, Built environment, Food access, Food deserts, Food swamps, Geospatial network analysis

## Abstract

**Background:**

Rising costs of groceries have prompted some United States (US) households to change their grocery shopping habits opting for dollar stores to lower household expenses. In previous work dollar stores have not been classified as a grocery store, limiting our understanding of how the dollar store associates with public health. Importantly, healthy food offerings such as fresh produce, whole grains, and lean proteins largely differ between traditional grocery stores and dollar stores. This public health study seeks to understand how the contrast in healthy food offerings associate with health outcomes across communities.

**Methods:**

We used 2016–2020 data where traditional grocery store and dollar store locations across the contiguous US were geocoded and assigned as standard quality (SQ) and low-quality (LQ) respectively. Access to SQ and LQ was gleaned using ArcGIS network analysis of these geocoded points with census tracts of metro areas represented by the 2019 release of the Centers for Disease Control (CDC) 500 Cities Project (2016–2017 estimates). ArcGIS generalized linear regression models analyzed links between health outcomes from the CDC 500 Cities Project and key variables: proximity to SQ stores, proximity to LQ stores, and an aggregate of socioeconomic factors indexed by the 2016 CDC Social Vulnerability Index (SVI).

**Results:**

Results across and within most regions of the US demonstrated significant negative associations between proximity to SQ stores and adverse health outcomes such as obesity, diabetes, and high blood pressure. Conversely, proximity to LQ stores (i.e., dollar stores) demonstrated significant positive associations with all health outcomes across the US, even after accounting for SVI.

**Conclusions:**

This study demonstrates the importance of dollar stores with food environment, an important component of food policy. Beyond recognizing dollar stores as grocery sources, these results emphasize the need to incorporate grocery quality into food environment research and policymaking.

**Supplementary Information:**

The online version contains supplementary material available at https://doi.org/10.1186/s12889-026-28145-y.

## Background

Food environment research and policy prioritize measures of grocery store access, typically defined by physical proximity, yet rarely account for the quality of foods offered in these outlets. The United States Department of Agriculture (USDA) designates “food deserts” as areas with high levels of poverty and low levels of access to traditional grocery stores [1]. Food deserts receive substantial government funding to improve grocery store access, however, these interventions often fail to improve the disparate health behaviors or outcomes present in these areas across the United States [2–5]. “Food swamps”, alternatively, reflect areas saturated with bodegas, convenience stores, and fast-food outlets where processed foods dominate and alternatives such as fresh produce and whole grains are sparse [6]. Compared to food deserts, food swamps show stronger associations with poor health outcomes such as obesity in many contexts [7, 8]; however, policy efforts to address components of food swamps have been unsuccessful [9]. Recent work has highlighted issues with classifying areas as food deserts or food swamps [10]; instead, studies note possible advantages to using a more multidimensional approach through broader incorporation of individual and household mobility, affordability, acceptability, time use, and activity space in considering grocery environments and access [7, 10–13]. Beyond discrete categorical labels and often-oversimplified food desert/food swamp approaches [1, 6], it is important to account for additional grocery sources beyond the market mainstays to better understand how varying grocery sources may help individuals, households, and communities meet their food needs. The dollar store is a grocery source often excluded from a grocery environment because it is categorized by the United States Census Bureau as a general merchandise store [7, 14]. Given the parallel low quality of grocery offerings between dollar stores to bodegas and convenience stores [15], the current study aimed to test whether dollar store access shows associations with poorer health outcomes, relative to traditional grocery stores.

Grocery store quality offers an important target for health policy given that many Americans now opt and need to shop at lower-cost, but also often lower-quality, grocery sources such as dollar stores [16]. For example, while traditional grocery stores food sales decreased by almost 7% from 2008 to 2020, dollar store food sales increased by over 3% over the same time period [15]. This trend is due in-part to the rise in inflation since the early 2000’s [17], increased cost of living and mismatched wages, and dollar stores marketing to lower-income households and communities [18, 19]. Traditional grocery stores offer a wide variety of food and a large selection of fresh fruits and vegetables, healthy grains and proteins—all of which are nutrient-dense and thus have positive associations with healthy diet choices [20]. Dollar stores are a retailer and grocery source in the United States. They contrast with other grocery and food sources due to factors such as their business model and scale, product offerings, and target customer base. The popularity of the dollar store has ballooned over previous decades due in large part to their ability to sell a wide variety of retail and grocery items for a low-cost relative to other big-box retailers, traditional grocery stores, and local private stores [16]. Grocery items sold at dollar stores are typically those that are highly processed and low-quality (e.g., minimal nutritional value) that fit within their business model of shelf-stable food products with a higher profit margin compared to fresh products at many traditional grocery sources [15, 19, 21]. Unlike big-box retailers and traditional grocery stores, dollar stores typically require a smaller store size yet attract many customers due to more product offerings compared to a convenience store or corner store [22]. Additionally, dollar stores have gained a specific footprint within the United States given their prevalence in low-income areas, offering a convenient and often more-affordable option for retail and grocery needs [22]. Dollar stores can influence the food environment and provide another option for grocery access, particularly for low-income areas where traditional grocery stores are lacking [16, 23]. Recent work found indirect evidence that while households increasingly rely on dollar stores for largely unhealthy options, some of these households may compensate by purchasing healthier foods from traditional grocery source outlets [15].

The shifting trends in grocery shopping are critical to consider in individual and community health, where increased reliance on dollar stores may portend increased disease prevalence given the relative lack of higher quality, nutrient-dense fresh foods. At the individual level, previous work shows that a poor-quality diet is linked with increased disease prevalence and progression, particularly in cardiometabolic conditions (i.e., obesity, diabetes, hypertension, dyslipidemia); these conditions are linked to premature aging [24–27]. On a community level, poor diet quality has been particularly prevalent in low-income groups who often experience disproportionally higher rates of cardiometabolic outcomes compared to higher income groups [28, 29]. We hypothesize that increased access to dollar stores will show associations with poorer health outcomes, similar to findings for food swamps [7, 8]. However, no work to date has directly compared dollar store to traditional grocery store effects on neighborhood health metrics.

This study examines whether access to standard-quality (SQ) traditional grocery stores versus low-quality (LQ) grocery stores (i.e., dollar stores) is associated with key health outcomes including diet-relevant cardiometabolic measures (e.g., obesity, diabetes, high blood pressure, high cholesterol) as well as less diet-relevant conditions (e.g., asthma, mental health). All dollar stores were designated as LQ grocery stores due to the lack of nutrient-dense grocery options such as whole grains, fresh produce, and lean proteins. We utilized geospatial network analysis to determine access to LQ and SQ grocery stores and then applied regression analysis to evaluate LQ and SQ access associations with different health outcomes, while considering socioeconomic factors. Study findings offer an important detail within one’s grocery environment by bringing in the quality of food options present at nearby grocery sources. This quality of a grocery source is an important nuance in considering public health interventions across grocery environments.

## Methods

### Grocery store access and quality

Grocery store access was defined as the total number of geocoded stores within a 10-minute drive for individuals within a given census tract (areas within counties that typically have 2,500 to 8,000 residents [30]), as in prior work [31, 32]. We obtained data on the location and type of traditional grocery store chains and dollar store chains between 2016 and 2020 across the contiguous United States (US) using POI Factory, an open-source website for geospatial datapoints [33]. In addition, we used OpenStreetMaps and Octoparse to gather grocery store brands not found on POI Factory [34, 35]. Checks were completed using spreadsheet tools in Microsoft Excel for reviewing latitude, longitude, and address data where duplicates or locations outside of the United States were removed. In total, we geocoded 30,920 traditional grocery store locations and 34,021 dollar store locations across the US. A full list of stores and the corresponding number of datapoints can be found in Supplemental Table 1.

For grocery store quality, we classified traditional grocery stores as standard-quality (SQ) and dollar stores were classified as low-quality (LQ). This was based on the typical lack of fresh produce, whole grains, and protein sources in dollar stores relative to traditional grocery stores [21, 22, 36–38].

### Health outcomes

We obtained disease/condition rates for obesity, diabetes, high blood pressure, high cholesterol, asthma, and poor mental health (mental distress for over two weeks, including stress, depression, and emotional problems) across US census tracts in the contiguous US from the 2019 release of the CDC’s 500 Cities Project which included rates from 2016 to 2017 [39]. To test disease rate pattern stability over time, we conducted additional correlation analysis of these 2016–2017 health outcomes across 2010 census tracts to determine consistency of health outcomes from release dates from 2013 to 2022. These data represented over 50 major urban/metropolitan areas utilizing cities within the 500 Cities Project within a common metro area within six different regions of the country (Table 1). A total of 19,607 census tracts are represented (representing 72% of all 500 Cities Project census tracts) across these metro areas accounting for over 76 million individuals (representing 74.6% of all 500 Cities Project tract population estimates).

**Table 1:** Regions and Metro Areas Analyzed

Region	City/Metro	Region	City/Metro
Midwest	Chicago	Pacific	Inland Empire
	Cincinnati		Los Angeles
	Cleveland		Orange County
	Columbus		Portland
	Des Moines		Sacramento
	Detroit		San Diego
	Indianapolis		San Francisco
	Kansas City		Seattle
	Milwaukee	South	Austin
	Minneapolis		Birmingham
	Omaha		Dallas
	St Louis		El Paso
Northeast	Baltimore		Houston
	Boston		Jackson
	Long Island		Little Rock
	Manhattan		Louisville
	New Jersey		Memphis
	Philadelphia		Nashville
	Pittsburgh		New Orleans
	Providence		Oklahoma City
	Staten Island		San Antonio
	Washington DC	South Atlantic	Atlanta
Mountain	Albuquerque		Charleston
	Boise		Charlotte
	Colorado Springs		Jacksonville
	Denver		Miami
	Las Vegas		Orlando
	Phoenix		Raleigh
	Salt Lake City		Tampa
	Tucson		Virginia Beach

### Socioeconomic variables

We included socioeconomic factors using the 2016 CDC’s overall Social Vulnerability Index (SVI) [40] to capture an aggregate of community-level factors that may influence food environment and food shopping behavior. SVI is a composite score that includes such variables as area-level income, vehicle access, and unemployment. The SVI overall index ranges from 0 to 1, with 1 indicating higher vulnerability (i.e., lower incomes, lower vehicle access, higher unemployment) in that census tract.

### Statistical analysis

We completed two network analyses using ArcGIS Pro 3.3.2 [41]. The first network analysis created network buffers to represent a 10-minute drive for individuals within a census tract, and a spatial join was completed to capture the total number of SQ data points within the network buffer. The second network analysis was completed in the same manner; however, it captured the total number of LQ data points within the network buffer. Next, we ran generalized linear regression models within ArcGIS for each of the six health outcomes with the network analyses for SQ grocery stores (traditional grocery stores), LQ grocery stores (dollar stores), and the overall SVI as main effects. Our analysis focused on grocery sources. Other food source datapoints such as fast-food establishments and convenience stores were not included in the analysis. These generalized linear regression models for each health outcome were first run across the contiguous United States, then by each separate US Census region (e.g., Pacific, Mountain, South). All explanatory variables (number of SQ grocery stores within a 10-minute drive, number of LQ grocery stores within a 10-minute drive, and the CDC Overall SVI for the given tract) were mean centered and standardized for the main analysis. Additional analysis using original variable scaling and mean-centered can be found in Supplemental Table 2. For a visual analysis across the metro areas (Table 1), we utilized the ArcGIS kernel density analysis tool. For each metro area, a separate kernel density analysis was completed for LQ grocery stores utilizing the dollar store locations as the input points and one for SQ grocery stores utilizing traditional grocery store locations as input points.

## Results

### Descriptive statistics and correlations

Descriptive statistics for each predictor and outcome variable are provided in Table 2, separately for the contiguous US and each region. Across the US, the mean number of driving accessible LQ grocery stores was lower than SQ grocery stores, with LQ grocery stores accounting for over 33% of the accessible grocery sources in the typical US census tract (Table 2). Regional data showed that the average number of accessible LQ grocery stores was the highest in the Midwest, South, and South Atlantic Regions (> 40%) and the lowest in the Pacific Region (< 25%). The highest average rates of poor health outcomes were seen for obesity, high blood pressure, and high cholesterol with the Midwest, South, and South Atlantic Regions having the highest average rates and the Pacific Region having the lowest rates.

**Table 2:** Descriptive Statistics for Predictors and Outcome Variables

		USA	Midwest	Mountain	Northeast	Pacific	South	South Atlantic
LQ	Mean	15.68	22.91	14.16	10.78	7.87	25.68	18.60
	SD	12.19	12.90	8.83	8.81	5.61	13.84	10.29
	IQR	6–22	14–30	7–19	3–16	4–11	15–35	11–25
SQ	Mean	25.69	21.88	25.51	25.78	29.35	24.50	24.45
	SD	13.04	9.66	11.39	14.57	14.00	14.07	9.56
	IQR	16–33	15–27	17–33	16–32	19–37	14–33	18–31
SVI	Mean	0.57	0.59	0.48	0.63	0.57	0.56	0.52
	SD	0.30	0.31	0.31	0.27	0.29	0.32	0.31
	IQR	0.30–0.84	0.31–0.86	0.20–0.76	0.42–0.87	0.32–0.84	0.26–0.85	0.23–0.81
Obesity	Mean	29.45	35.25	27.04	27.81	24.43	34.10	31.08
	SD	8.18	8.54	5.23	8.25	5.56	7.19	6.92
	IQR	23.4–34.6	28.0–42.8	23.8–29.8	21.3–33.4	20.1–28.3	28.6–39.4	25.6–35.7
Diabetes	Mean	10.71	12.12	8.54	11.18	9.40	12.08	11.01
	SD	4.22	5.30	2.81	4.00	2.70	4.75	4.29
	IQR	7.8–12.9	8.2–15.9	6.6–10.0	8.6–13.6	7.5–11.1	8.5–14.9	7.9–13.4
High BP	Mean	30.07	33.67	26.42	29.94	26.25	33.54	32.58
	SD	8.20	10.17	5.72	7.85	4.72	8.57	8.01
	IQR	24.8–34.1	26.2–41.5	22.8–29.4	25–34.9	23.6–28.6	28.2–37.8	27.3–37.1
High Chol	Mean	31.11	31.21	30.15	30.78	30.13	32.98	32.07
	SD	4.62	4.94	4.67	4.20	3.94	4.73	5.05
	IQR	28.6–34.0	28.5–34.6	27.7–32.7	29–33.4	28.1–32.3	30.2–36.2	29.4–35.1
Asthma	Mean	9.63	10.45	9.96	10.24	8.92	9.25	9.23
	SD	1.74	2.28	0.87	1.79	1.12	1.75	1.63
	IQR	8.4–10.6	8.5–12.1	9.4–10.5	8.8–11.6	8.2–9.6	7.9–10.3	8.0–10.2
M Health	Mean	13.61	14.70	13.14	13.84	12.50	14.28	13.64
	SD	3.50	4.05	3.01	3.39	3.02	3.64	3.30
	IQR	10.9–16.0	11.5–17.7	10.9–15.0	11.4–16.1	10.1–14.5	11.4–16.9	11.2–15.8

Mean, standard deviation, and interquartile ranges of explanatory variables of grocery store access and socioeconomic vulnerability index as well as health-related outcomes. Values are represented across the contiguous United States and by region. LQ = Low-quality grocery stores, SQ = Standard-quality traditional grocery stores, SVI = Overall Social Vulnerability Index, BP = Blood Pressure, Chol = Cholesterol, M Health = Mental Health, SD = Standard deviation, IQR = Interquartile range

Correlations across health outcomes over years are noted in Supplemental Table 3 where the 2016–2017 estimates used in statistical analysis had strong correlations with earlier years such as 2013–2014 (correlation range = 0.92–0.97), later years such as 2019–2020 (correlation range = 0.89–0.97), and recent years such as 2021–2022 (correlation range = 0.81–0.93).

**Table 3:** Regression Results of Health Outcomes Across the USA and Regions

Regression Results Standardized Variables		Obesity			Diabetes			High BP		
		Coef	SE	p-value	Coef	SE	p-value	Coef	SE	p-value
USA	Intercept	29.45	0.03	***	10.71	0.02	***	30.07	0.05	***
	LQ Grocery Stores	4.16	0.04	***	1.32	0.02	***	3.39	0.05	***
	SQ Grocery Stores	-2.19	0.03	***	-0.73	0.02	***	-1.92	0.05	***
	SVI Overall	3.57	0.04	***	2.45	0.02	***	2.71	0.05	***
Pacific	Intercept	24.43	0.05	***	9.40	0.03	***	26.25	0.06	***
	LQ Grocery Stores	1.86	0.08	***	0.64	0.04	***	0.98	0.09	***
	SQ Grocery Stores	-1.95	0.07	***	-0.55	0.03	***	-1.16	0.08	***
	SVI Overall	3.09	0.06	***	1.72	0.03	***	1.32	0.07	***
Midwest	Intercept	35.25	0.08	***	12.12	0.06	***	33.67	0.12	***
	LQ Grocery Stores	2.47	0.09	***	1.56	0.07	***	3.89	0.13	***
	SQ Grocery Stores	-1.29	0.09	***	-0.68	0.06	***	-1.83	0.12	***
	SVI Overall	5.50	0.09	***	3.09	0.07	***	4.59	0.14	***
Northeast	Intercept	27.81	0.08	***	11.22	0.04	***	29.99	0.10	***
	LQ Grocery Stores	3.85	0.09	***	0.75	0.04	***	2.45	0.10	***
	SQ Grocery Stores	-2.15	0.08	***	-0.55	0.04	***	-1.71	0.10	***
	SVI Overall	4.33	0.09	***	2.85	0.04	***	4.02	0.10	***
Mountain	Intercept	27.04	0.07	***	8.54	0.05	***	26.42	0.12	***
	LQ Grocery Stores	1.54	0.10	***	0.86	0.06	***	0.76	0.16	***
	SQ Grocery Stores	-1.17	0.08	***	-0.32	0.05	***	0.26	0.13	*
	SVI Overall	3.03	0.09	***	1.25	0.06	***	1.17	0.15	***
South	Intercept	34.10	0.07	***	12.08	0.05	***	33.54	0.12	***
	LQ Grocery Stores	1.26	0.08	***	0.95	0.06	***	1.74	0.14	***
	SQ Grocery Stores	-1.43	0.07	***	-1.06	0.05	***	-2.54	0.12	***
	SVI Overall	4.97	0.08	***	2.89	0.06	***	3.27	0.14	***
South Atlantic	Intercept	30.94	0.09	***	10.94	0.06	***	32.46	0.13	***
	LQ Grocery Stores	2.33	0.11	***	0.75	0.08	***	1.92	0.17	***
	SQ Grocery Stores	-1.74	0.10	***	-0.77	0.07	***	-1.60	0.14	***
	SVI Overall	3.89	0.11	***	2.71	0.07	***	3.82	0.16	***

Regression Results Standardized Variables		High Chol			Asthma			M Health		
		Coef	SE	p-value	Coef	SE	p-value	Coef	SE	p-value
USA	Intercept	31.11	0.03	***	9.63	0.01	***	13.61	0.01	***
	LQ Grocery Stores	1.07	0.03	***	0.47	0.01	***	0.94	0.02	***
	SQ Grocery Stores	-0.46	0.03	***	-0.40	0.01	***	-0.58	0.01	***
	SVI Overall	0.74	0.03	***	0.77	0.01	***	2.37	0.02	***
Pacific	Intercept	30.13	0.05	***	8.92	0.01	***	12.50	0.03	***
	LQ Grocery Stores	0.46	0.08	***	0.20	0.02	***	0.45	0.04	***
	SQ Grocery Stores	-0.14	0.07	*	-0.36	0.02	***	-0.58	0.03	***
	SVI Overall	0.41	0.06	***	0.51	0.01	***	2.22	0.03	***
Midwest	Intercept	31.21	0.07	***	10.45	0.03	***	14.70	0.04	***
	LQ Grocery Stores	1.81	0.08	***	1.00	0.03	***	1.09	0.04	***
	SQ Grocery Stores	-1.12	0.08	***	-0.44	0.03	***	-0.25	0.04	***
	SVI Overall	0.81	0.08	***	1.03	0.03	***	2.70	0.05	***
Northeast	Intercept	30.80	0.06	***	10.25	0.02	***	13.88	0.03	***
	LQ Grocery Stores	-0.12	0.07	0.080	0.72	0.02	***	1.02	0.04	***
	SQ Grocery Stores	-0.50	0.06	***	-0.36	0.02	***	-0.57	0.03	***
	SVI Overall	1.61	0.07	***	0.82	0.02	***	2.27	0.04	***
Mountain	Intercept	30.15	0.10	***	9.96	0.01	***	13.14	0.03	***
	LQ Grocery Stores	0.45	0.14	***	0.18	0.02	***	0.59	0.05	***
	SQ Grocery Stores	0.24	0.11	*	-0.03	0.01	0.071	-0.55	0.04	***
	SVI Overall	0.05	0.13	0.678	0.52	0.02	***	2.18	0.04	***
South	Intercept	32.98	0.08	***	9.25	0.02	***	14.28	0.04	***
	LQ Grocery Stores	0.86	0.09	***	0.24	0.03	***	0.28	0.04	***
	SQ Grocery Stores	-0.66	0.08	***	-0.57	0.03	***	-0.63	0.04	***
	SVI Overall	0.95	0.09	***	0.70	0.03	***	2.72	0.04	***
South Atlantic	Intercept	32.05	0.11	***	9.20	0.03	***	13.58	0.04	***
	LQ Grocery Stores	0.01	0.13	0.960	0.63	0.03	***	0.74	0.05	***
	SQ Grocery Stores	-0.30	0.11	**	-0.38	0.03	***	-0.39	0.04	***
	SVI Overall	1.13	0.13	***	0.61	0.03	***	2.34	0.05	***

Regression results where all main effect variables are standardized and centered about the mean. Unstandardized regression results can be found on Supplemental Table 2. Notes: BP = blood pressure; Chol = cholesterol; M Health = mental health; Coef = coefficient, SE = standard error; LQ = low-quality; SQ = standard-quality; SVI = Social Vulnerability Index; * = *p*-value < 0.05, ** = *p*-value < 0.01, *** = *p*-value < 0.001

### Visual trends across cities

**Fig. 1:**
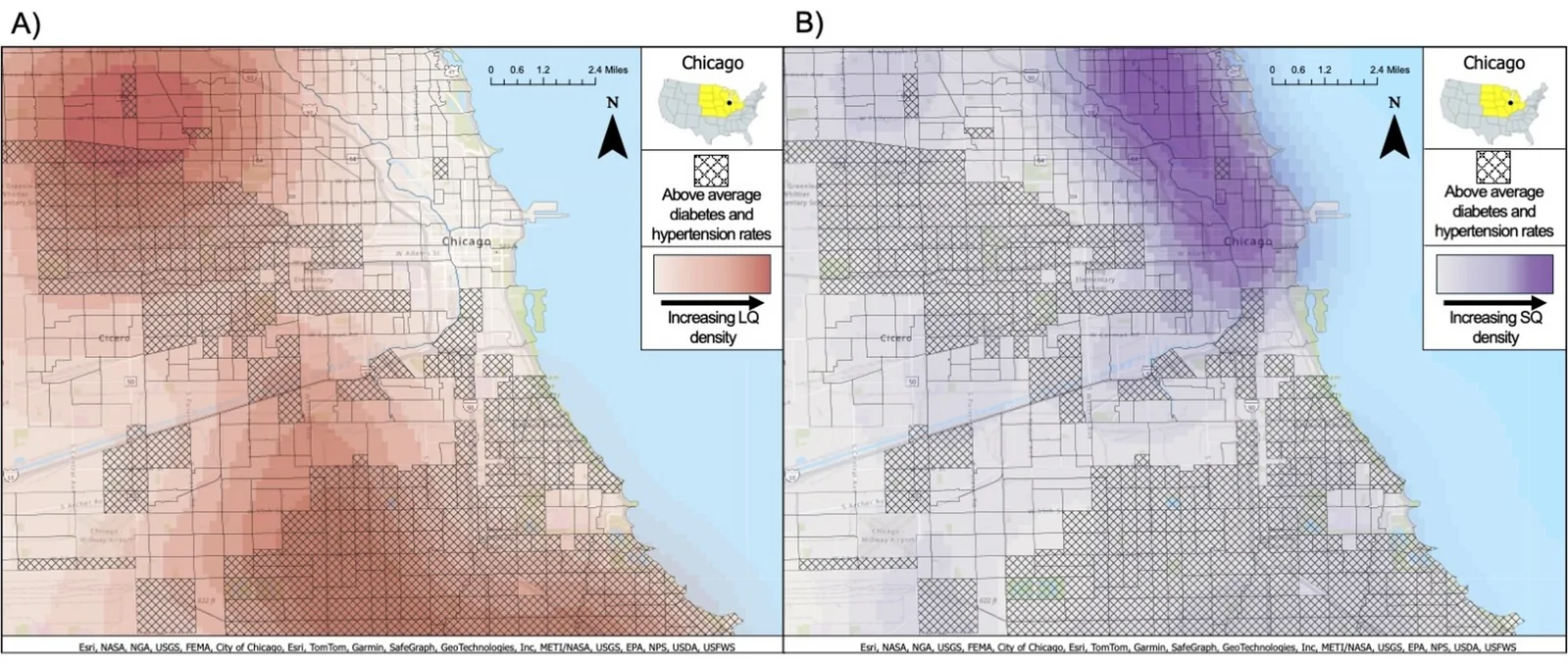
Side by side kernel analysis overlay with hatched tracts for the city of Chicago, Illinois. Hatched tracts represent above US average diabetes and hypertension rates. **A** Red kernel shaded areas are LQ (dollar store) dense areas. **B** Purple kernel shaded areas are SQ (traditional grocery store) dense areas. LQ = Low Quality, SQ = Standard Quality

**Fig. 2:**
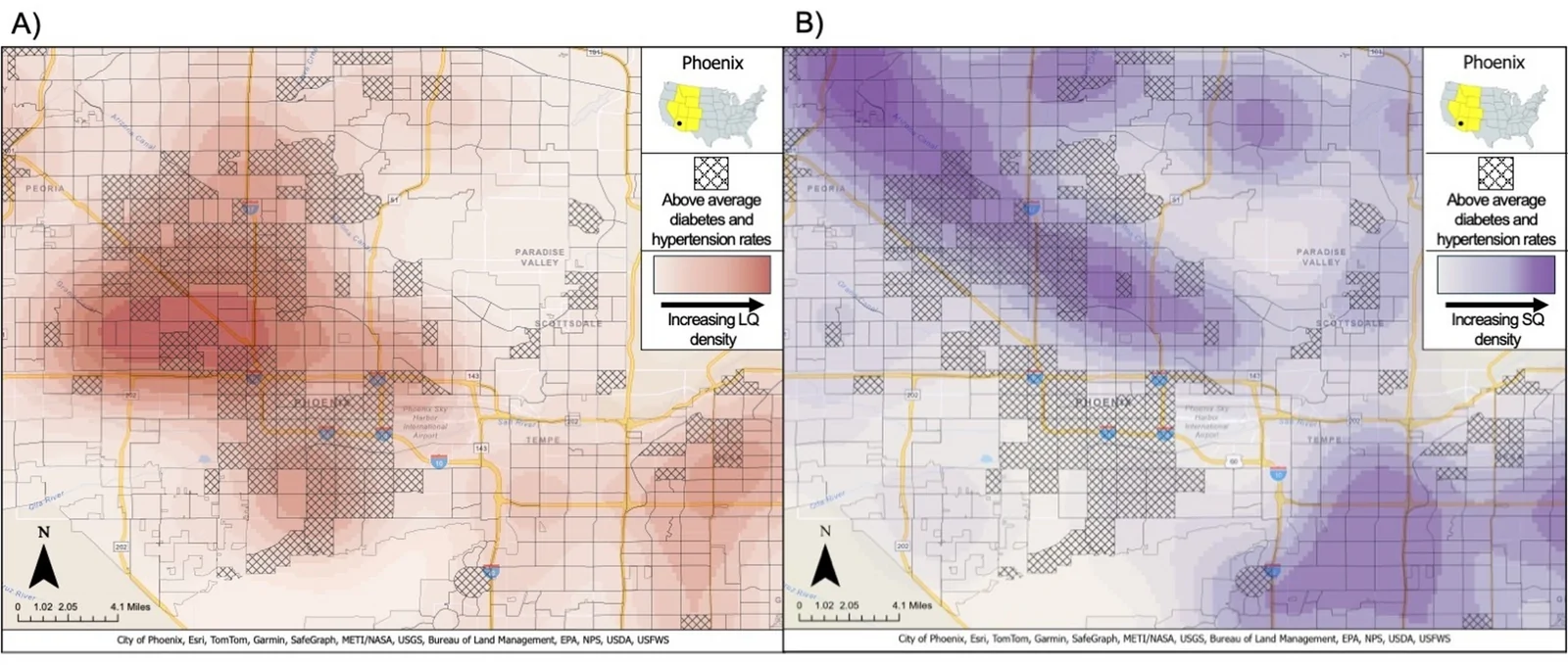
Side by side kernel analysis overlay with hatched tracts for the city of Phoenix, Arizona. Hatched tracts represent above US average diabetes and hypertension rates. **A** Red kernel shaded areas are LQ (dollar store) dense areas. **B** Purple kernel shaded areas are SQ (traditional grocery store) dense areas. LQ = Low Quality, SQ = Standard Quality

**Fig. 3:**
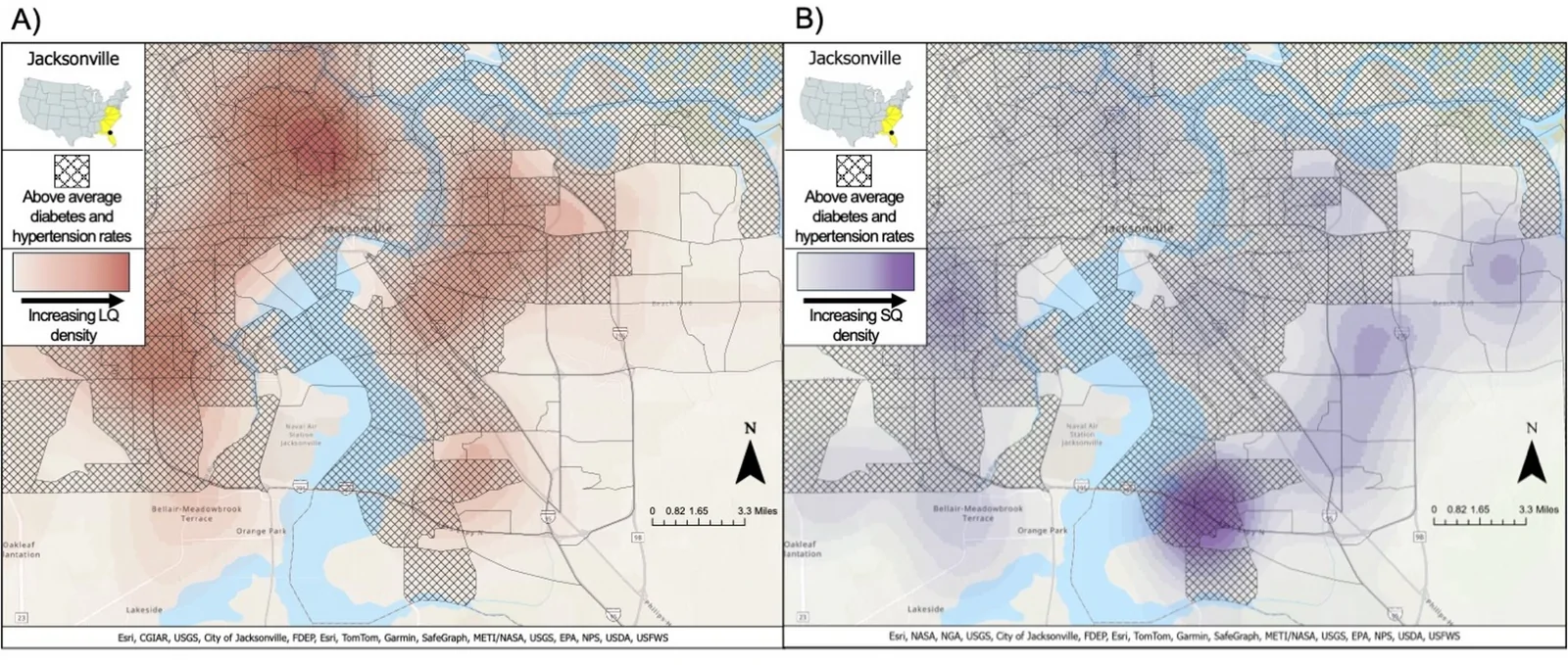
Side by side kernel analysis overlay with hatched tracts for the city of Jacksonville, Florida. Hatched tracts represent above US average diabetes and hypertension rates. **A** Red kernel shaded areas are LQ (dollar store) dense areas. **B** Purple kernel shaded areas are SQ (traditional grocery store) dense areas. LQ = Low Quality, SQ = Standard Quality

While each city has distinct characteristics and represents a different socio-geographic region across the country, there are consistent trends across many US metropolitan areas. For example, LQ grocery stores across the Midwest Region city of Chicago show clustering of above-average rates of poor cardiovascular and metabolic health (indicated by above average diabetes and hypertension) rates over the western and southern areas of the city (Fig. 1). Figure 1B also shows larger SQ grocery store clusters across the downtown and northern areas of Chicago where diabetes and hypertension rates are generally well below average. While these SQ grocery store clusters are present in areas across the west and south side of Chicago (Fig. 1B), they are overshadowed by dense areas of LQ grocery stores (Fig. 1A). Figure 2 illustrates similar trends across the Mountain Region city of Phoenix where higher rates of diabetes and hypertension are present across areas such as Central City and west of downtown, where dense areas of LQ grocery stores are prominent (Fig. 2A) along with some dense areas of SQ grocery stores (Fig. 2B). Figure 3 shows trends across the South Atlantic Region city of Jacksonville where dense areas of LQ coincide with hatched areas across much of the metro area (Fig. 3A). Areas with dense SQ areas are mixed in Jacksonville with health outcome rates (Fig. 3B). Other metro areas across all regions can be found in the Supplemental Figures.

## Linear regression analyses

### National models

**Fig. 4:**
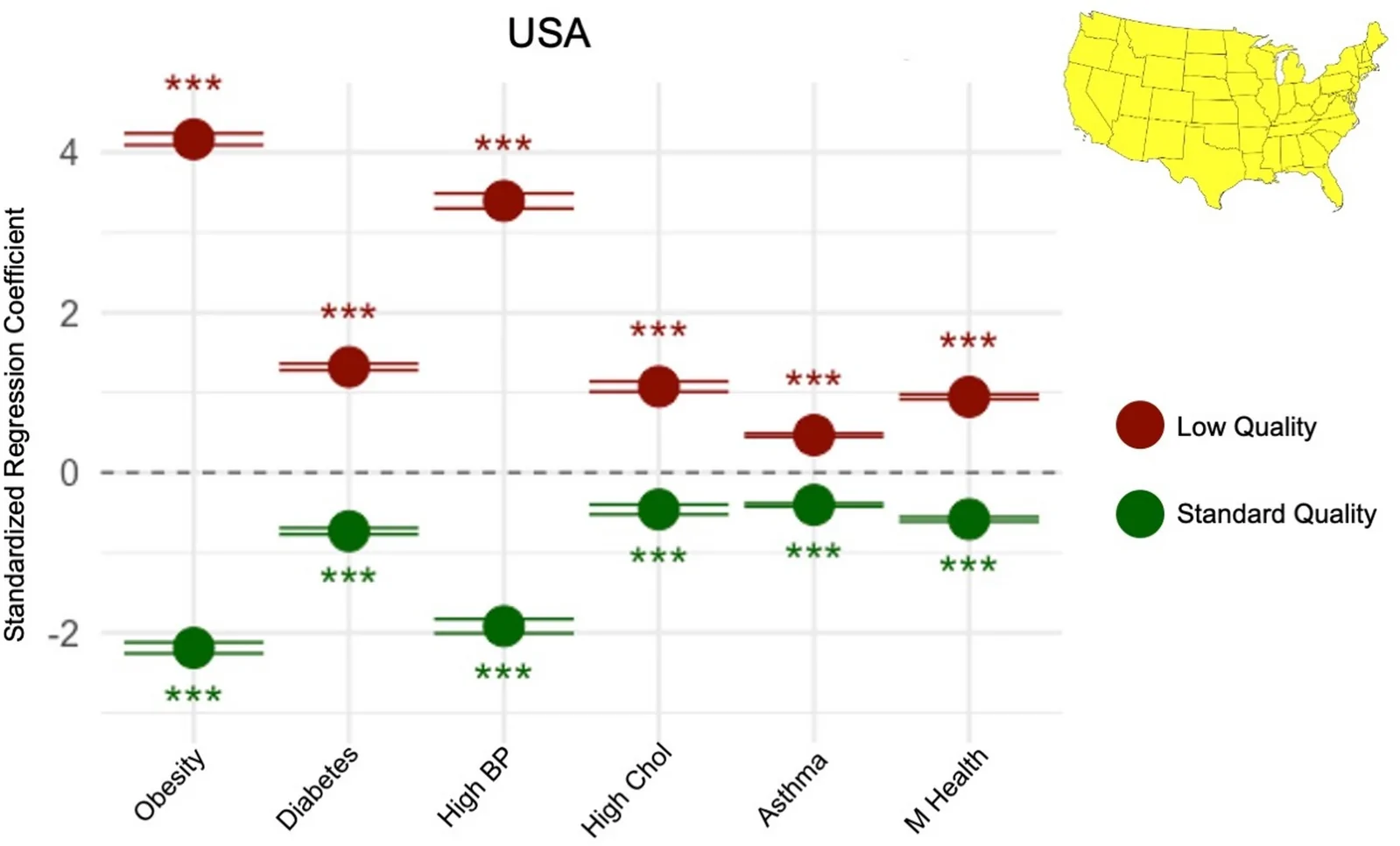
Visualization of regression results across contiguous United States and regions. Results are from all explanatory variables being standardized. High BP = High blood pressure; High Chol = High cholesterol, M Health = Mental Health. * = p-value < 0.05, ** = *p*-value < 0.01, *** = *p*-value < 0.001, ns = *p*-value ≥ 0.05

Across the US, all explanatory variables were significant (Fig. 4; Table 3, Supplemental Fig. 1), with greater LQ grocery store access and higher SVI associated with higher rates of obesity, diabetes, high blood pressure, high cholesterol, asthma, and poor mental health conditions. Greater SQ grocery store access was associated with lower rates of these health outcomes. For example, obesity rates showed a significant positive association with LQ grocery store access across the US (ß_LQ Grocery Stores_ = 4.16, *p* < 0.001); whereas SQ grocery access had a smaller, yet significant negative association with obesity rates (ß_SQ Grocery Stores_ = -2.19, *p* < 0.001). Across health outcomes, cardiometabolic health rates of obesity, diabetes, and high blood pressure showed the highest associations with LQ and SQ access. Other non-cardiometabolic health outcomes (i.e., asthma and poor mental health) still showed significance with LQ and SQ access, however, the associations were weaker (High BP ß_LQ Grocery Stores_ = 3.39 compared to Asthma ß_LQ Grocery Stores_ = 0.47, and High BP ß_SQ Grocery Stores_ = -1.92 compared to Asthma ß_SQ Grocery Stores_ = -0.40). To facilitate interpretability and policy relevance, model results with unstandardized variables are reported in Supplemental Table 2.

### Regional models

Regional trends were generally consistent with the overall US results except for high blood pressure in the Mountain Region (ß_SQ Grocery Stores_ = 0.26, *p* < 0.05); high cholesterol in the Northeast Region (ß_LQ Grocery Stores_ = − 0.12, *p* = 0.080), Mountain Region (ß_SQ Grocery Stores_ = 0.24, *p* < 0.05), and South Atlantic Region (ß_LQ Grocery Stores_ = 0.01, *p* = 0.960); and asthma in the Mountain Region (ß_SQ Grocery Stores_ = − 0.03, *p* = 0.071) (Table 3, Supplemental Fig. 1). Higher SVI index was also significantly associated with higher disease and poor health outcome rates in all models except for the high cholesterol model for the Mountain Region (ß_SVI Overall_ = 0.05, *p* = 0.678). Across regions, model results from unstandardized variables demonstrated similar effects as expected (Table 3, Supplemental Table 2).

## Discussion

The current study aimed to extend existing research by considering access to both traditional grocery stores and dollar stores as grocery sources and their associations with health given differential food quality. Previous work on food environments and health has often focused on traditional grocery store access measured primarily by proximity to a grocery store [1, 6]. This research typically excludes dollar stores. Overall, we found consistent beneficial health associations in US regions with better access to SQ grocery sources, while better access to LQ grocery sources (i.e., dollar stores) was generally associated with poorer health. It is important to highlight that even when aggregating certain socioeconomic factors represented by the overall SVI, these relationships remained significant. Hence, despite what might be an intuitive relationship between dollar store access and health, our consideration of non-traditional, potentially more affordable and accessible grocery sources (e.g., dollar stores) aligns with, and offers novel insight into, current multidimensional approaches to defining grocery environments [7, 10–12]. We further found that these positive associations between dollar store access and disease rates varied across regions and localities. Our work is noteworthy as it calls for further consideration of grocery source affordability and food offerings beyond the traditional grocery store. Given that LQ grocers tend to sell low cost, highly processed foods and allocate smaller amounts of space for nutrient-dense grocery items [22, 36, 42], we speculate that the health outcomes observed here likely reflect differences in food quality across grocery sources.

Our finding that access to LQ grocery sources is associated with poorer health underscores that dollar stores (i.e., a primary type of LQ grocery sources) have an association with health outcomes across regions and should be formally considered as part of the broader food environment. Previous work has found that some households that frequent dollar stores compensate for their purchase of non-nutrient-dense foods with healthier foods from other retail outlets, resulting in modest differences in diet relative to those who did not frequent dollar stores [15]. This compensatory approach may not be adopted broadly enough to overcome our observation of poorer health outcomes with greater access to LQ grocery sources. Moreover, in our study, health outcome differences were seen even when adjusting dollar store access with traditional grocery store access, implying that these associations are driven by LQ grocery independent of SQ grocery access, at least at a community level. The associations observed in this study may reflect a confluence of factors due to complexities within the built environment, meaning dollar stores could be a marker for neighborhood deprivation or market trends. Future work can address this ambiguity through such approaches as longitudinal design analyzing the temporal sequence between dollar store opening and changes in neighborhood socioeconomic factors.

While our results showed general statistical and visual trends were consistent across the US, there was notable variation across regions and within metro areas. For example, dollar store access and many cardiometabolic disease rates were highest across the South and Midwest regions of the US. However, visual representations across cities within these regions (Figs. 1 and 3, Supplemental Files) illustrated that higher dollar store access did not always coincide with areas of high cardiometabolic disease rates. Thus, future public health initiatives addressing food environment via grocery store quality metrics should not apply a one-size-fits-all solution and must instead consider regional and local variation.

In areas with high access to dollar stores, we propose that one solution is to increase food quality in those stores. This differs from past grocery environment interventions that predominately focus on increasing the number of traditional grocery stores in an area to improve accessibility [4]. Of note, previous work has shown that adding SQ grocery store access in areas with LQ grocery store access often fails to change community diet behaviors and overall health outcomes [2, 3, 5]. Also, previous community studies show that residents (including dollar store customers) desire more fresh options on dollar store shelves [43, 44]. Improving food quality in dollar stores through local policies or partnerships with local farmers may in turn improve health outcomes. This may be an important direction for future work.

Our study has notable limitations and critical considerations. Importantly, while we found significant associations between dollar store access and poor health outcomes, we did not test causality or broader structural drivers of the built environment such as systemic racism and classism within these communities. We acknowledge that dollar stores may function as a potential neighborhood exposure, a marker of broader structural disinvestment, or a product of poor access to unhealthy foods. Any causal interpretation of associations noted with dollar store presence can be complicated by underlying socioeconomic conditions and retail dynamics in a community. Our metric of socioeconomic factors was the SVI Overall, which includes variables such as poverty, median per capita income, and vehicle access. SVI Overall was beneficial for our tract-level analysis across broad US regions; however, it is limited to estimates at the scale of the census tract (an imperfect proxy at the neighborhood level [45, 46]) and cannot fully capture variability or inequity along additional axes of power and privilege (e.g., wealth inequality, housing instability). Future analyses could expand on this approach through incorporation of specific socioeconomic layers (e.g., walkability, public transit access, federal Supplemental Nutrition Assistance Program usage) important for robust grocery access within a given neighborhood or area. We did not account for other health behaviors that commonly co-occur with poor diet (e.g., smoking, sedentary lifestyle). These behaviors may explain why we found significant health associations between asthma and poor mental health, not just poor cardiometabolic health, with proximity to LQ dollar stores across the US. In some regions, asthma and/or mental health associations outpaced measures of cardiometabolic health such as high cholesterol. Comorbidity between asthma or poor mental health and the metabolic or cardiovascular conditions could contribute to these findings, in line with previous work [47, 48]. While we coded location points for major national and regional grocery store chains, we were unable to account for bodega style stores or independently owned stores that may only have one or a few locations. Our dataset only included large metropolitan areas that are part of the CDC 500 cities dataset [39]. Dollar stores hold particular importance in some rural areas where other grocery options are limited [16] and future work should elaborate on dollar store health associations in towns and rural communities. Additionally, grocery access in some urban areas that we analyzed may be more reasonably defined by walking rather than driving (e.g., Manhattan in New York City). Future testing could focus on specific high-density metro areas to consider both walking and driving buffers. Within our analysis, all dollar stores were classified as LQ grocery sources and all traditional grocery stores as SQ, an assumption that may introduce exposure misclassification. Both categories are heterogeneous, and this simplification may obscure meaningful variation in grocery environments across stores. Future work could develop granular, store-level classifications that better capture variability within and across retail types, including differences in product quality, affordability, and health-promoting provisions. Finally, the health outcome measurements were area-level estimates from the CDC [39]. This allowed us to examine large scale patterns across US regions and large cities; however, our study cannot directly address individual-level impacts. This reliance on area-level measures introduces risk of ecological inference [49], as associations observed at the census tract level may not reflect individual-level relationships. Additionally, census tract boundaries may not correspond to individuals’ lived activity spaces or actual food purchasing behaviors [50], resulting in potential spatial misalignment between measured environments and exposures [51, 52]. Future research on health behaviors and biomarkers should integrate individual-level data, mobility patterns, and activity-space approaches to capture a multidimensional approach of how people interact with grocery environments beyond administratively defined boundaries.

## Conclusions

This study highlights that access to quality grocery sources matters for health outcomes, even after considering an aggregate of socioeconomic factors. This detail has key implications for emerging multidimensional approaches for policy and related grocery environment definitions and interventions. Key implications include: (1) the need to classify dollar stores as grocery stores given their prevalence and associations with poor health across the US and each region, and (2) considering policy to improve the quality of food options at dollar stores through increased variety and number of fresh and nutrient-dense food products. While strategies to improve the quality of food at dollar stores would likely vary between (and even within) metro areas, it is important to retain dollar stores due to their pervasiveness and use across disadvantaged communities. Dollar stores serve as an important grocery source option across communities, and our study suggests that they also play a pivotal role in health.

## Supplementary Information

Supplementary Material 1.

Supplementary Material 2.

Supplementary Material 3.

## Data Availability

The datasets used and/or analyzed during the current study are available from the corresponding author on reasonable request.

## Abbreviations

US: United States
SQ: standard quality
LQ: low quality
CDC: Centers for Disease Control
USDA: United States Department of Agriculture
SVI: Social Vulnerability Index
