# Supplementary material for: Beyond proximity: how food quality in dollar and grocery stores influences health
Source: BMC Public Health. 2026 Jun 18;26:3268. doi: 10.1186/s12889-026-28145-y (PMC13746574; doi:10.1186/s12889-026-28145-y)
Supplement: Supplementary file 1 [file 12889_2026_28145_MOESM1_ESM.pdf]

Albuquerque, New Mexico (Mountain Region)

A) B)

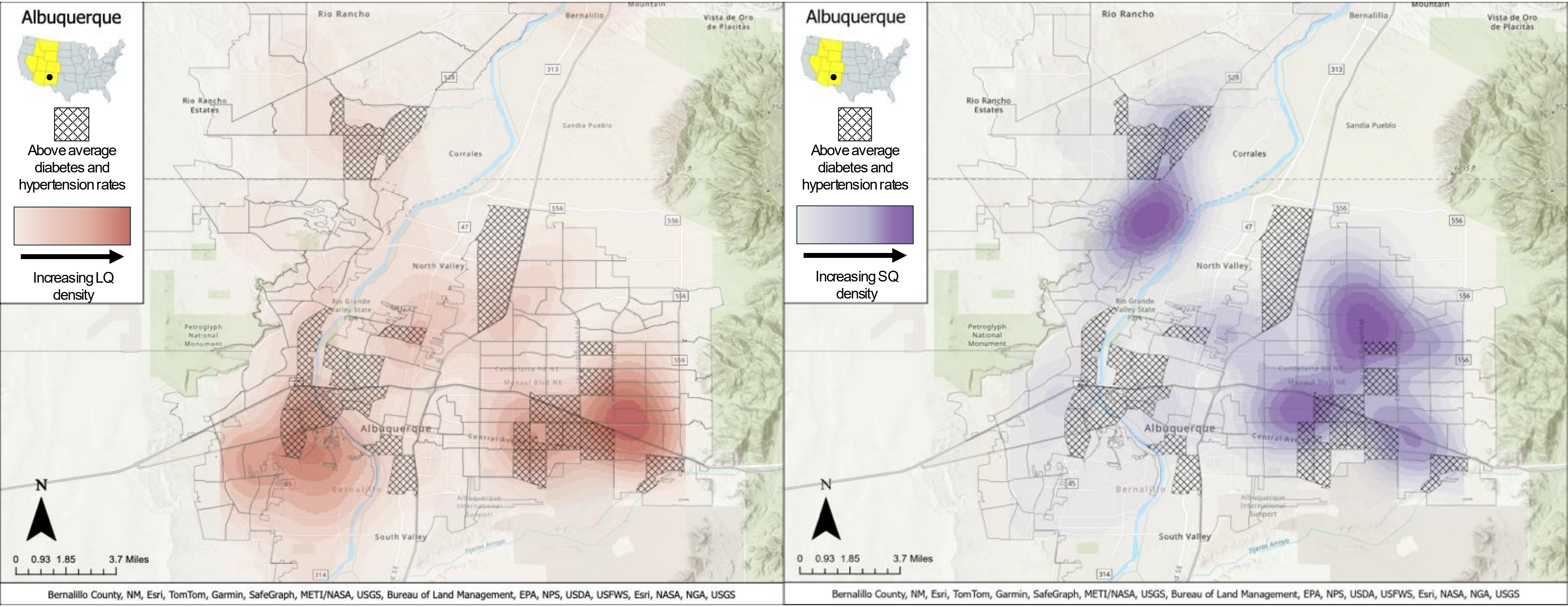

Side by side kernel analysis overlay with hatched tracts for the city of Albuquerque, New Mexico. Hatched tracts represent above US average diabetes and hypertension rates. A) Red kernel shaded areas are LQ (dollar store) dense areas. B) Purple kernel shaded areas are SQ (traditional grocery store) dense areas. LQ = Low Quality, SQ = Standard Quality

Atlanta, Georgia (South Atlantic Region)

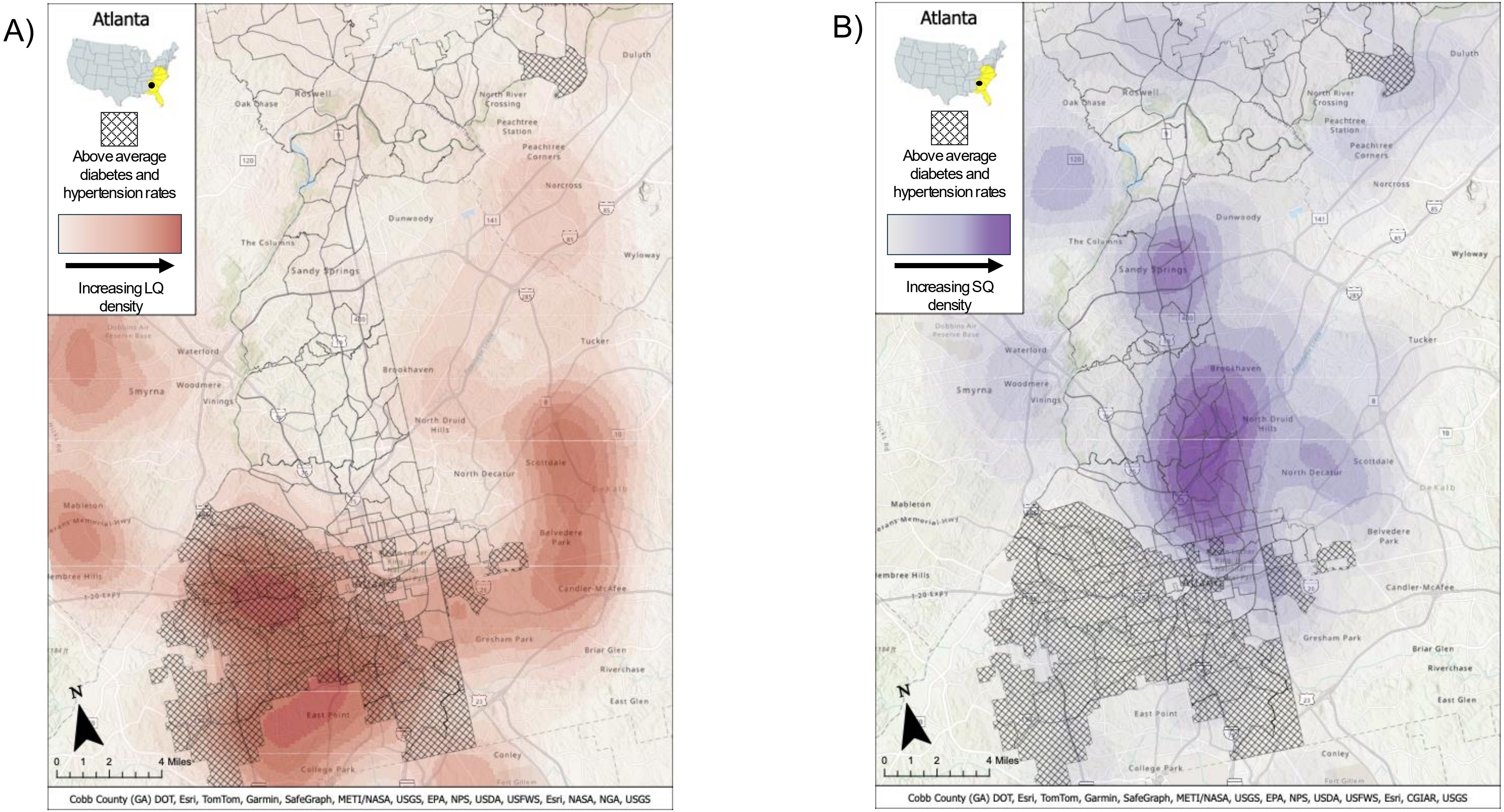

Side by side kernel analysis overlay with hatched tracts for the city of Atlanta, Georgia. Hatched tracts represent above US average diabetes and hypertension rates. A) Red kernel shaded areas are LQ (dollar store) dense areas. B) Purple kernel shaded areas are SQ (traditional grocery store) dense areas. LQ = Low Quality, SQ = Standard Quality

Austin, Texas (South Region)

A) B)

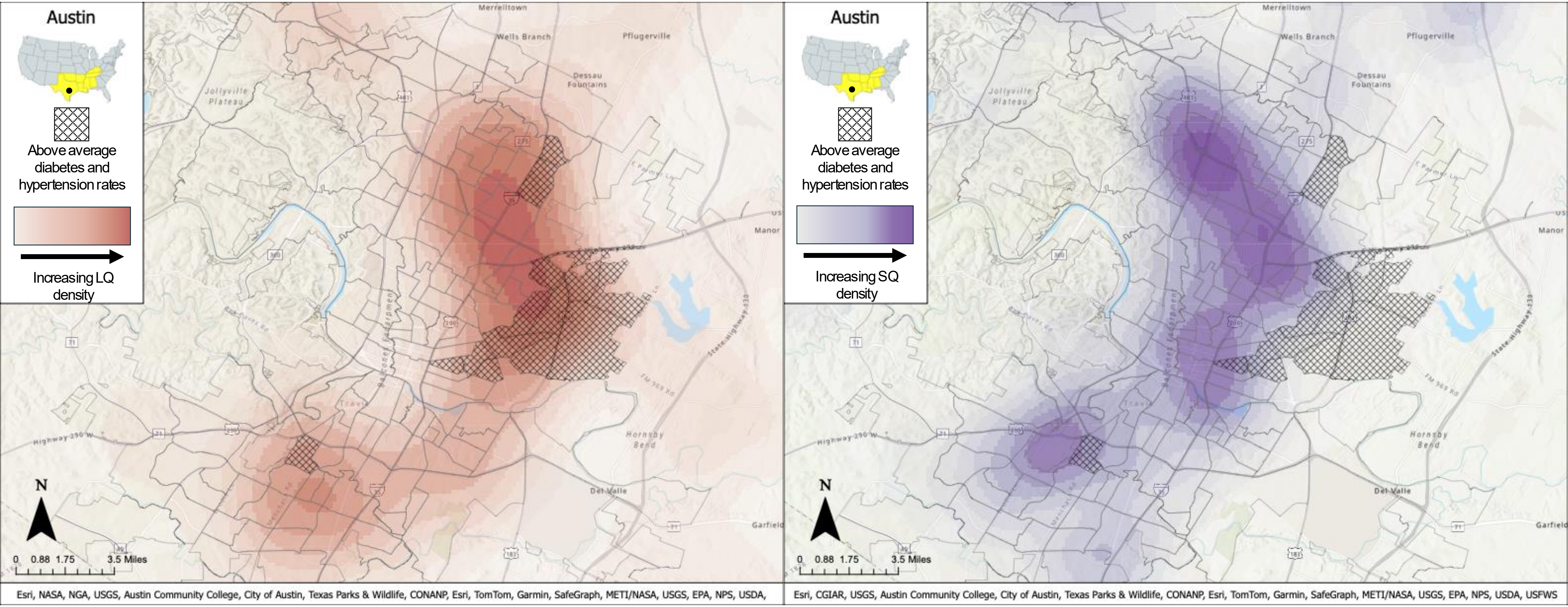

Side by side kernel analysis overlay with hatched tracts for the city of Austin, Texas. Hatched tracts represent above US average diabetes and hypertension rates. A) Red kernel shaded areas are LQ (dollar store) dense areas. B) Purple kernel shaded areas are SQ (traditional grocery store) dense areas. LQ = Low Quality, SQ = Standard Quality

Baltimore, Maryland (Northeast Region)

A) B)

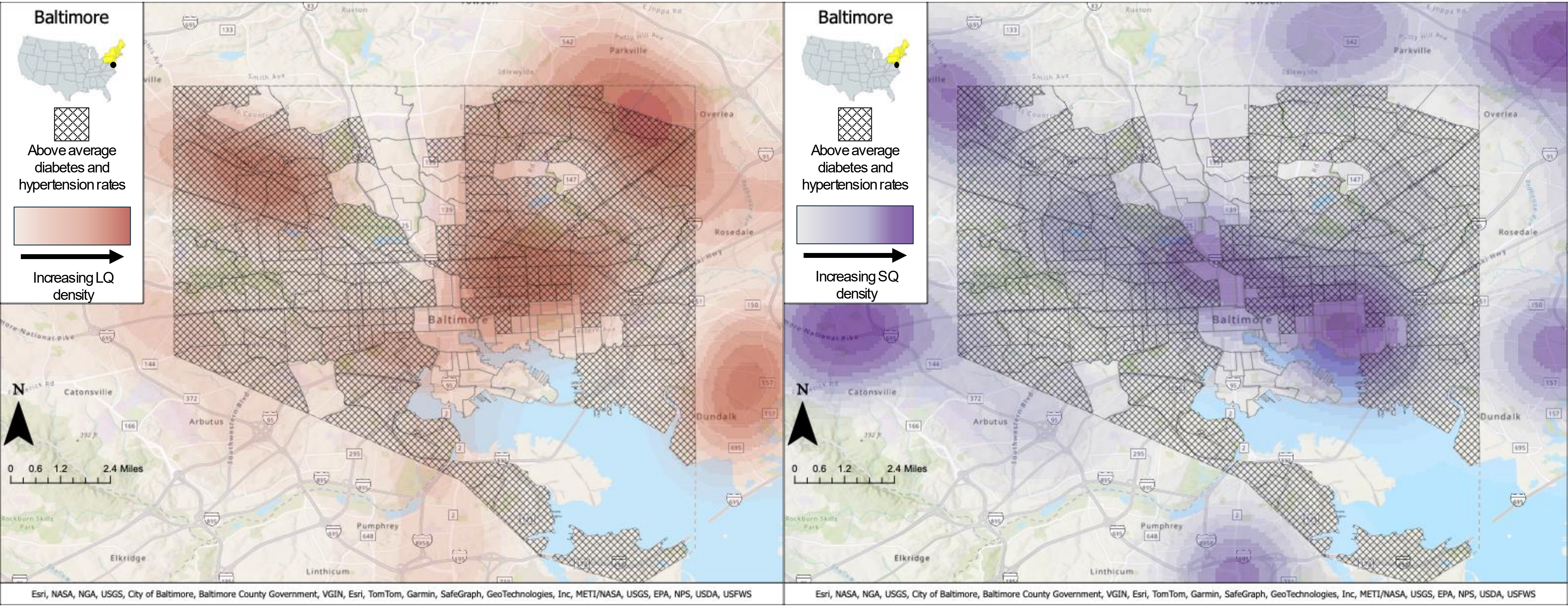

Side by side kernel analysis overlay with hatched tracts for the city of Baltimore, Maryland. Hatched tracts represent above US average diabetes and hypertension rates. A) Red kernel shaded areas are LQ (dollar store) dense areas. B) Purple kernel shaded areas are SQ (traditional grocery store) dense areas. LQ = Low Quality, SQ = Standard Quality

Birmingham, Alabama (South Region)

A) B)

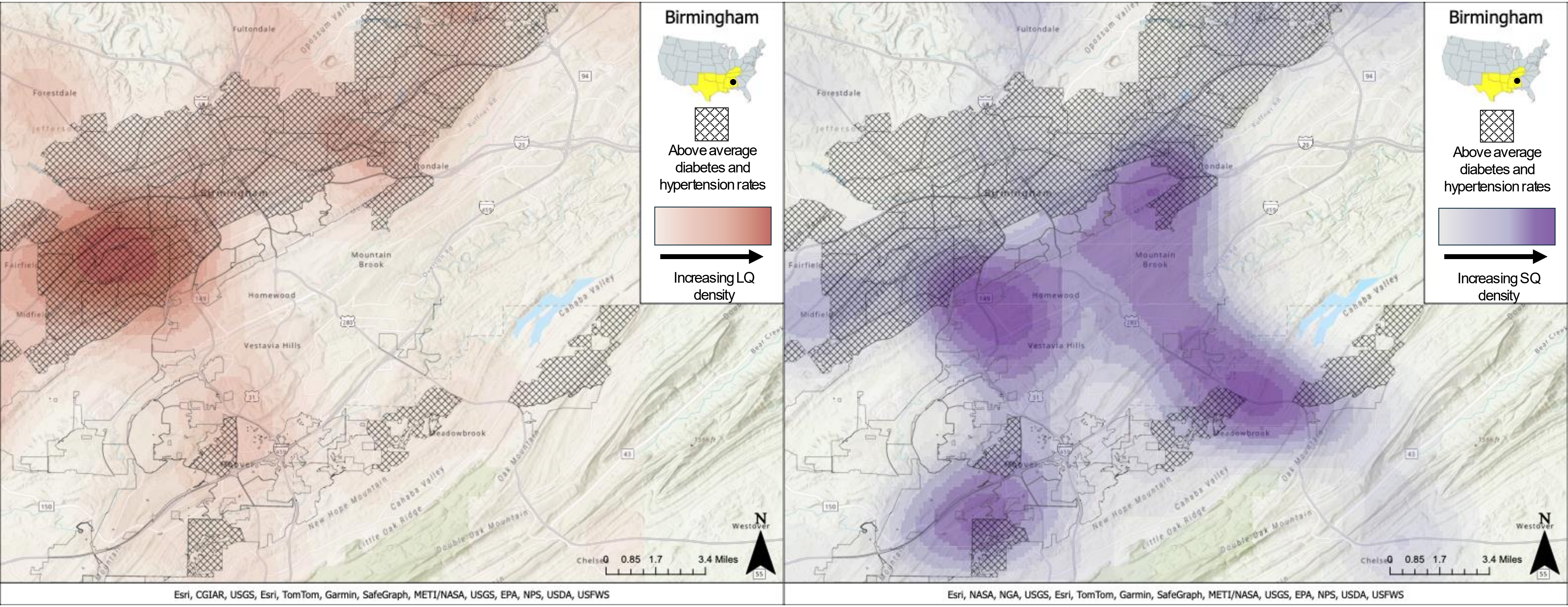

Side by side kernel analysis overlay with hatched tracts for the city of Birmingham, Alabama. Hatched tracts represent above US average diabetes and hypertension rates. A) Red kernel shaded areas are LQ (dollar store) dense areas. B) Purple kernel shaded areas are SQ (traditional grocery store) dense areas. LQ = Low Quality, SQ = Standard Quality

Boise, Idaho (Mountain Region)

A) B)

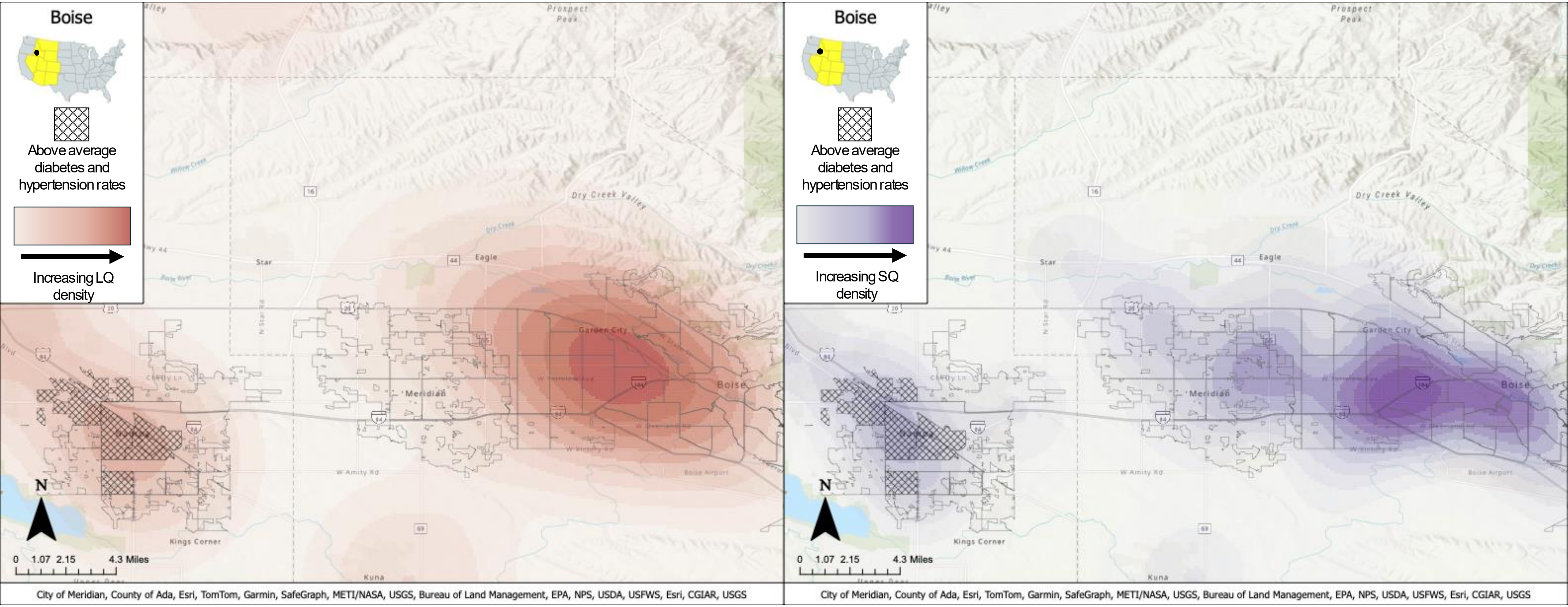

Side by side kernel analysis overlay with hatched tracts for the city of Boise, Idaho. Hatched tracts represent above US average diabetes and hypertension rates. A) Red kernel shaded areas are LQ (dollar store) dense areas. B) Purple kernel shaded areas are SQ (traditional grocery store) dense areas. LQ = Low Quality, SQ = Standard Quality

Boston, Massachusetts (Northeast Region)

A)

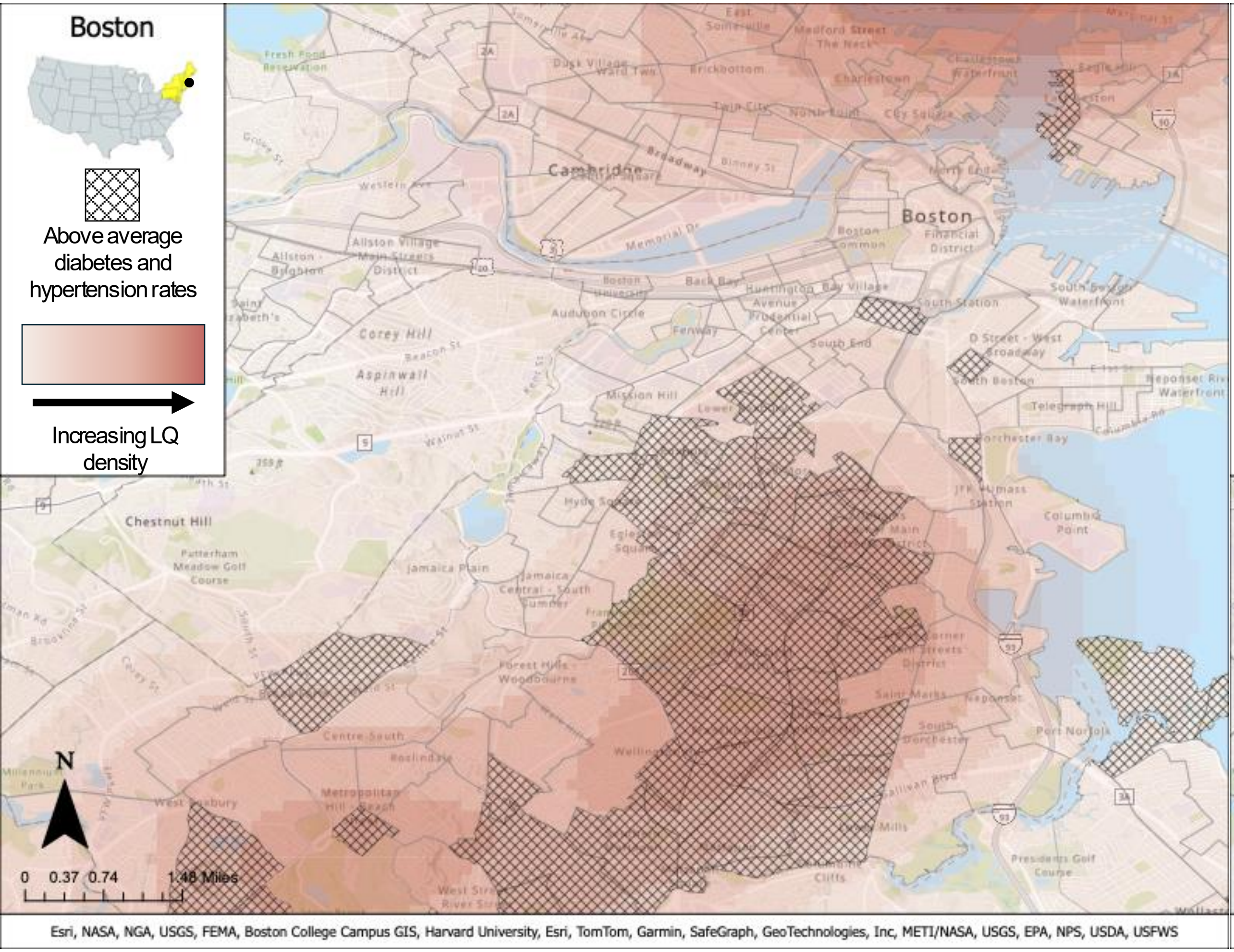

B)

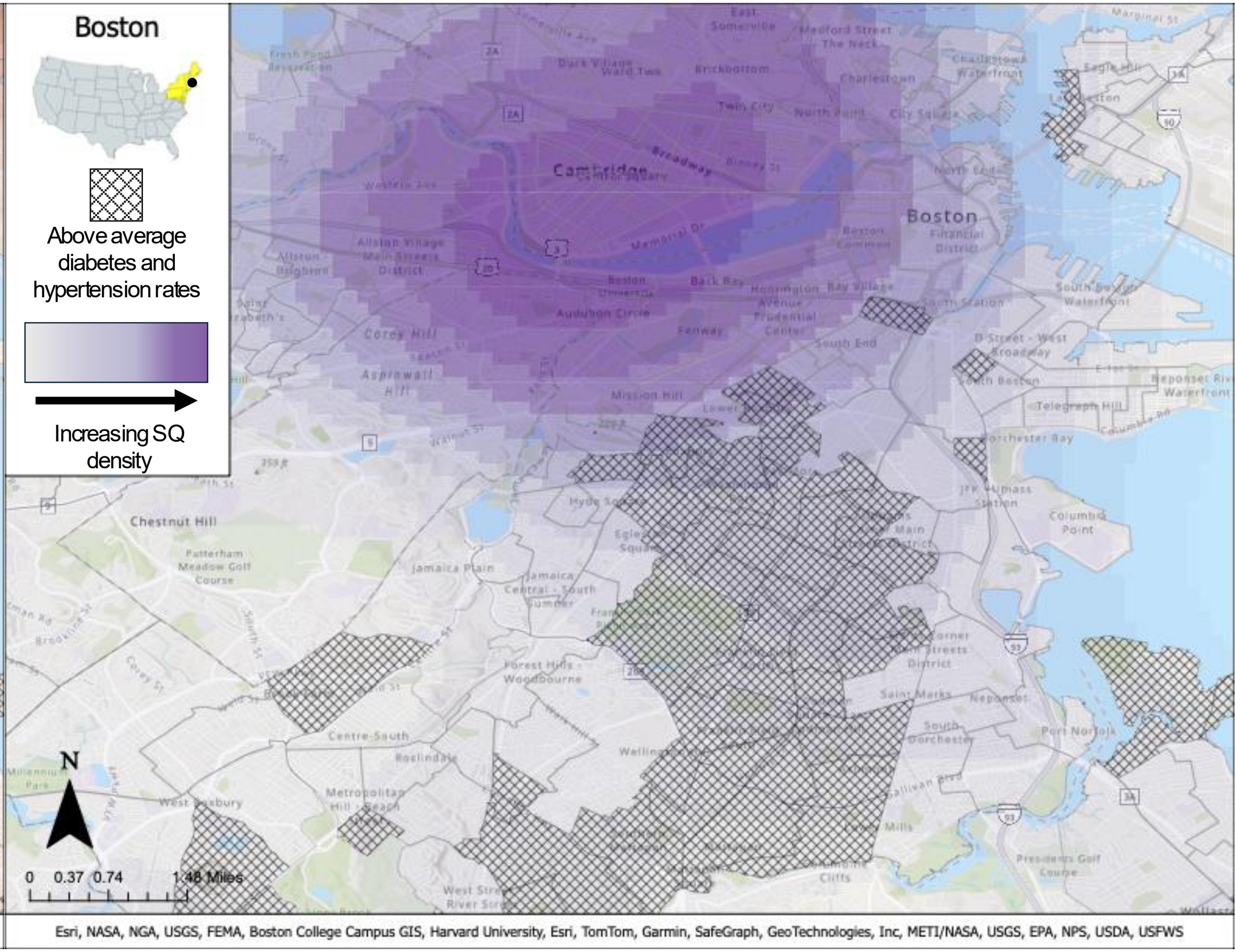

Side by side kernel analysis overlay with hatched tracts for the city of Boston, Massachusetts. Hatched tracts represent above US average diabetes and hypertension rates. A) Red kernel shaded areas are LQ (dollar store) dense areas. B) Purple kernel shaded areas are SQ (traditional grocery store) dense areas. LQ = Low Quality, SQ = Standard Quality

Charleston, South Carolina (South Atlantic Region)

A) B)

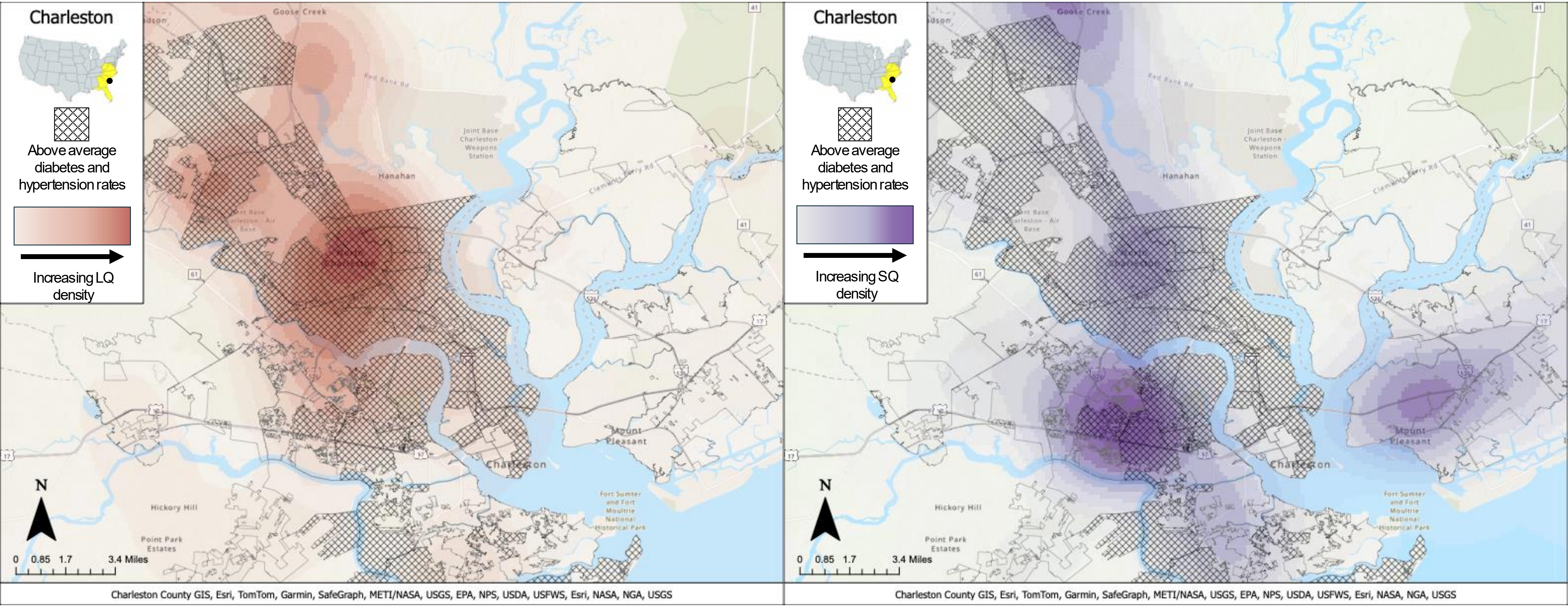

Side by side kernel analysis overlay with hatched tracts for the city of Charleston, South Carolina. Hatched tracts represent above US average diabetes and hypertension rates. A) Red kernel shaded areas are LQ (dollar store) dense areas. B) Purple kernel shaded areas are SQ (traditional grocery store) dense areas. LQ = Low Quality, SQ = Standard Quality

Charlotte, North Carolina (South Atlantic Region)

A) B)

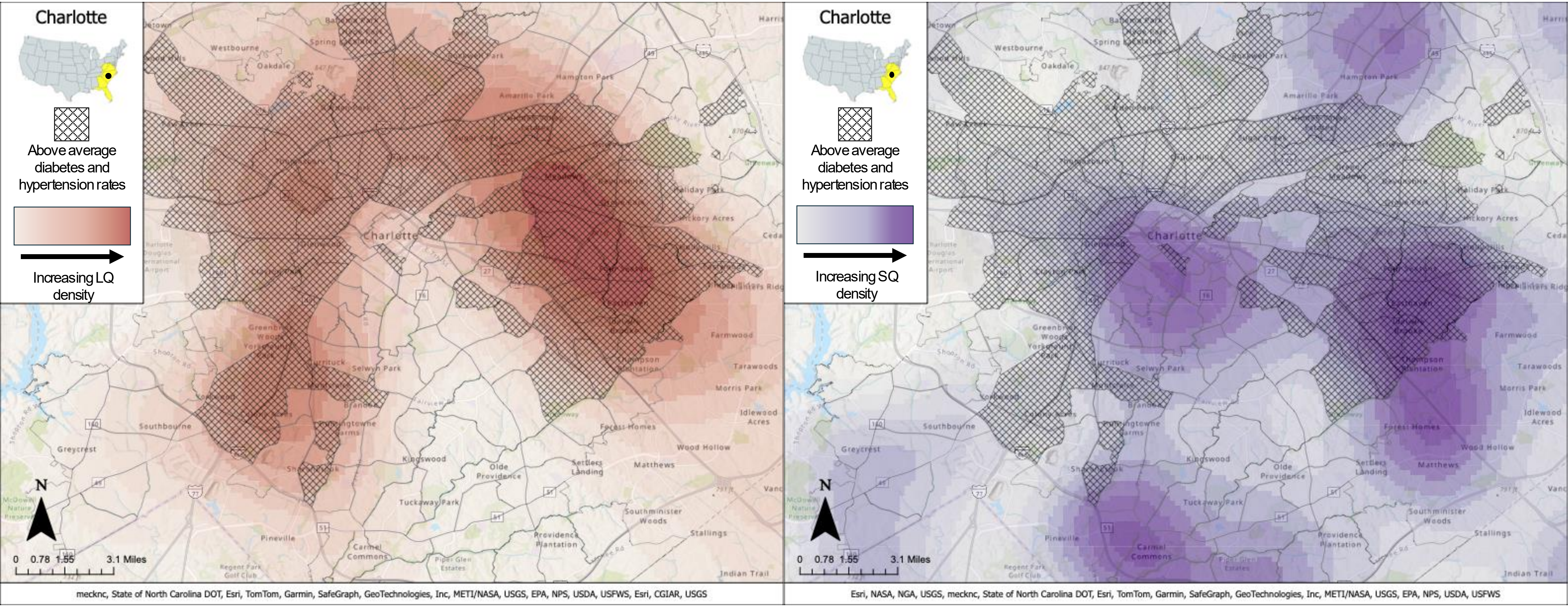

Side by side kernel analysis overlay with hatched tracts for the city of Charlotte, North Carolina. Hatched tracts represent above US average diabetes and hypertension rates. A) Red kernel shaded areas are LQ (dollar store) dense areas. B) Purple kernel shaded areas are SQ (traditional grocery store) dense areas. LQ = Low Quality, SQ = Standard Quality

Cincinnati, Ohio (Midwest Region)

A) B)

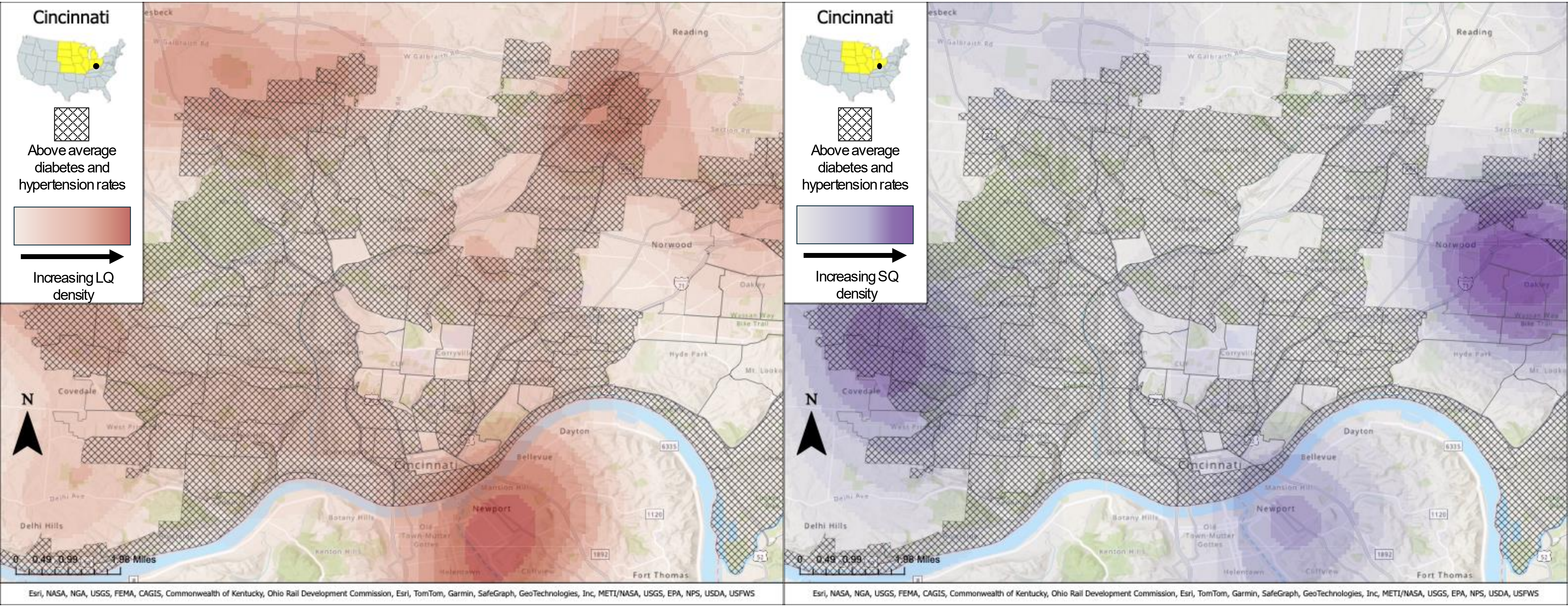

Side by side kernel analysis overlay with hatched tracts for the city of Cincinnati, Ohio. Hatched tracts represent above US average diabetes and hypertension rates. A) Red kernel shaded areas are LQ (dollar store) dense areas. B) Purple kernel shaded areas are SQ (traditional grocery store) dense areas. LQ = Low Quality, SQ = Standard Quality

Cleveland, Ohio (Midwest Region)

A) B)

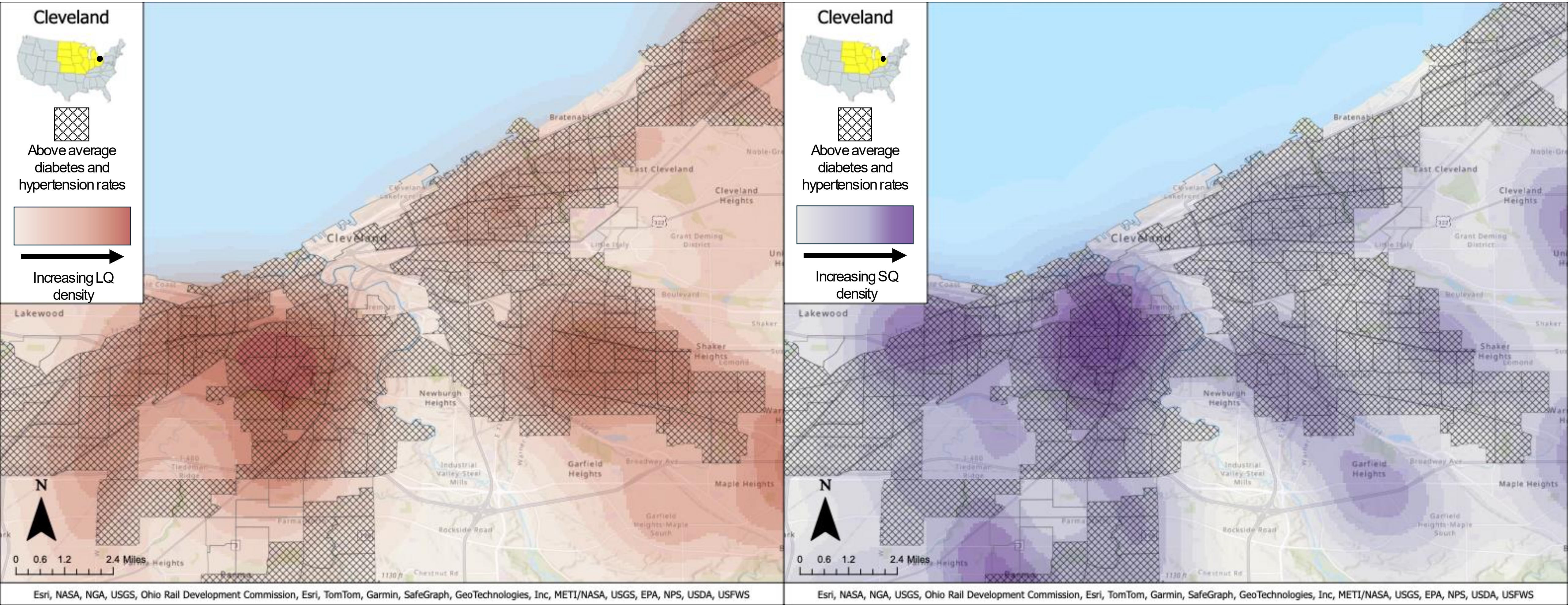

Side by side kernel analysis overlay with hatched tracts for the city of Cleveland, Ohio. Hatched tracts represent above US average diabetes and hypertension rates. A) Red kernel shaded areas are LQ (dollar store) dense areas. B) Purple kernel shaded areas are SQ (traditional grocery store) dense areas. LQ = Low Quality, SQ = Standard Quality

Colorado Springs, Colorado (Mountain Region)

A) B)

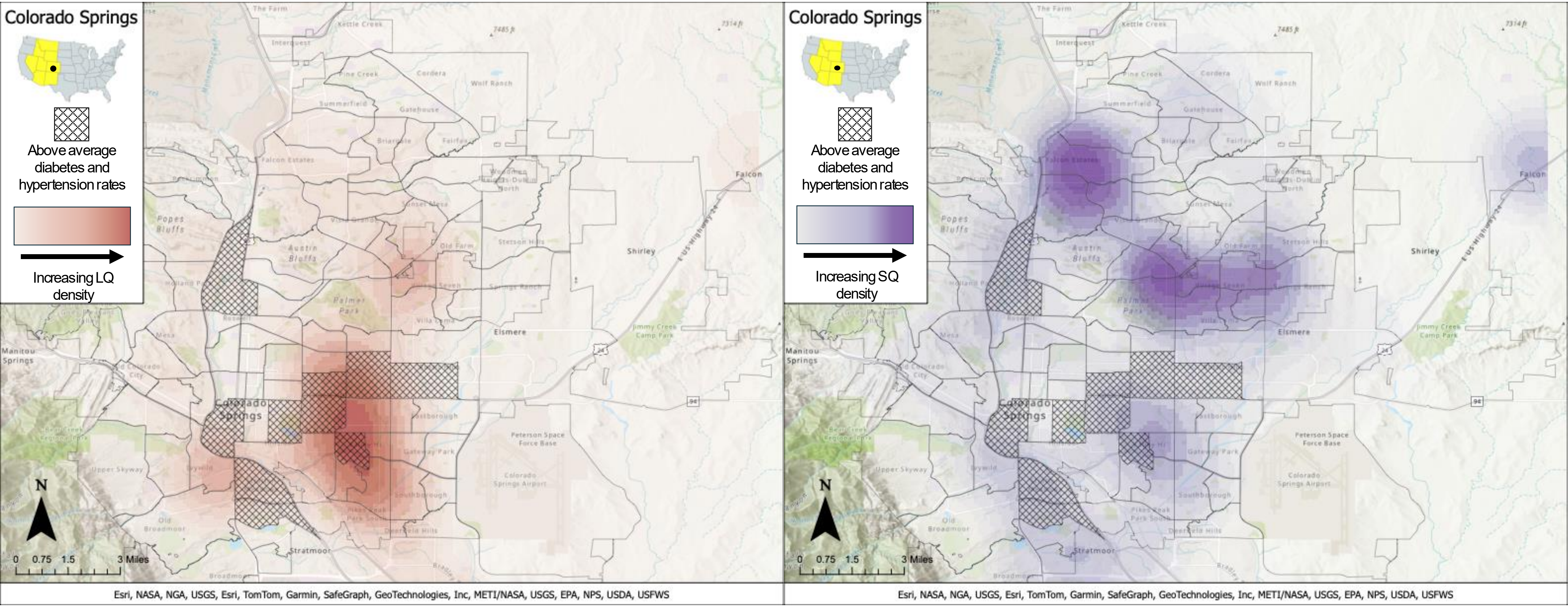

Side by side kernel analysis overlay with hatched tracts for the city of Colorado Springs, Colorado. Hatched tracts represent above US average diabetes and hypertension rates. A) Red kernel shaded areas are LQ (dollar store) dense areas. B) Purple kernel shaded areas are SQ (traditional grocery store) dense areas. LQ = Low Quality, SQ = Standard Quality

Columbus, Ohio (Midwest Region)

A) B)

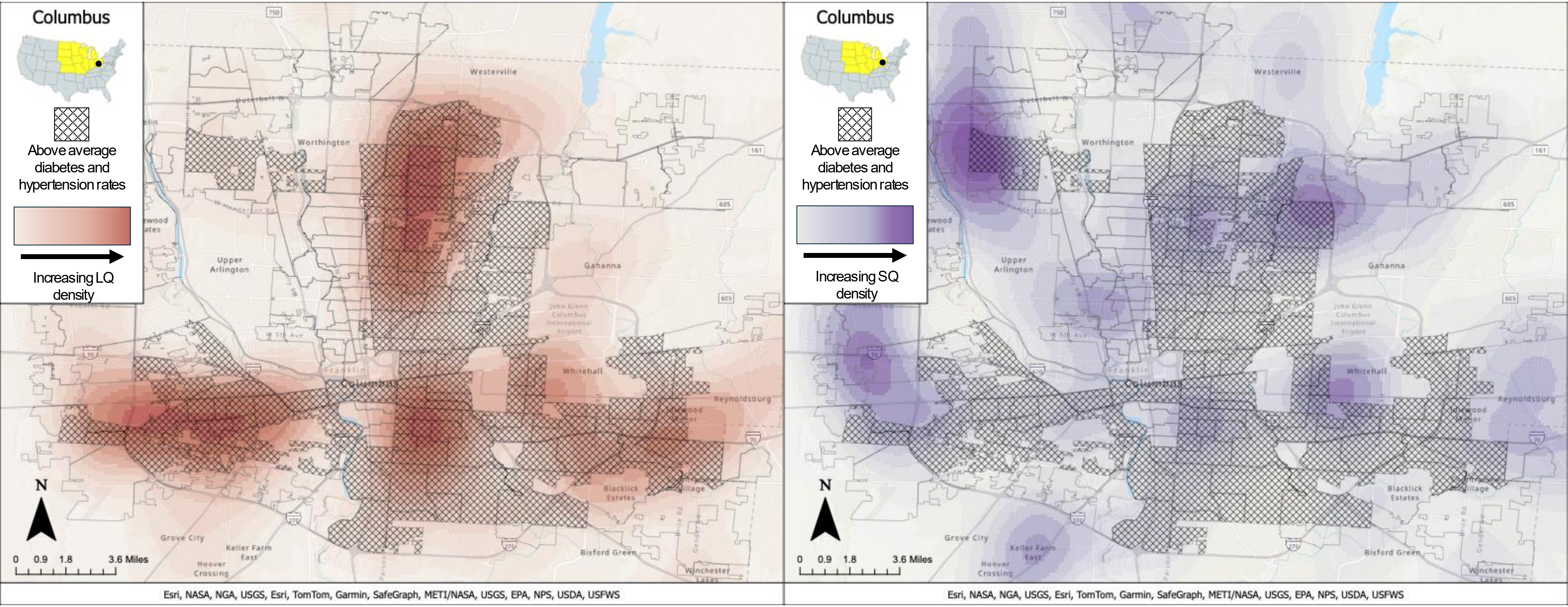

Side by side kernel analysis overlay with hatched tracts for the city of Columbus, Ohio. Hatched tracts represent above US average diabetes and hypertension rates. A) Red kernel shaded areas are LQ (dollar store) dense areas. B) Purple kernel shaded areas are SQ (traditional grocery store) dense areas. LQ = Low Quality, SQ = Standard Quality

Dallas, Texas (South Region)

A) B)

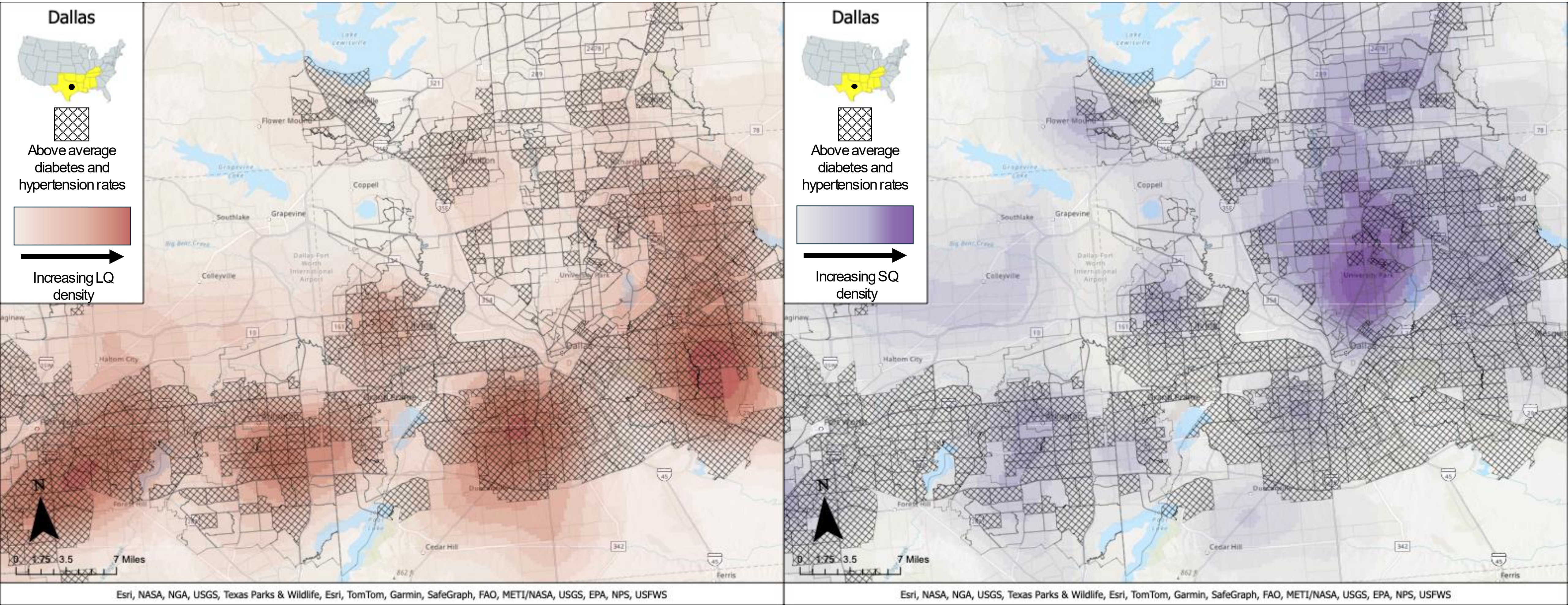

Side by side kernel analysis overlay with hatched tracts for the metro of Dallas, Texas. Hatched tracts represent above US average diabetes and hypertension rates. A) Red kernel shaded areas are LQ (dollar store) dense areas. B) Purple kernel shaded areas are SQ (traditional grocery store) dense areas. LQ = Low Quality, SQ = Standard Quality

Denver, Colorado (Mountain Region)

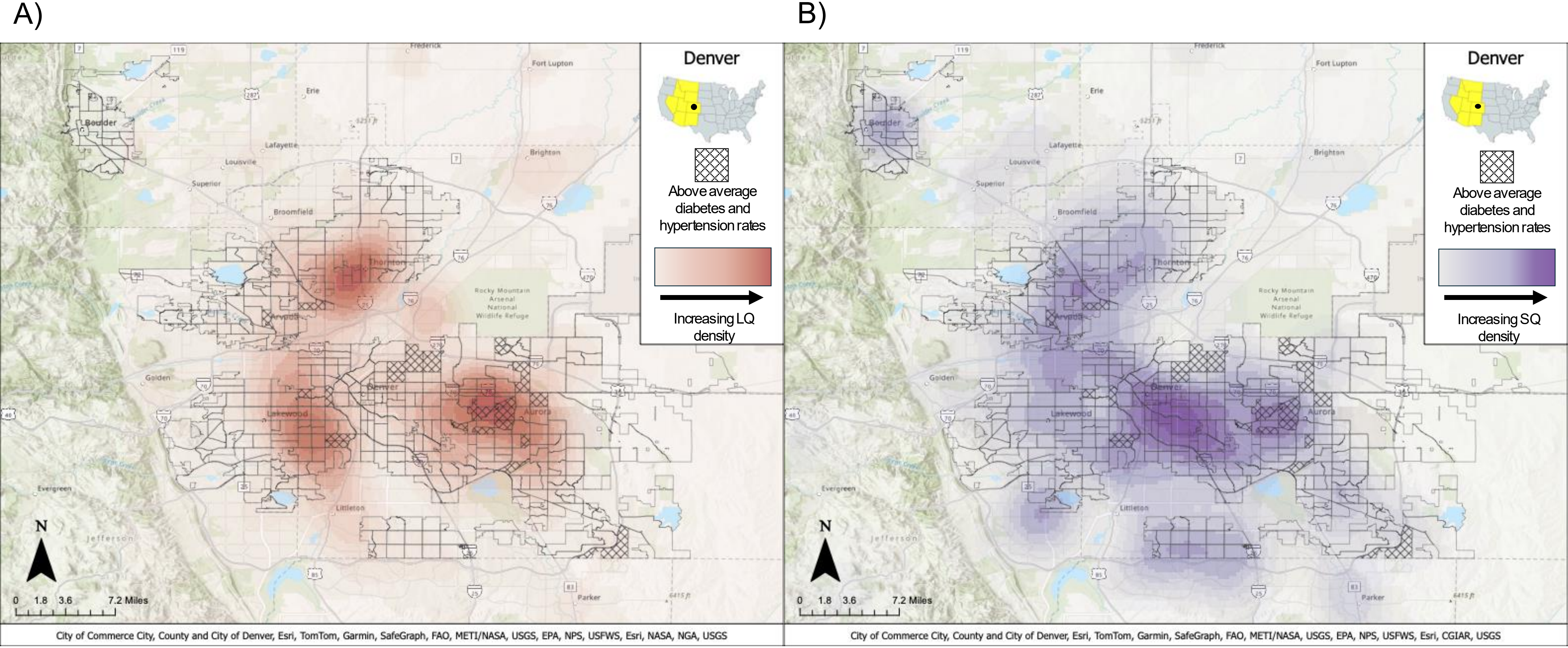

Side by side kernel analysis overlay with hatched tracts for the metro of Denver, Colorado. Hatched tracts represent above US average diabetes and hypertension rates. A) Red kernel shaded areas are LQ (dollar store) dense areas. B) Purple kernel shaded areas are SQ (traditional grocery store) dense areas. LQ = Low Quality, SQ = Standard Quality

Des Moines, Iowa (Midwest Region)

A) B)

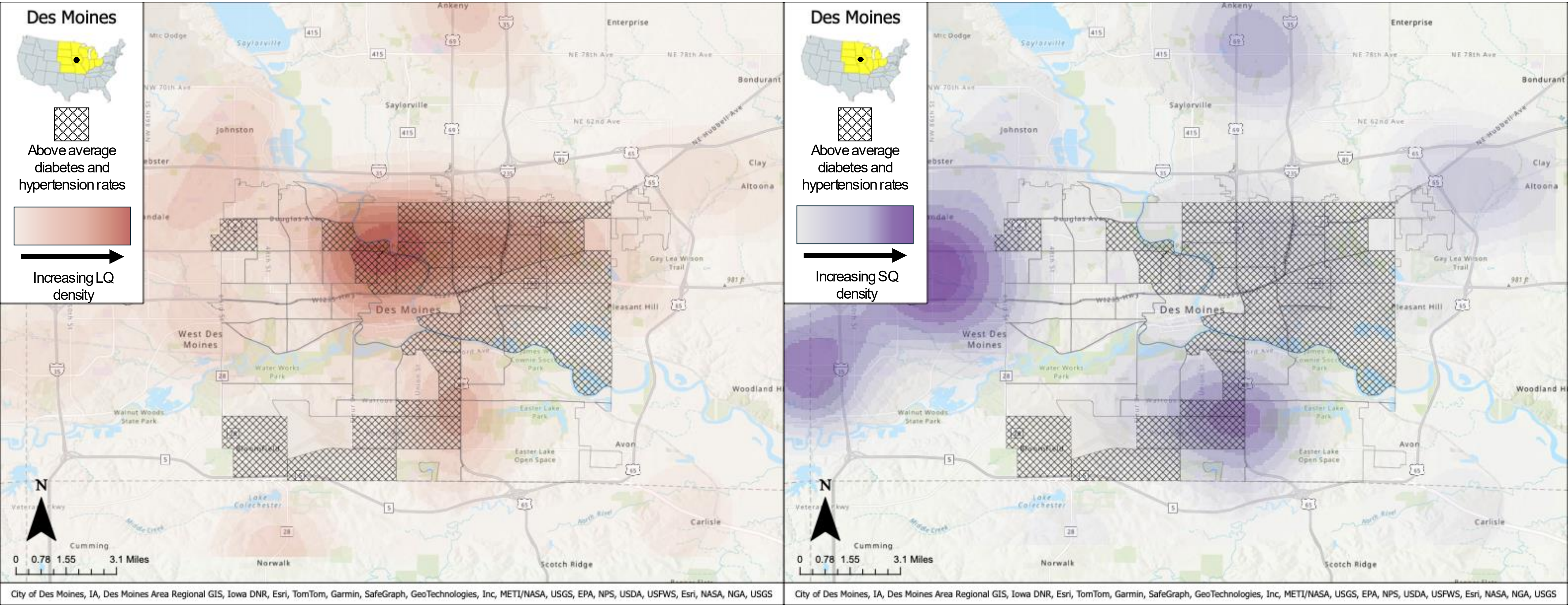

Side by side kernel analysis overlay with hatched tracts for the city of Des Moines, Iowa. Hatched tracts represent above US average diabetes and hypertension rates. A) Red kernel shaded areas are LQ (dollar store) dense areas. B) Purple kernel shaded areas are SQ (traditional grocery store) dense areas. LQ = Low Quality, SQ = Standard Quality

Detroit, Michigan (Midwest Region)

A)

B)

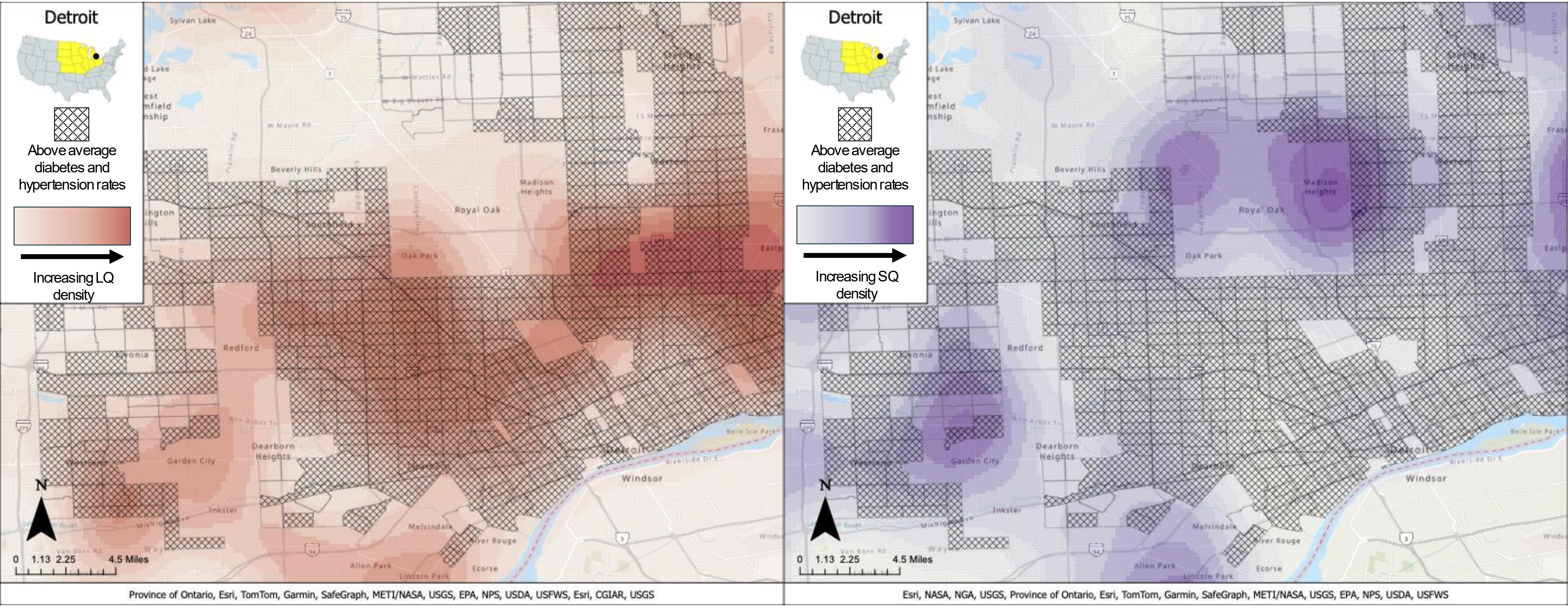

Side by side kernel analysis overlay with hatched tracts for the city of Detroit, Michigan. Hatched tracts represent above US average diabetes and hypertension rates. A) Red kernel shaded areas are LQ (dollar store) dense areas. B) Purple kernel shaded areas are SQ (traditional grocery store) dense areas. LQ = Low Quality, SQ = Standard Quality

El Paso, Texas (South Region)

A)

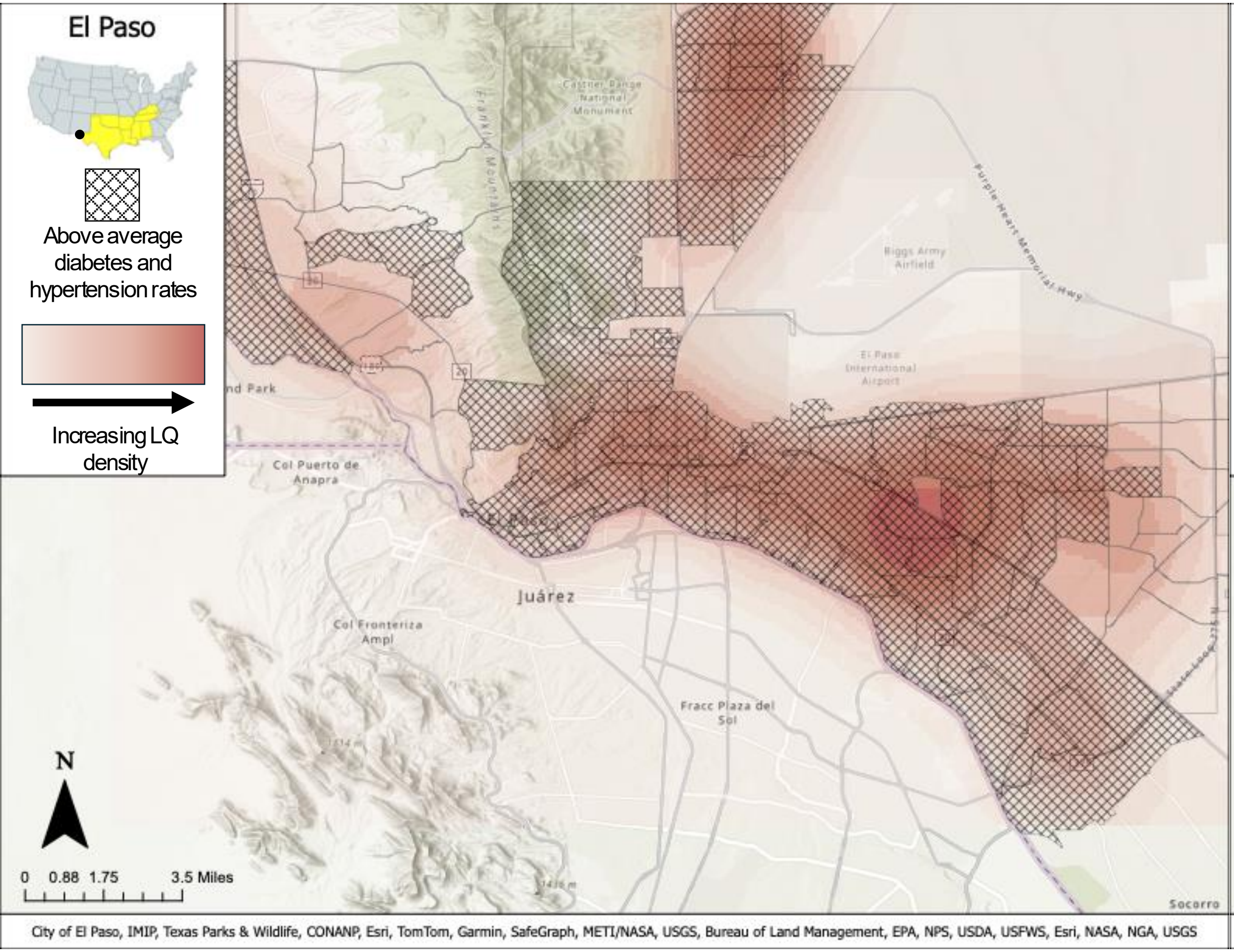

B)

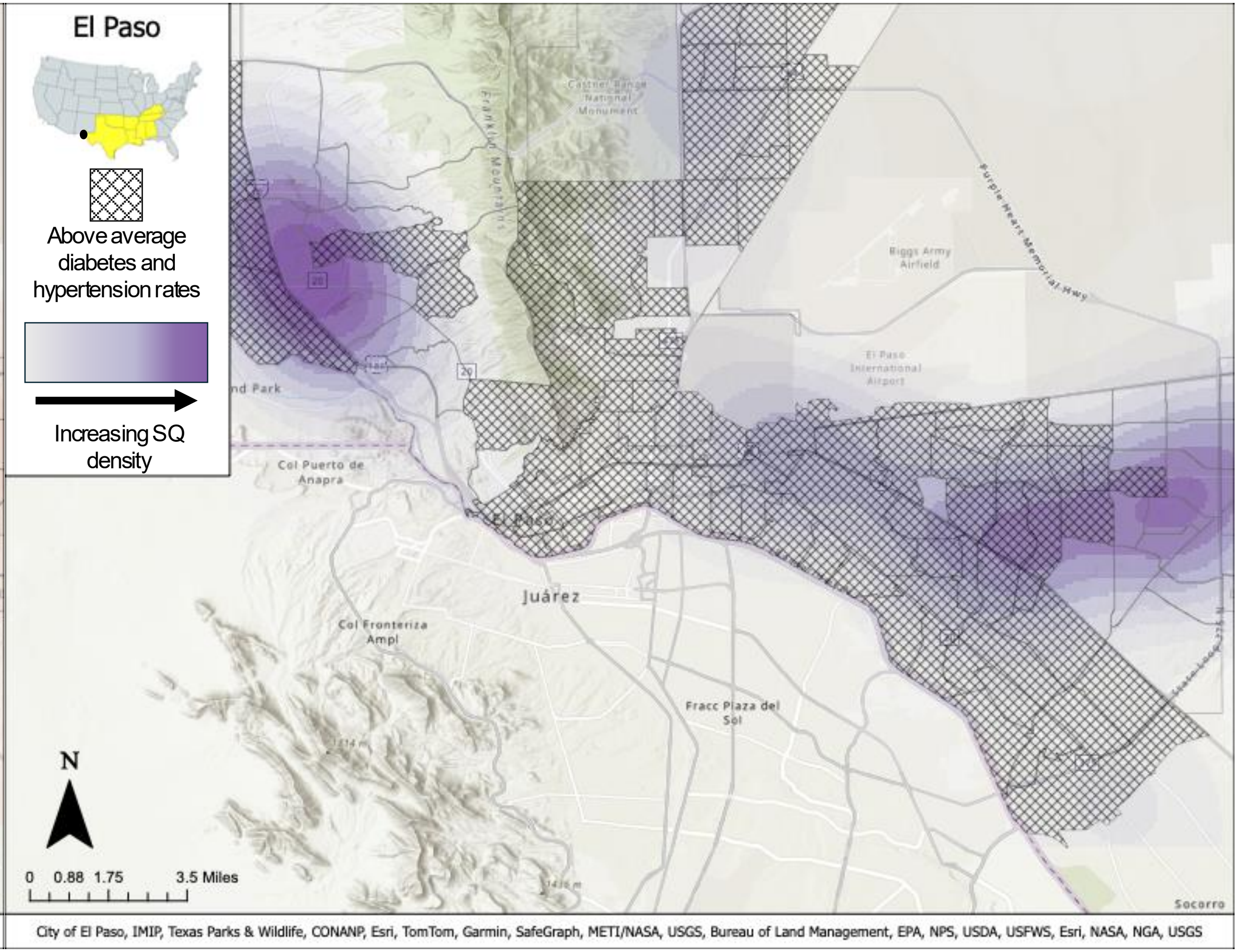

Side by side kernel analysis overlay with hatched tracts for the city of El Paso, Texas. Hatched tracts represent above US average diabetes and hypertension rates. A) Red kernel shaded areas are LQ (dollar store) dense areas. B) Purple kernel shaded areas are SQ (traditional grocery store) dense areas. LQ = Low Quality, SQ = Standard Quality

Houston, Texas (South Region)

A) B)

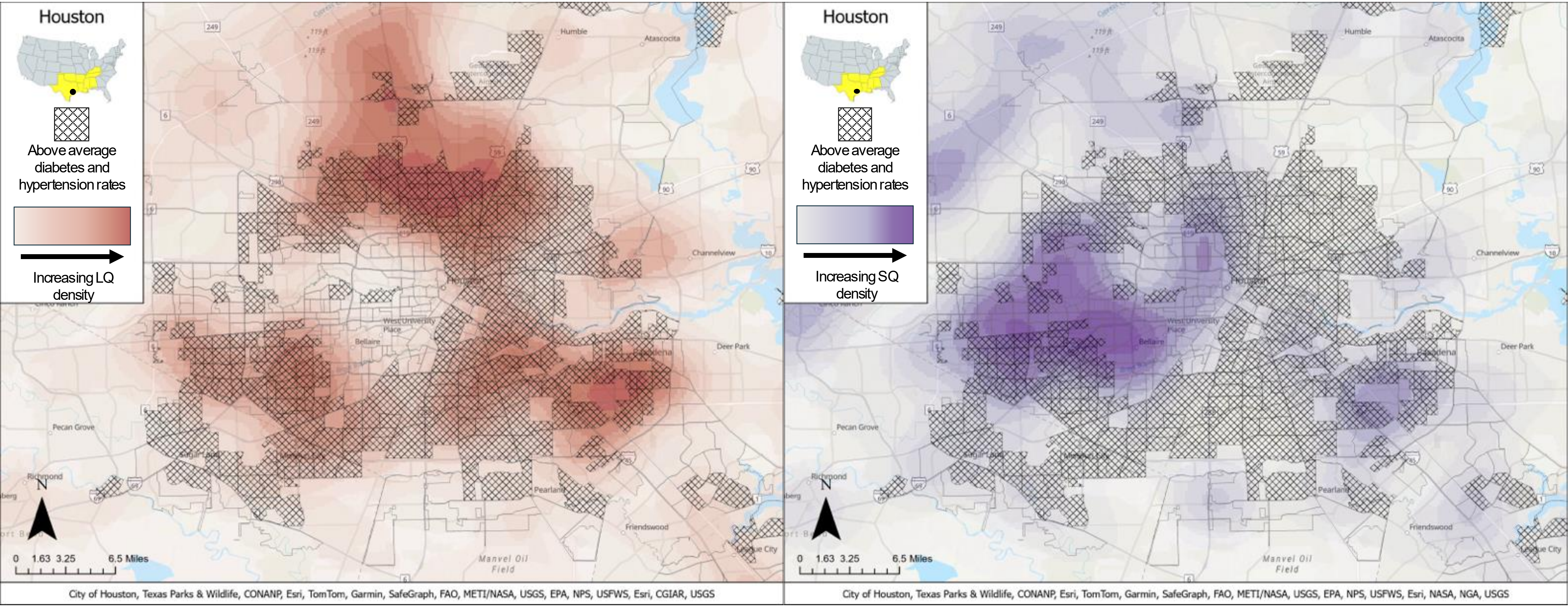

Side by side kernel analysis overlay with hatched tracts for the city of Houston, Texas. Hatched tracts represent above US average diabetes and hypertension rates. A) Red kernel shaded areas are LQ (dollar store) dense areas. B) Purple kernel shaded areas are SQ (traditional grocery store) dense areas. LQ = Low Quality, SQ = Standard Quality

Indianapolis, Indiana (Midwest Region)

A)

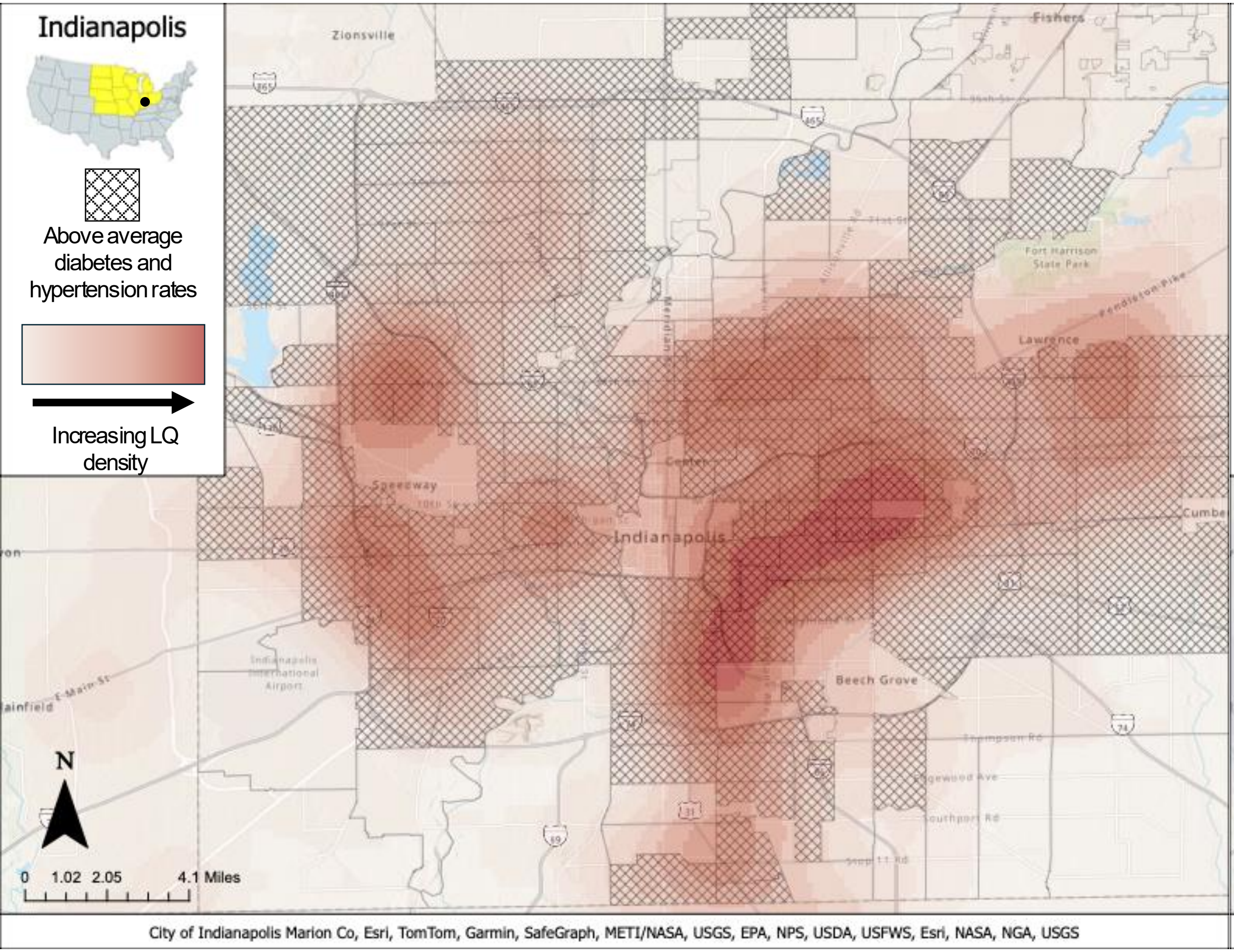

B)

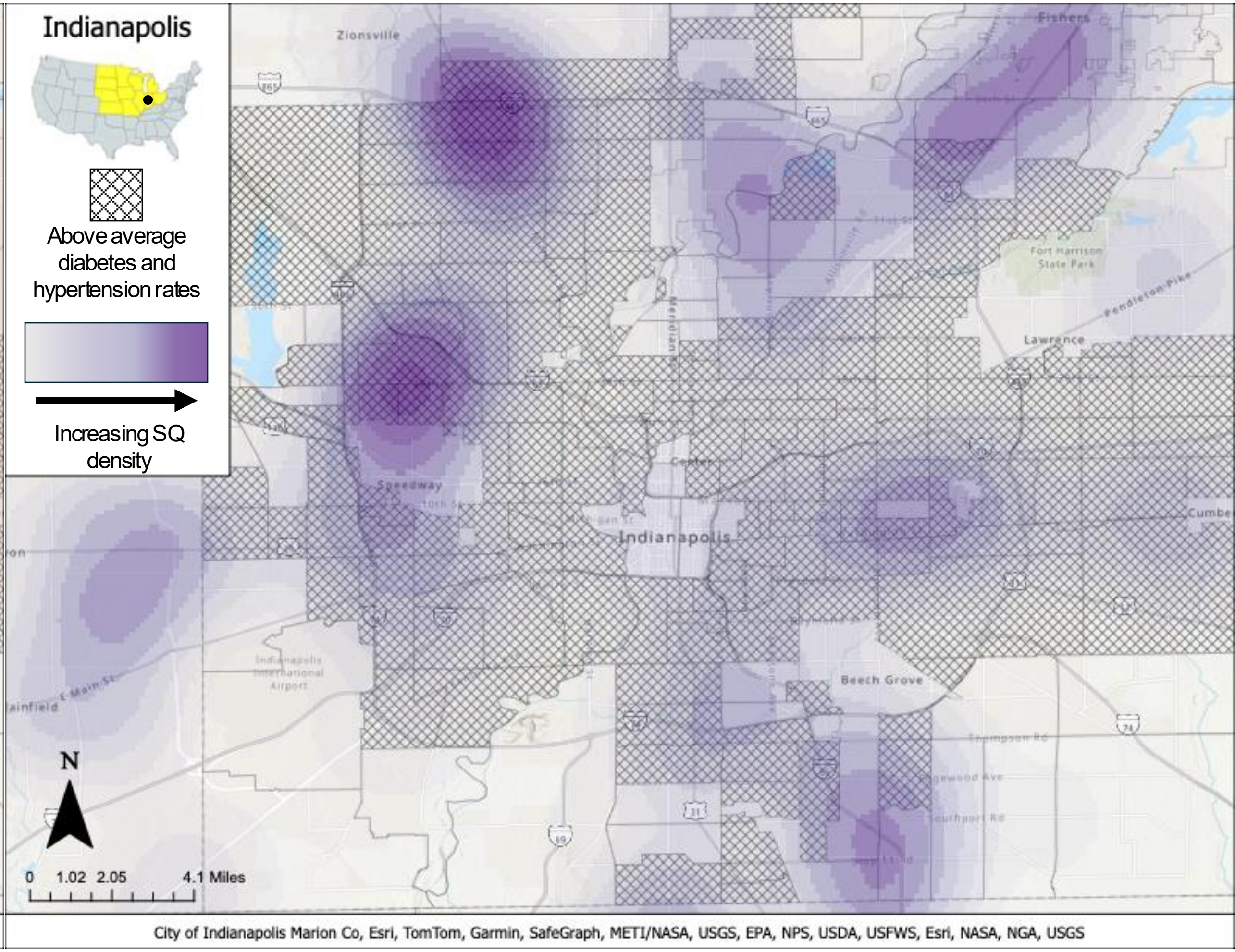

Side by side kernel analysis overlay with hatched tracts for the city of Indianapolis, Indiana. Hatched tracts represent above US average diabetes and hypertension rates. A) Red kernel shaded areas are LQ (dollar store) dense areas. B) Purple kernel shaded areas are SQ (traditional grocery store) dense areas. LQ = Low Quality, SQ = Standard Quality

Inland Empire, California (Pacific Region)

A) B)

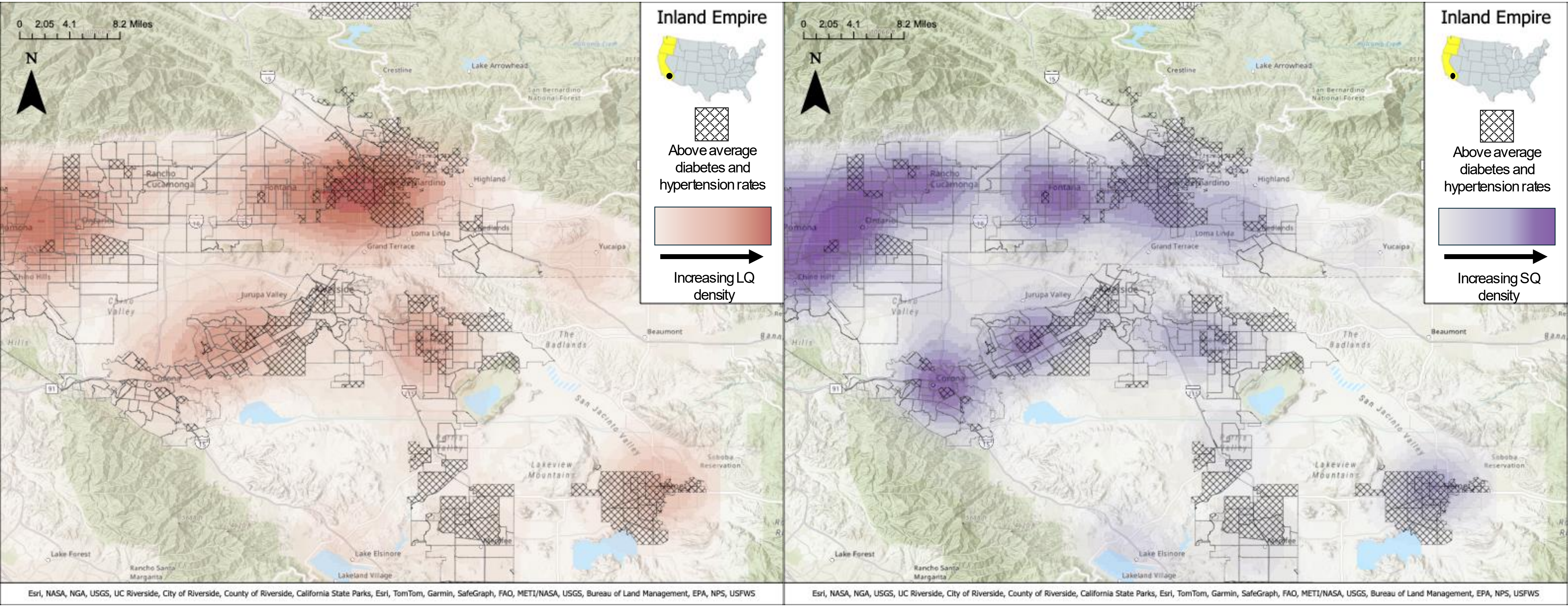

Side by side kernel analysis overlay with hatched tracts for cities in the Inland Empire Region, California (Riverside, San Bernadino, Ontario, Corona and surrounding areas). Hatched tracts represent above US average diabetes and hypertension rates. A) Red kernel shaded areas are LQ (dollar store) dense areas. B) Purple kernel shaded areas are SQ (traditional grocery store) dense areas. LQ = Low Quality, SQ = Standard Quality

Jackson, Mississippi (South Region)

A)

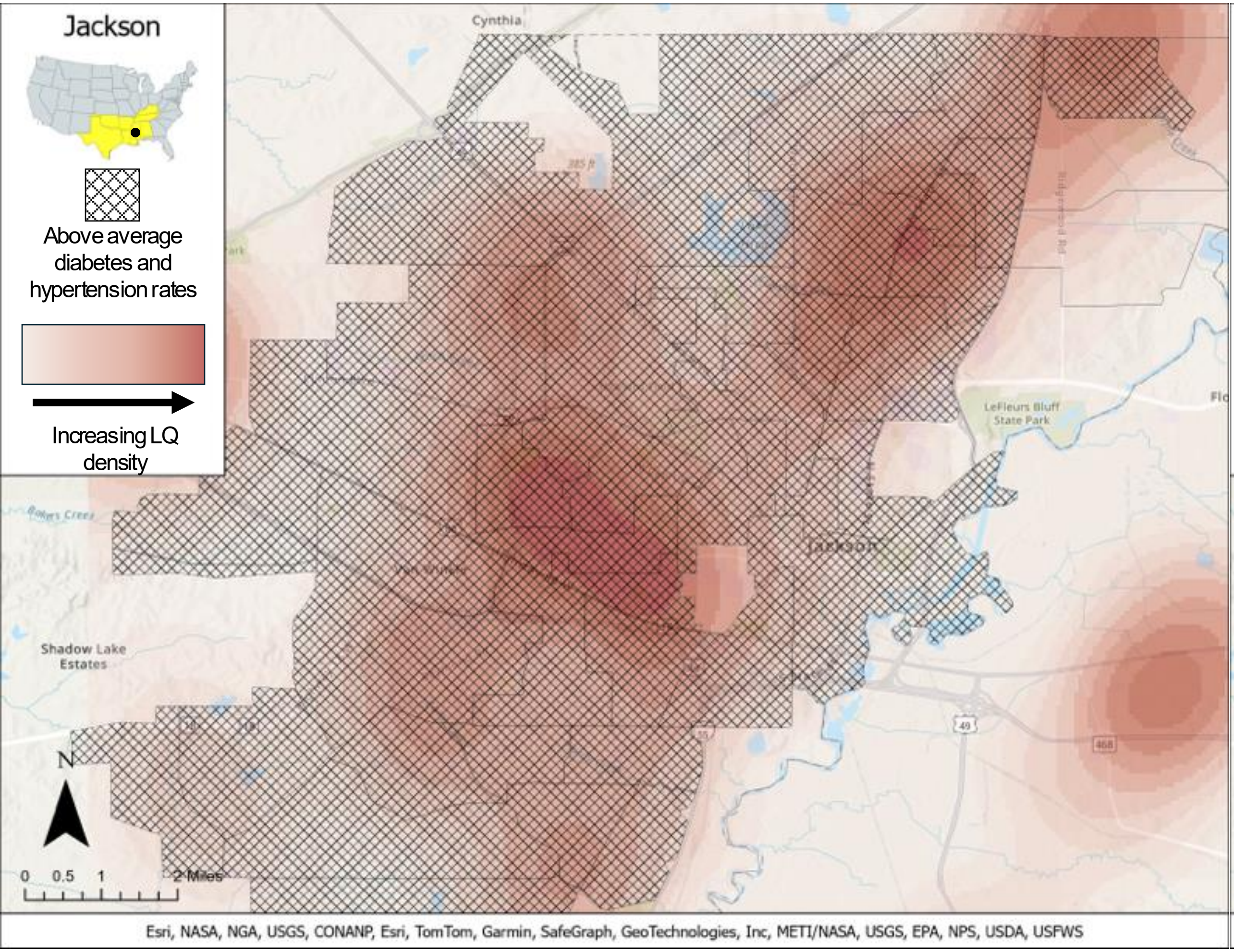

B)

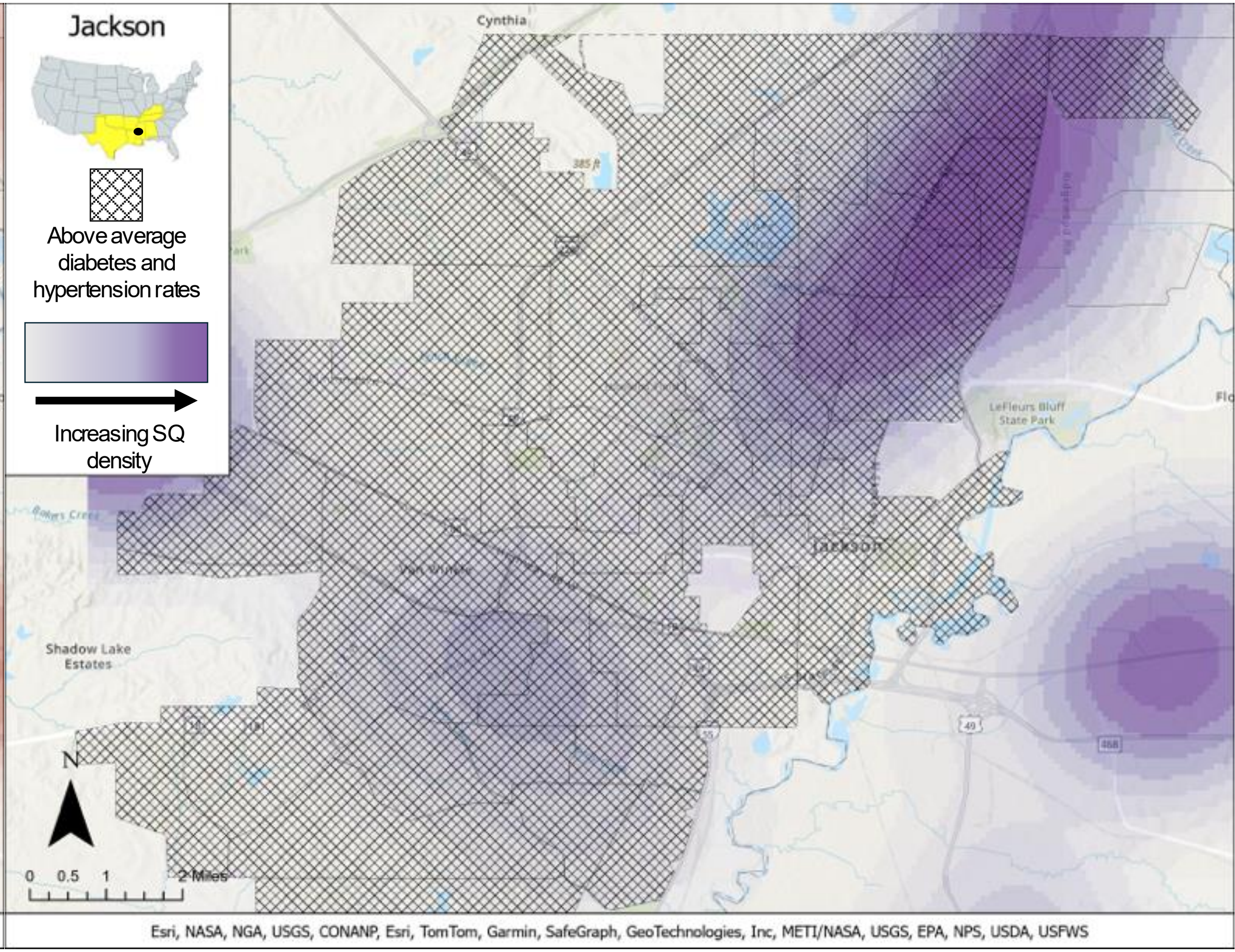

Side by side kernel analysis overlay with hatched tracts for the city of Jackson, Mississippi. Hatched tracts represent above US average diabetes and hypertension rates. A) Red kernel shaded areas are LQ (dollar store) dense areas. B) Purple kernel shaded areas are SQ (traditional grocery store) dense areas. LQ = Low Quality, SQ = Standard Quality

Kansas City, Missouri (Midwest Region)

A) B)

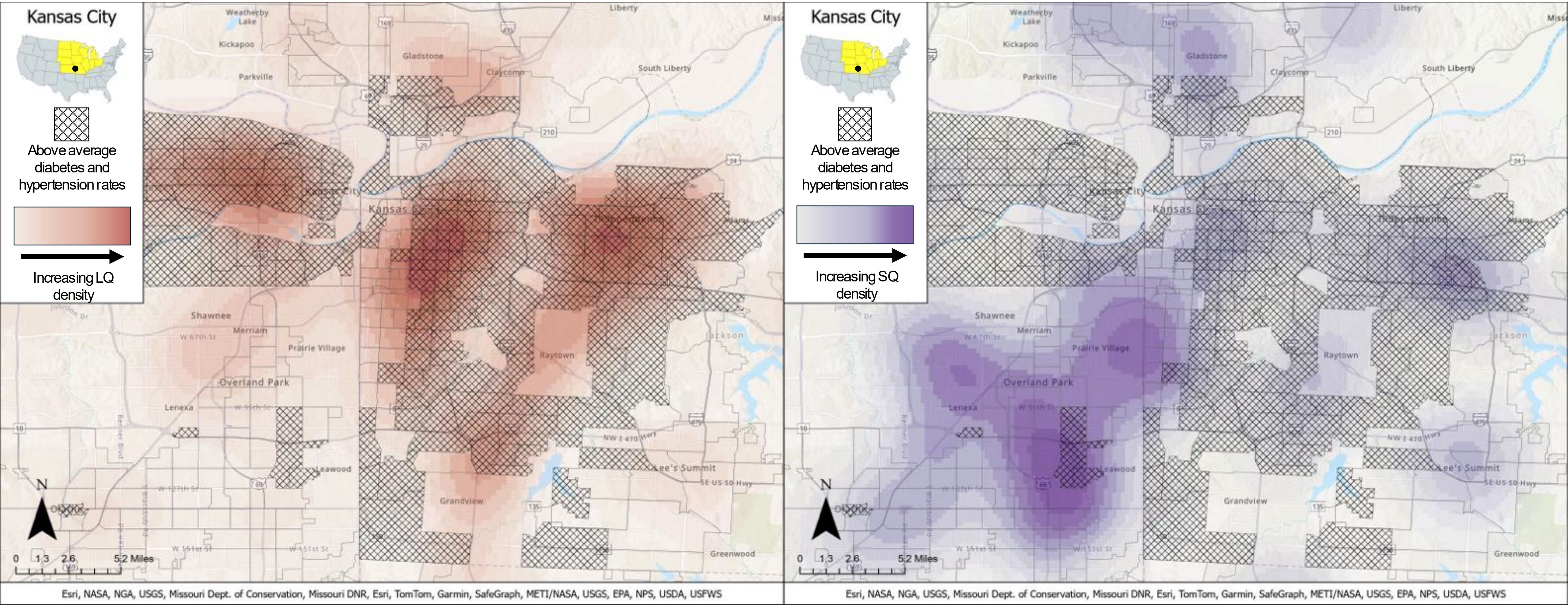

Side by side kernel analysis overlay with hatched tracts for the metro of Kansas City, Missouri. Hatched tracts represent above US average diabetes and hypertension rates. A) Red kernel shaded areas are LQ (dollar store) dense areas. B) Purple kernel shaded areas are SQ (traditional grocery store) dense areas. LQ = Low Quality, SQ = Standard Quality

Las Vegas, Nevada (Mountain Region)

A) B)

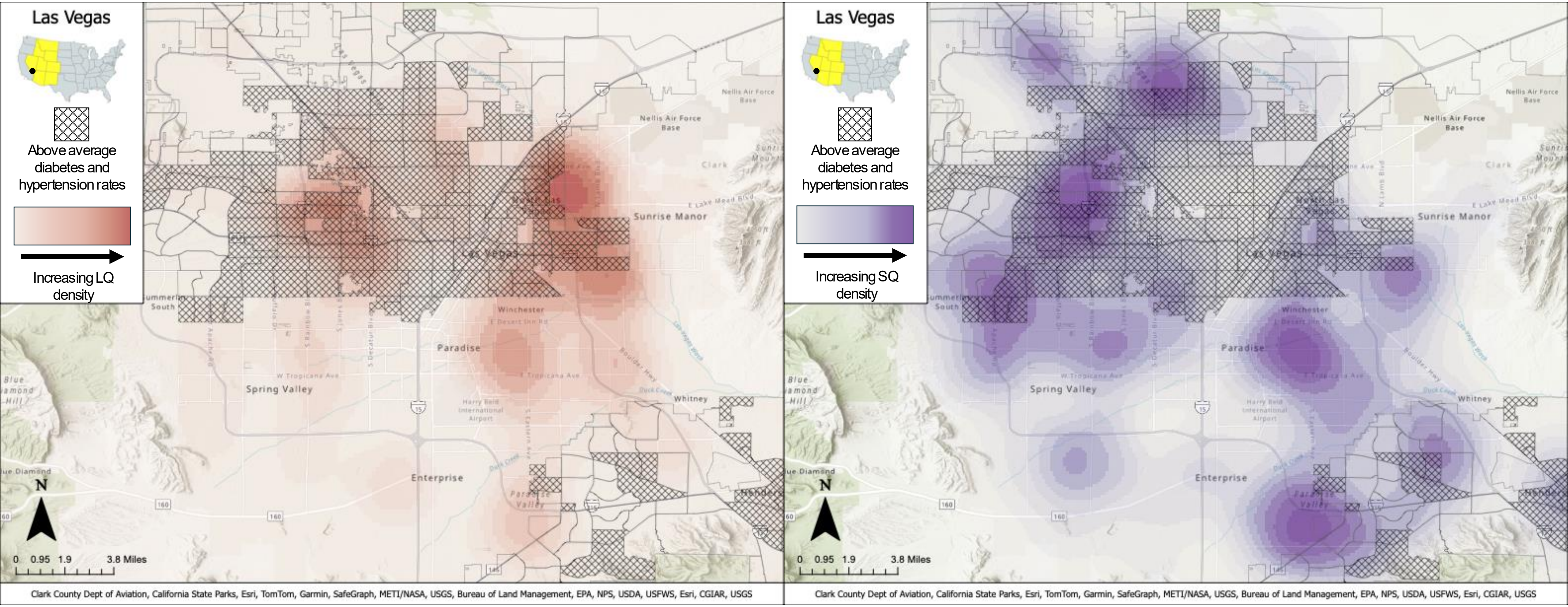

Side by side kernel analysis overlay with hatched tracts for the city of Las Vegas, Nevada. Hatched tracts represent above US average diabetes and hypertension rates. A) Red kernel shaded areas are LQ (dollar store) dense areas. B) Purple kernel shaded areas are SQ (traditional grocery store) dense areas. LQ = Low Quality, SQ = Standard Quality

Little Rock, Arkansas (South Region)

A) B)

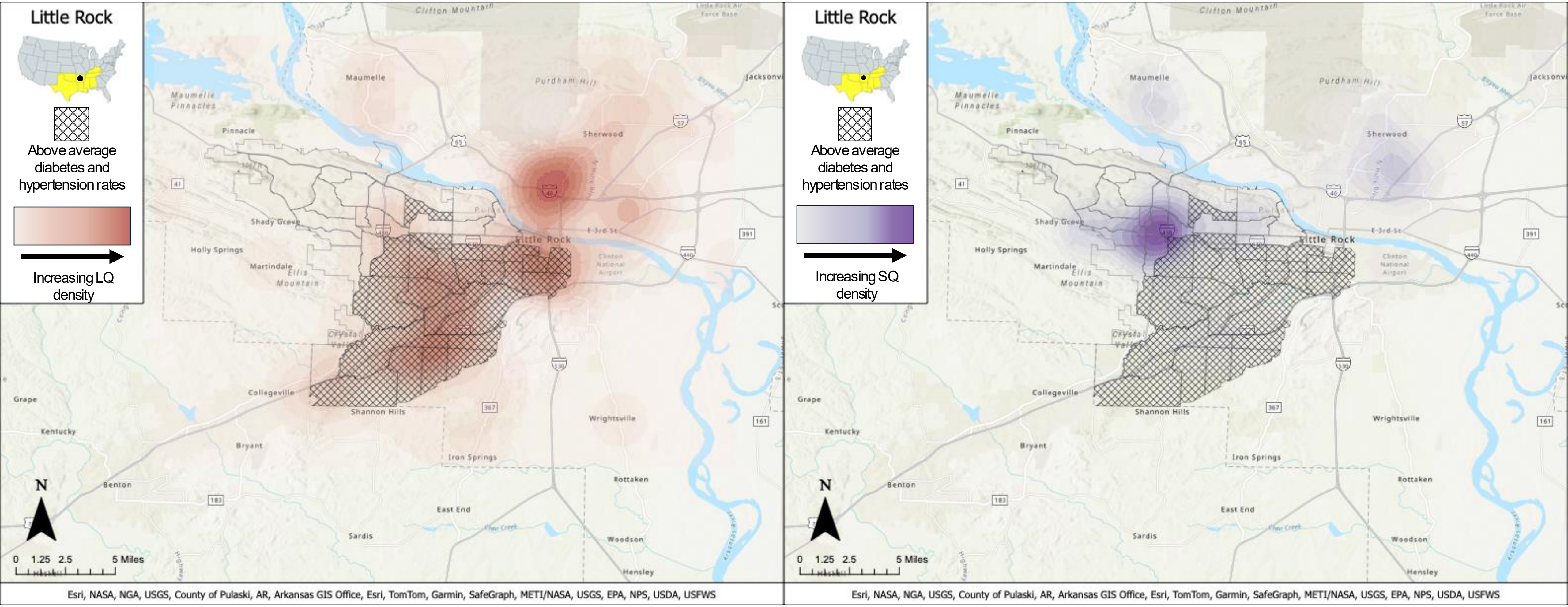

Side by side kernel analysis overlay with hatched tracts for the city of Little Rock, Arkansas. Hatched tracts represent above US average diabetes and hypertension rates. A) Red kernel shaded areas are LQ (dollar store) dense areas. B) Purple kernel shaded areas are SQ (traditional grocery store) dense areas. LQ = Low Quality, SQ = Standard Quality

Long Island, New York (Northeast Region)

A) B)

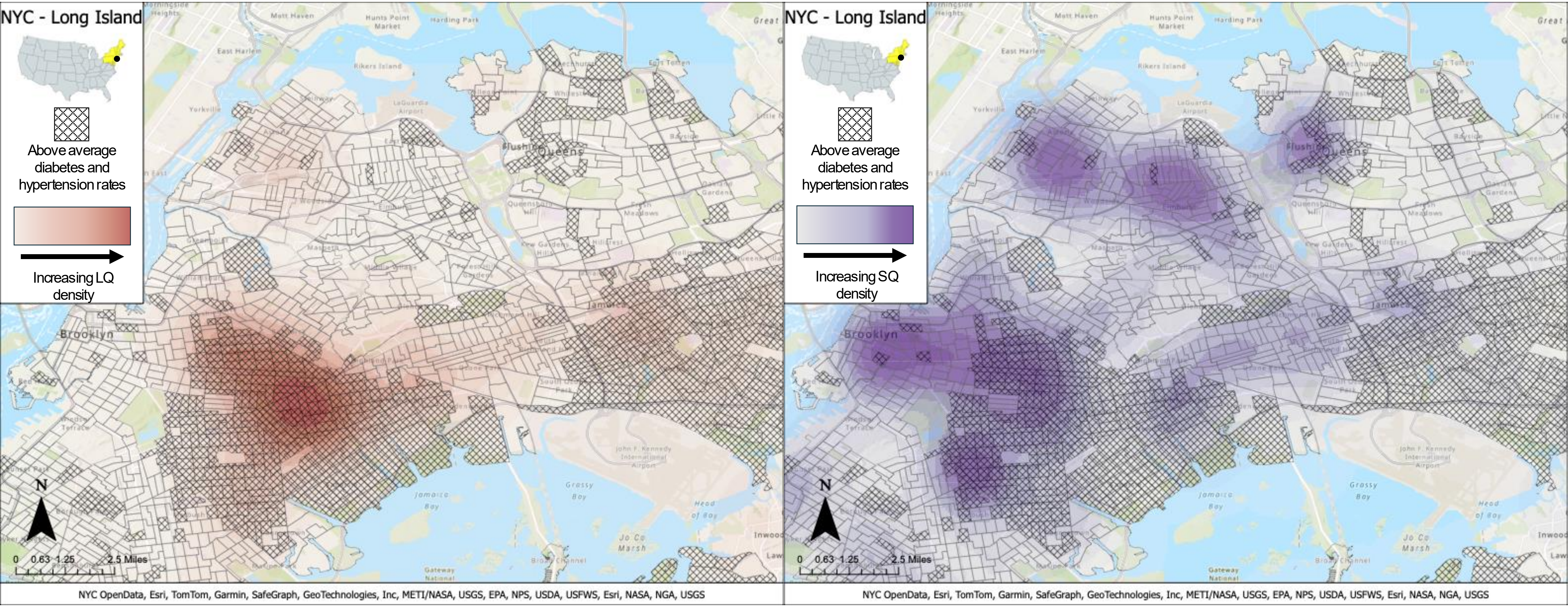

Side by side kernel analysis overlay with hatched tracts for New York City (Long Island), New York. Hatched tracts represent above US average diabetes and hypertension rates. A) Red kernel shaded areas are LQ (dollar store) dense areas. B) Purple kernel shaded areas are SQ (traditional grocery store) dense areas. LQ = Low Quality, SQ = Standard Quality

Los Angeles, California (Pacific Region)

A) B)

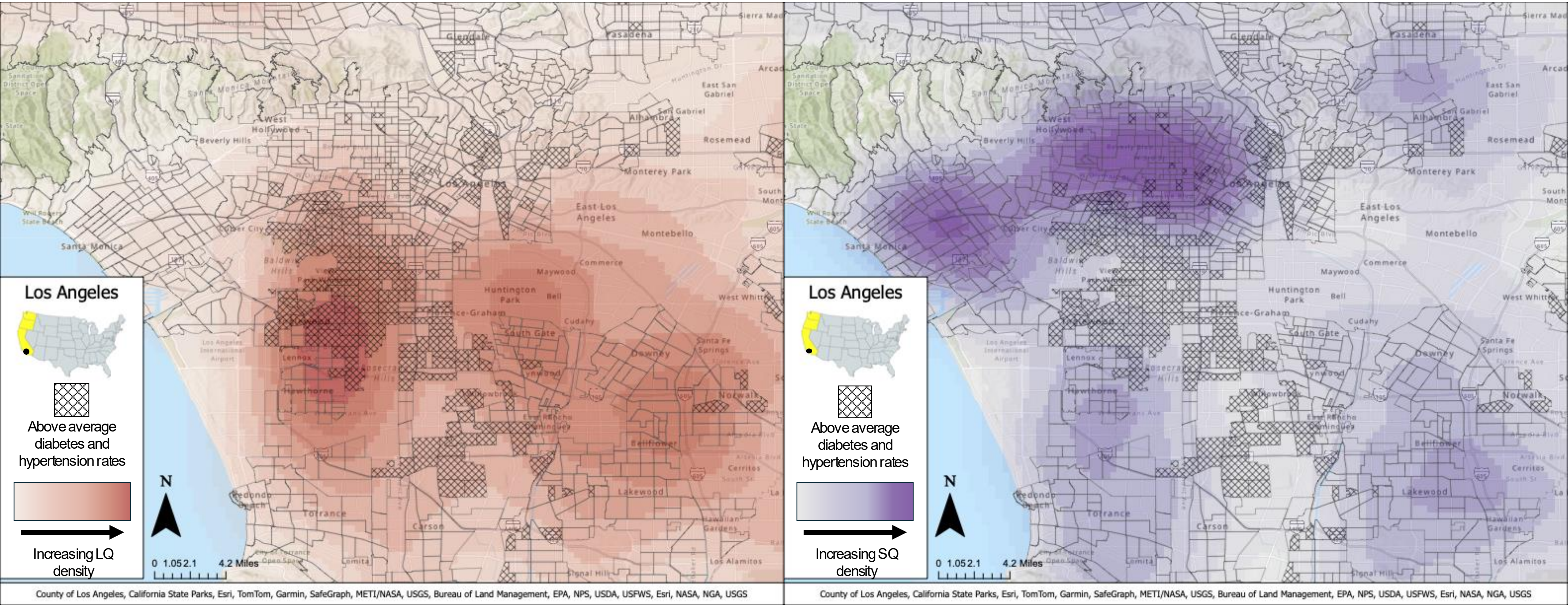

Side by side kernel analysis overlay with hatched tracts for the metro of Los Angeles, California. Hatched tracts represent above US average diabetes and hypertension rates. A) Red kernel shaded areas are LQ (dollar store) dense areas. B) Purple kernel shaded areas are SQ (traditional grocery store) dense areas. LQ = Low Quality, SQ = Standard Quality

Louisville, Kentucky (South Region)

A) B)

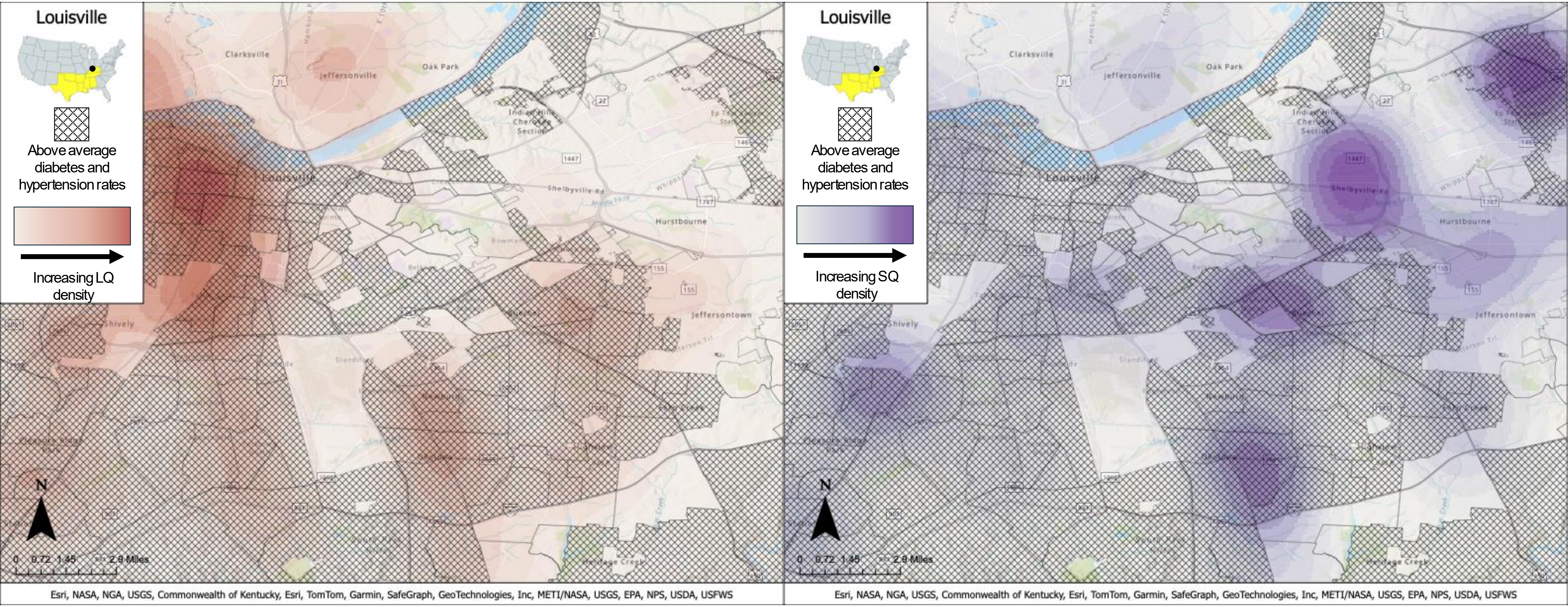

Side by side kernel analysis overlay with hatched tracts for the city of Louisville, Kentucky. Hatched tracts represent above US average diabetes and hypertension rates. A) Red kernel shaded areas are LQ (dollar store) dense areas. B) Purple kernel shaded areas are SQ (traditional grocery store) dense areas. LQ = Low Quality, SQ = Standard Quality

Manhattan, New York (Northeast Region)

A) B)

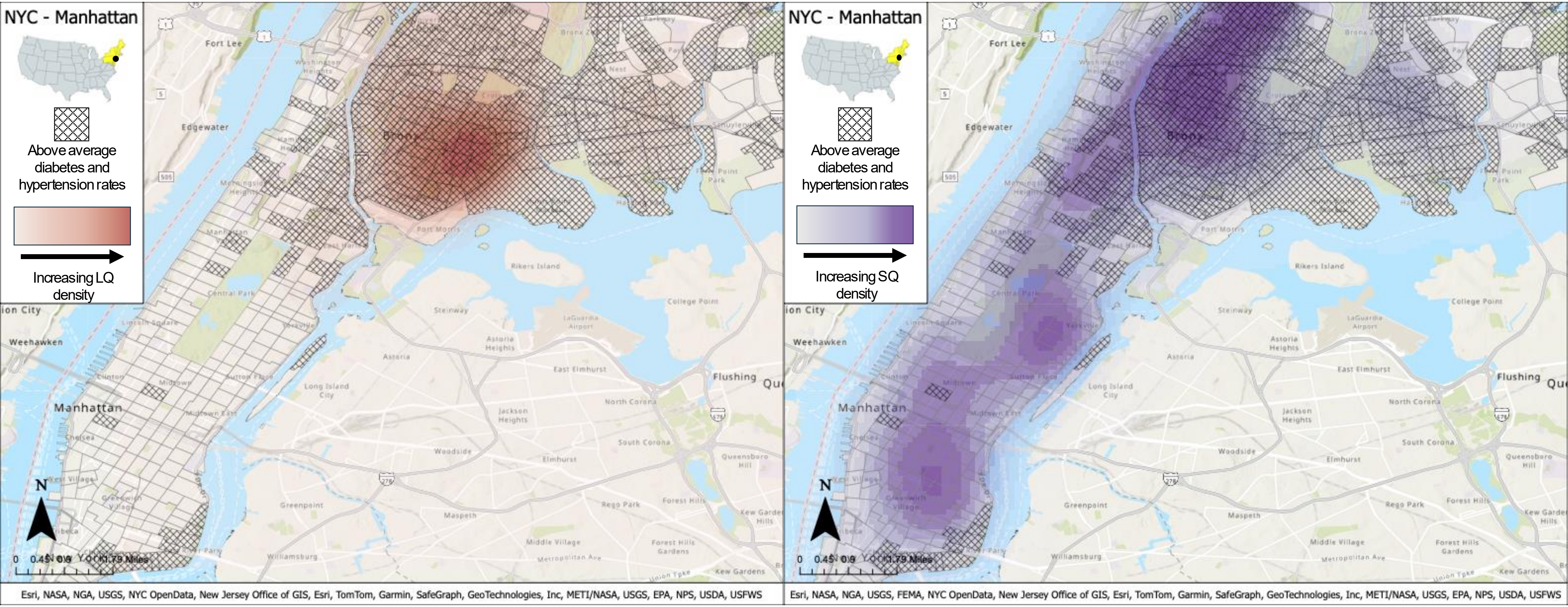

Side by side kernel analysis overlay with hatched tracts for New York City (Manhattan), New York. Hatched tracts represent above US average diabetes and hypertension rates. A) Red kernel shaded areas are LQ (dollar store) dense areas. B) Purple kernel shaded areas are SQ (traditional grocery store) dense areas. LQ = Low Quality, SQ = Standard Quality

Memphis, Tennessee (South Region)

A) B)

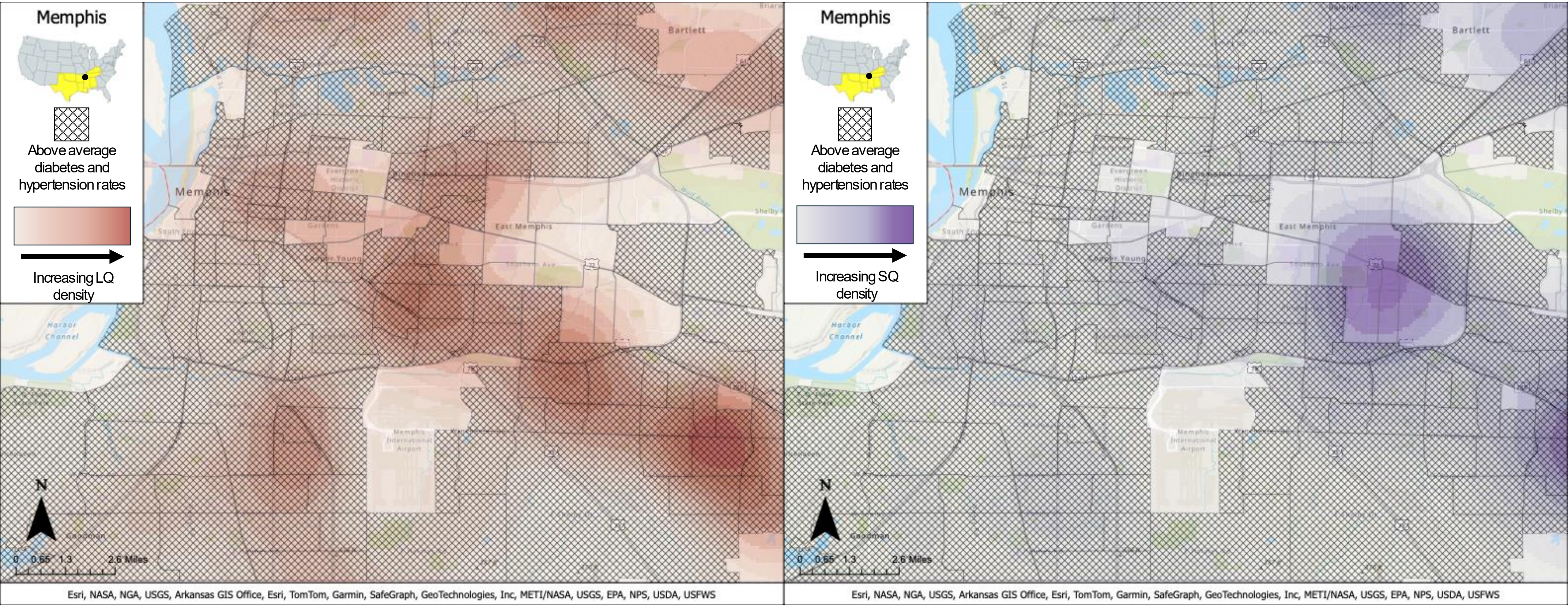

Side by side kernel analysis overlay with hatched tracts for the city of Memphis, Tennessee. Hatched tracts represent above US average diabetes and hypertension rates. A) Red kernel shaded areas are LQ (dollar store) dense areas. B) Purple kernel shaded areas are SQ (traditional grocery store) dense areas. LQ = Low Quality, SQ = Standard Quality

Miami, Florida (South Atlantic Region)

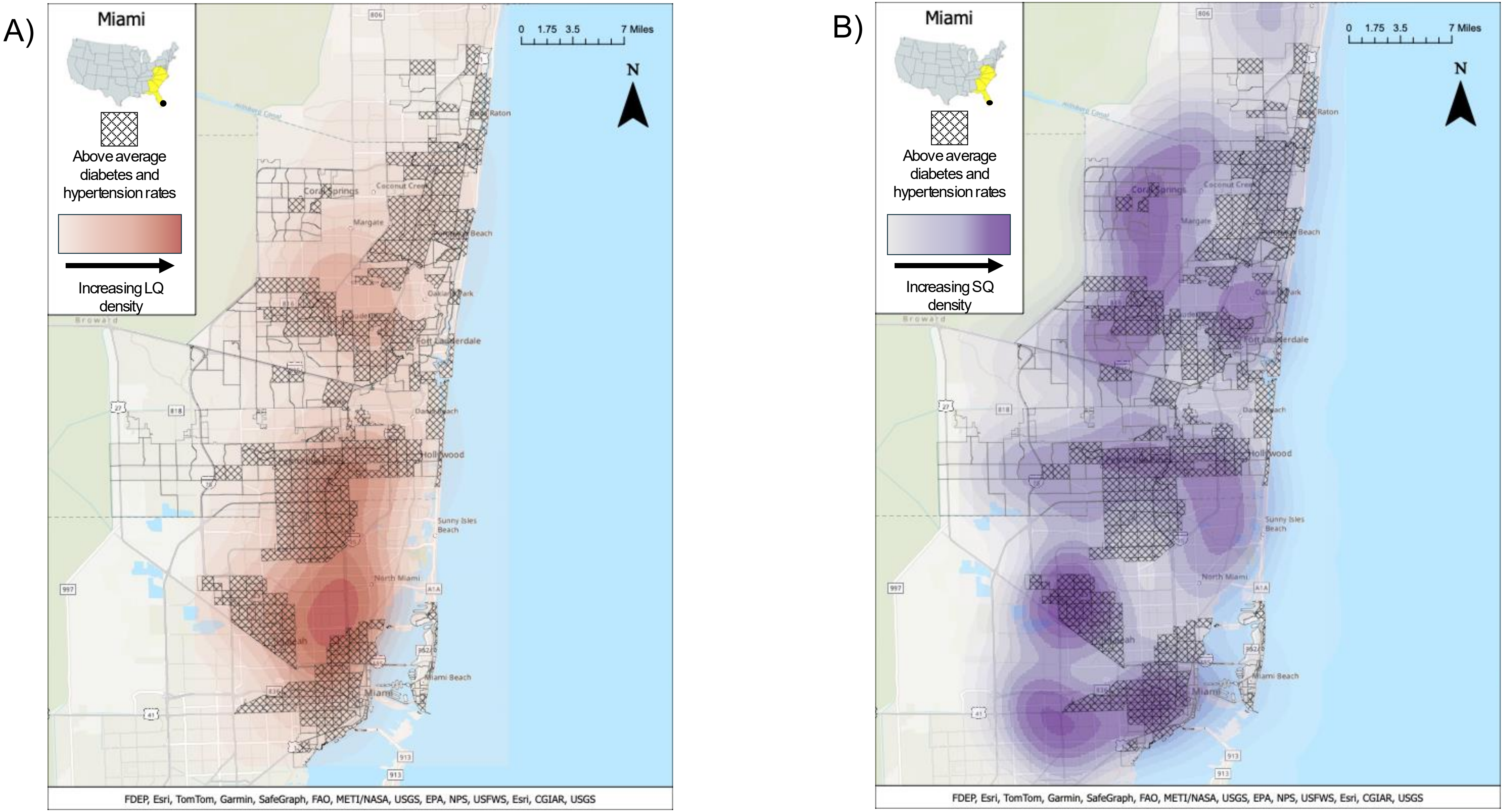

Side by side kernel analysis overlay with hatched tracts for the metro of Miami / Fort Lauderdale, Florida. Hatched tracts represent above US average diabetes and hypertension rates. A) Red kernel shaded areas are LQ (dollar store) dense areas. B) Purple kernel shaded areas are SQ (traditional grocery store) dense areas. LQ = Low Quality, SQ = Standard Quality

Milwaukee, Wisconsin (Midwest Region)

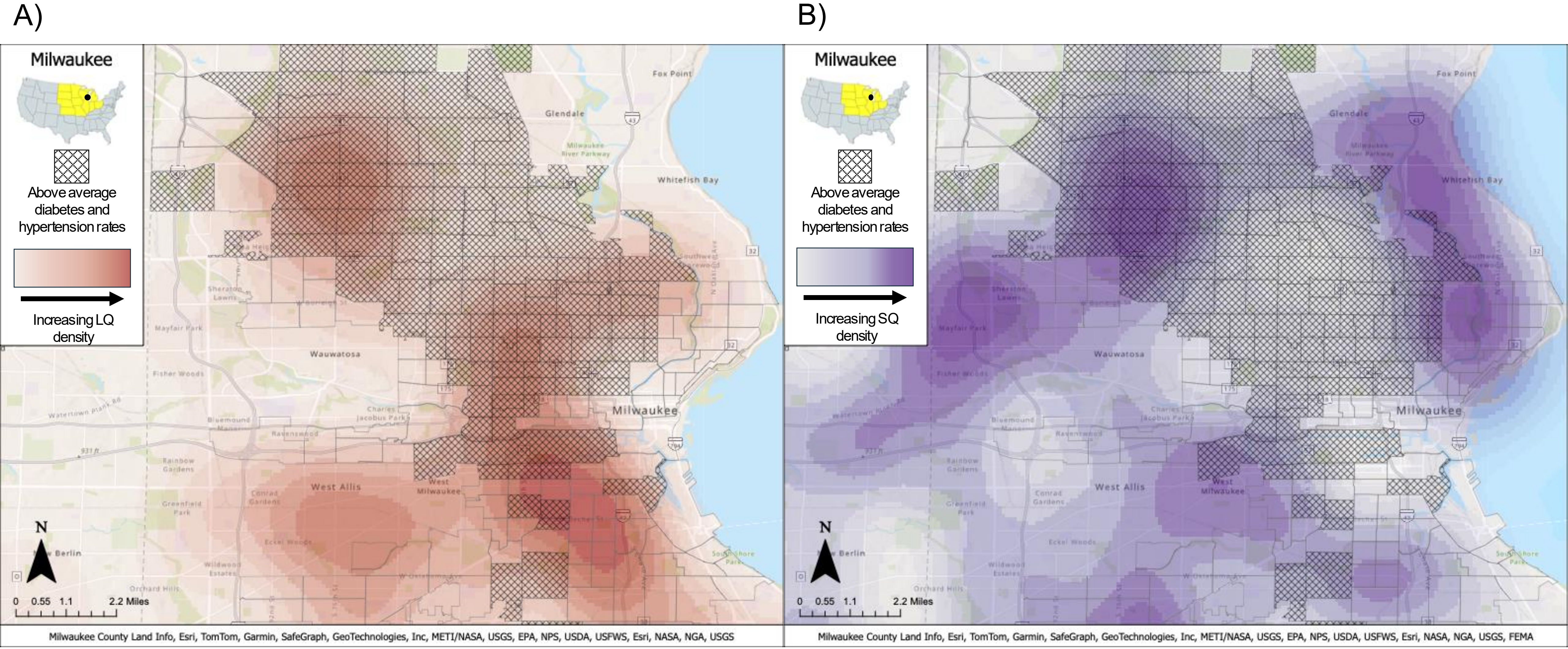

Side by side kernel analysis overlay with hatched tracts for the city of Milwaukee, Wisconsin. Hatched tracts represent above US average diabetes and hypertension rates. A) Red kernel shaded areas are LQ (dollar store) dense areas. B) Purple kernel shaded areas are SQ (traditional grocery store) dense areas. LQ = Low Quality, SQ = Standard Quality

Minneapolis, Minnesota (Midwest Region)

A) B)

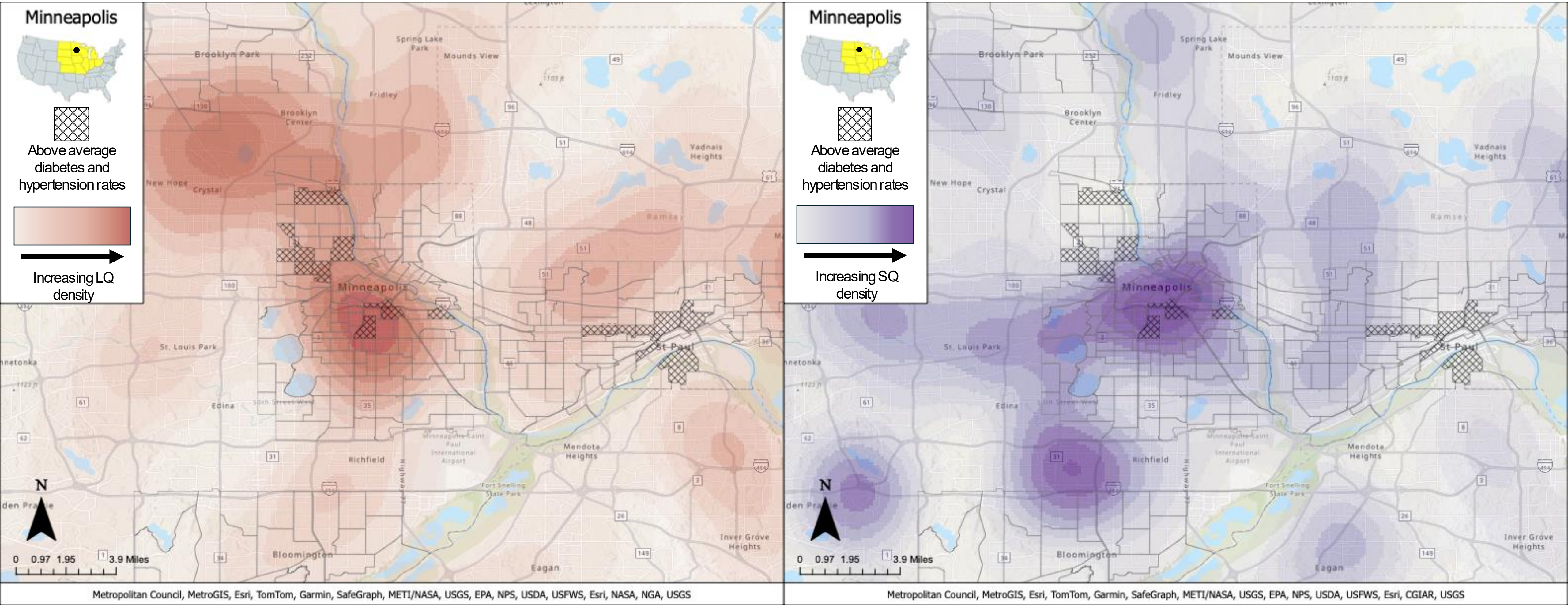

Side by side kernel analysis overlay with hatched tracts for the metro of Minneapolis, Minnesota. Hatched tracts represent above US average diabetes and hypertension rates. A) Red kernel shaded areas are LQ (dollar store) dense areas. B) Purple kernel shaded areas are SQ (traditional grocery store) dense areas. LQ = Low Quality, SQ = Standard Quality

Nashville, Tennessee (South Region)

A) B)

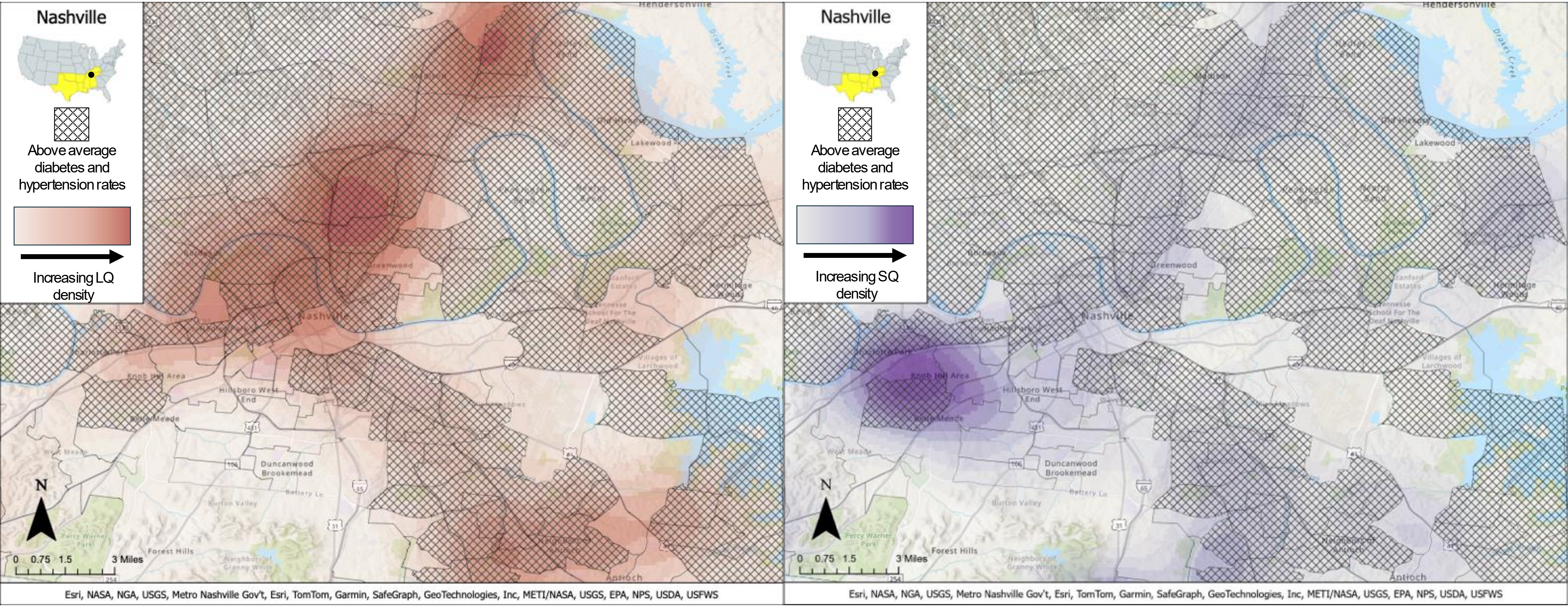

Side by side kernel analysis overlay with hatched tracts for the city of Nashville, Tennessee. Hatched tracts represent above US average diabetes and hypertension rates. A) Red kernel shaded areas are LQ (dollar store) dense areas. B) Purple kernel shaded areas are SQ (traditional grocery store) dense areas. LQ = Low Quality, SQ = Standard Quality

New Jersey (Northeast Region)

A) B)

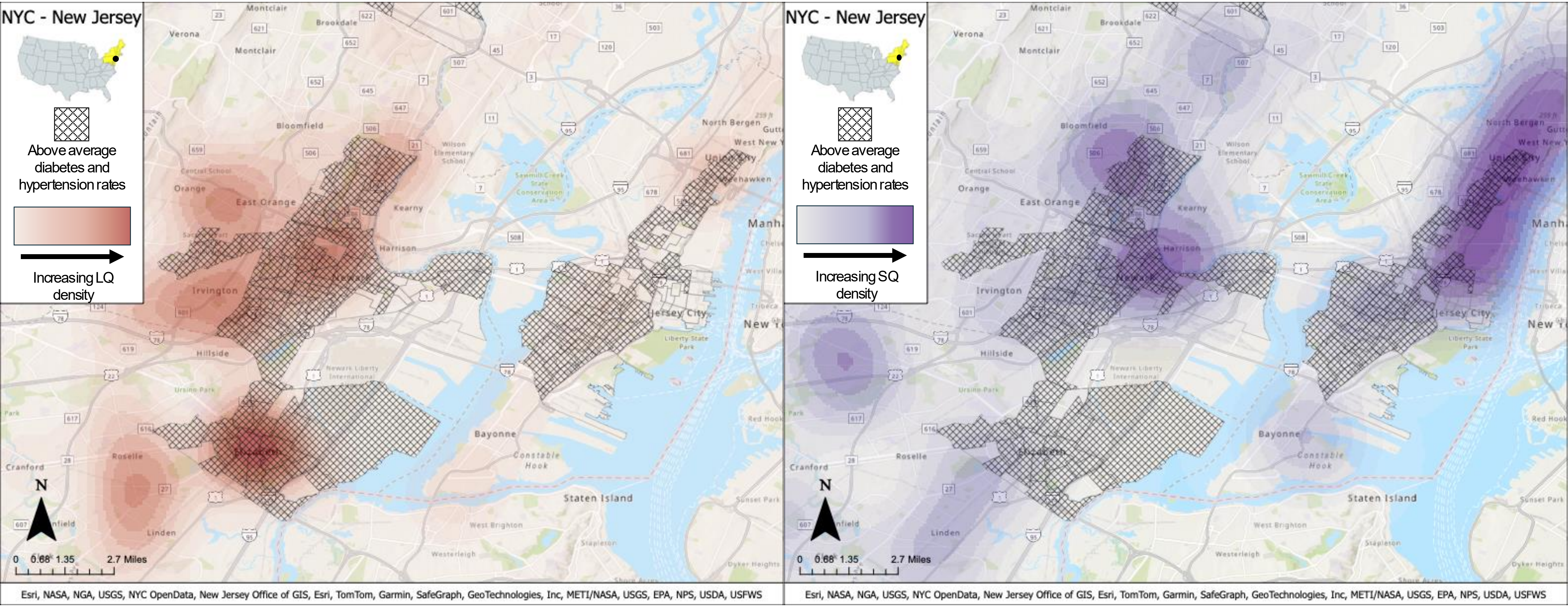

Side by side kernel analysis overlay with hatched tracts for the metro of New York City, New Jersey (Newark, Hoboken, Jersey City). Hatched tracts represent above US average diabetes and hypertension rates. A) Red kernel shaded areas are LQ (dollar store) dense areas. B) Purple kernel shaded areas are SQ (traditional grocery store) dense areas. LQ = Low Quality, SQ = Standard Quality

New Orleans, Louisiana (South Region)

A) B)

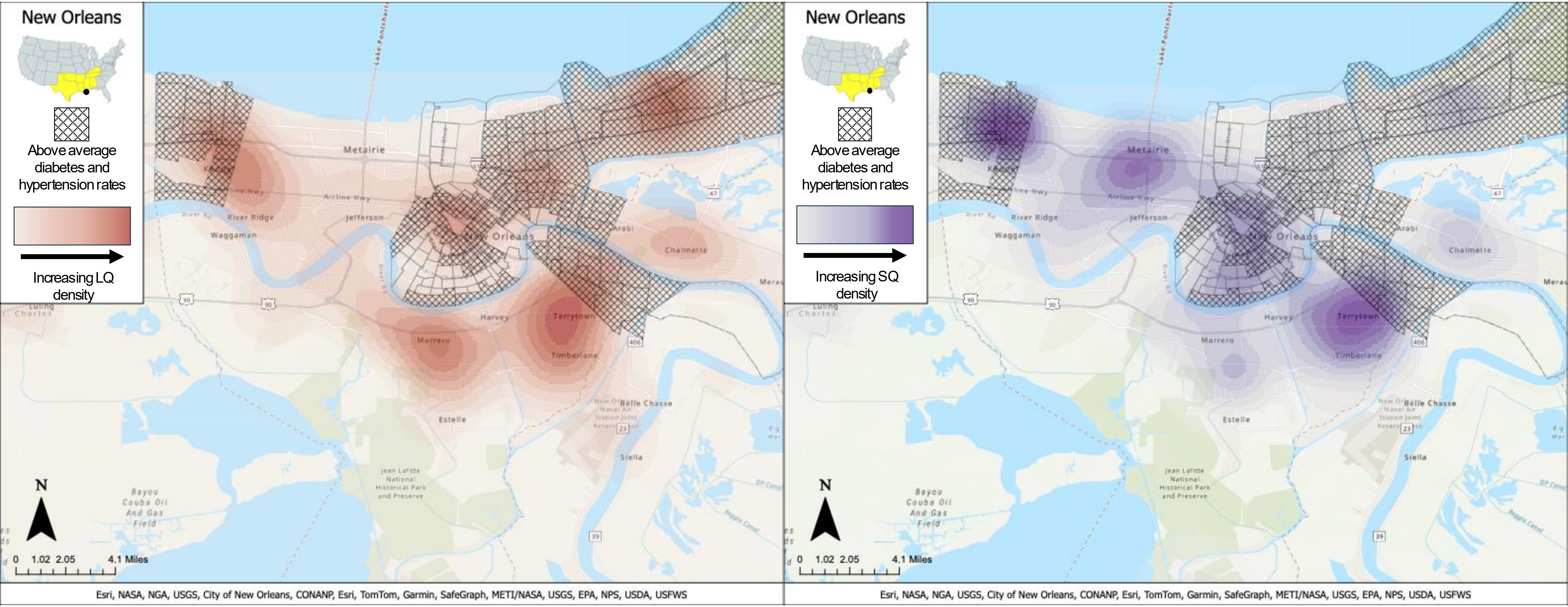

Side by side kernel analysis overlay with hatched tracts for the city of New Orleans, Louisiana. Hatched tracts represent above US average diabetes and hypertension rates. A) Red kernel shaded areas are LQ (dollar store) dense areas. B) Purple kernel shaded areas are SQ (traditional grocery store) dense areas. LQ = Low Quality, SQ = Standard Quality

Oklahoma City, Oklahoma (South Region)

A)

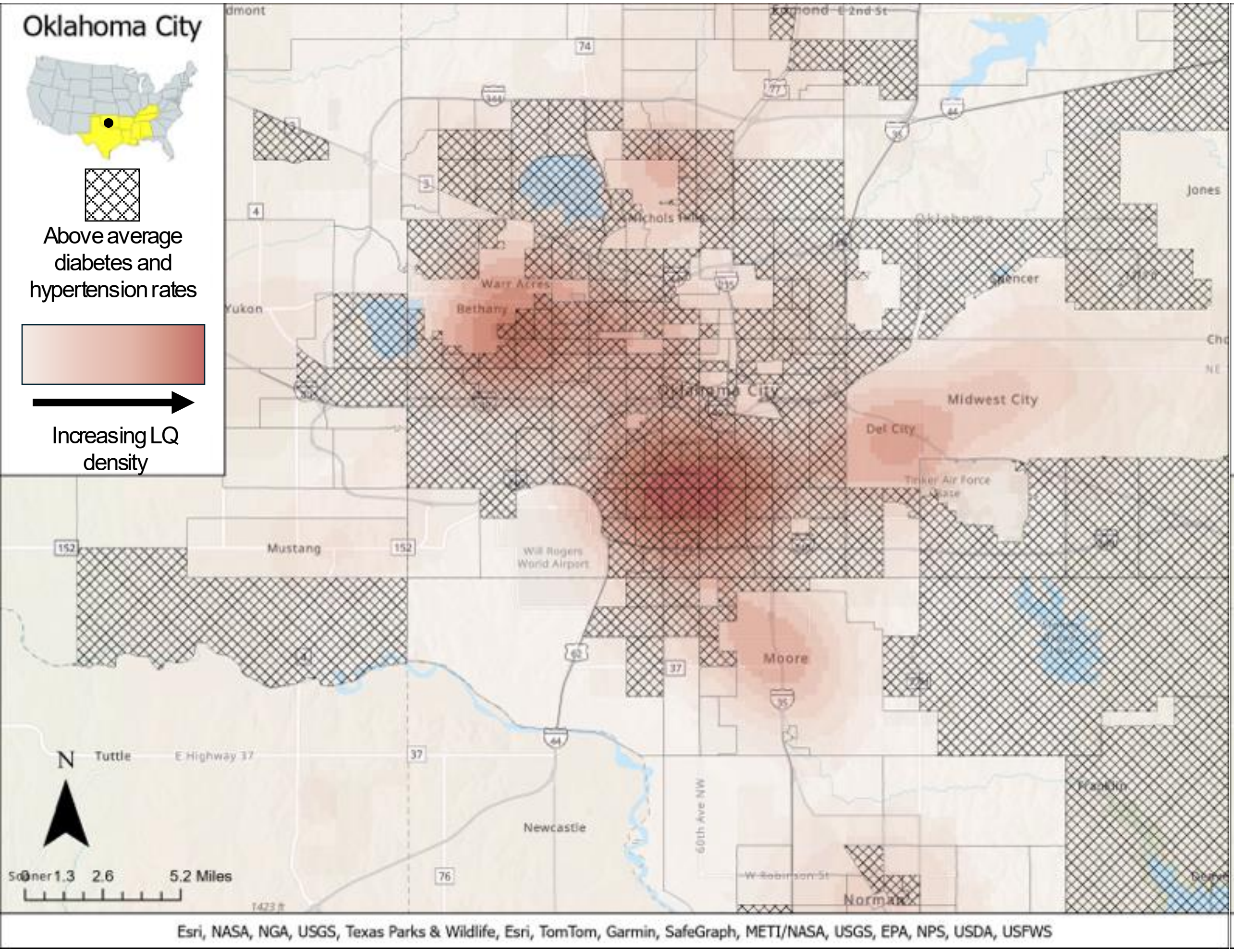

B)

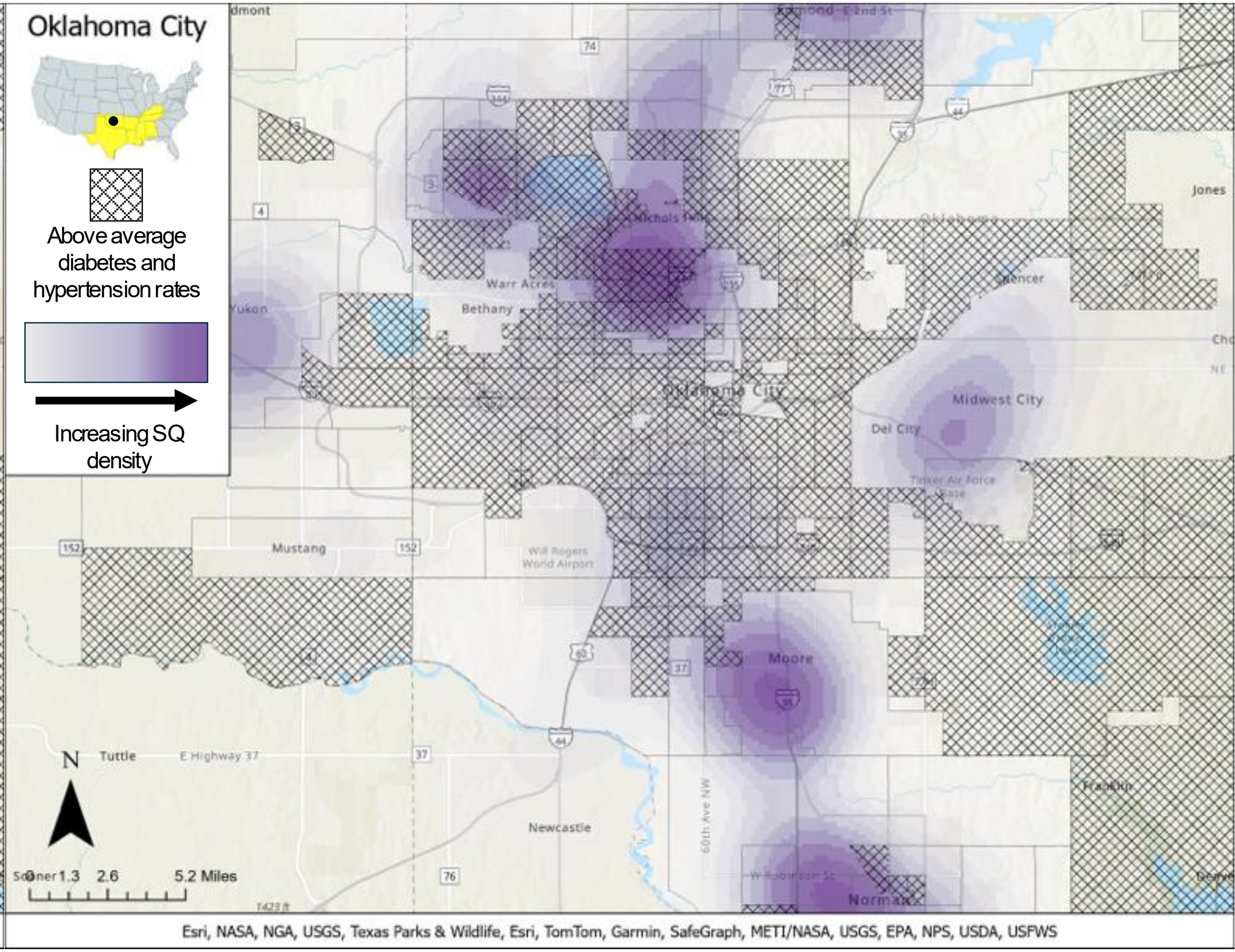

Side by side kernel analysis overlay with hatched tracts for the city of Oklahoma City, Oklahoma. Hatched tracts represent above US average diabetes and hypertension rates. A) Red kernel shaded areas are LQ (dollar store) dense areas. B) Purple kernel shaded areas are SQ (traditional grocery store) dense areas. LQ = Low Quality, SQ = Standard Quality

Omaha, Nebraska (Midwest Region)

A) B)

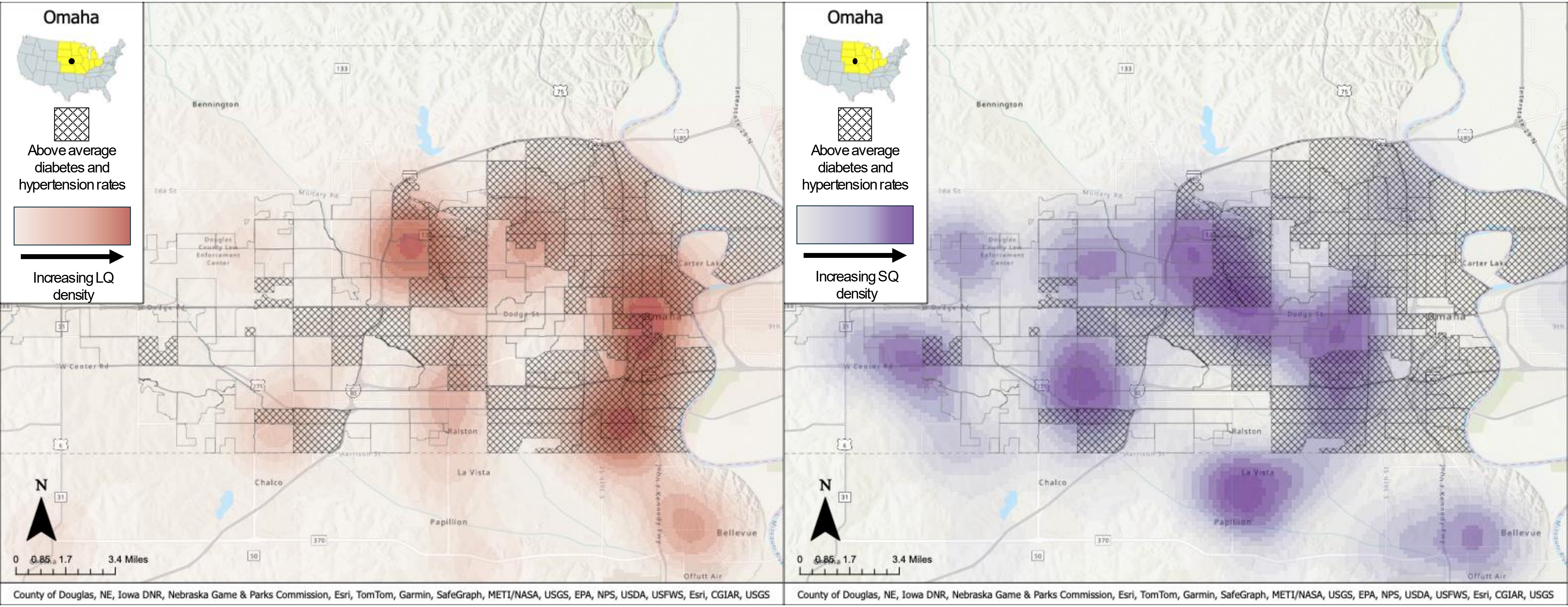

Side by side kernel analysis overlay with hatched tracts for the city of Omaha, Nebraska. Hatched tracts represent above US average diabetes and hypertension rates. A) Red kernel shaded areas are LQ (dollar store) dense areas. B) Purple kernel shaded areas are SQ (traditional grocery store) dense areas. LQ = Low Quality, SQ = Standard Quality

Orange County, California (Pacific Region)

A) B)

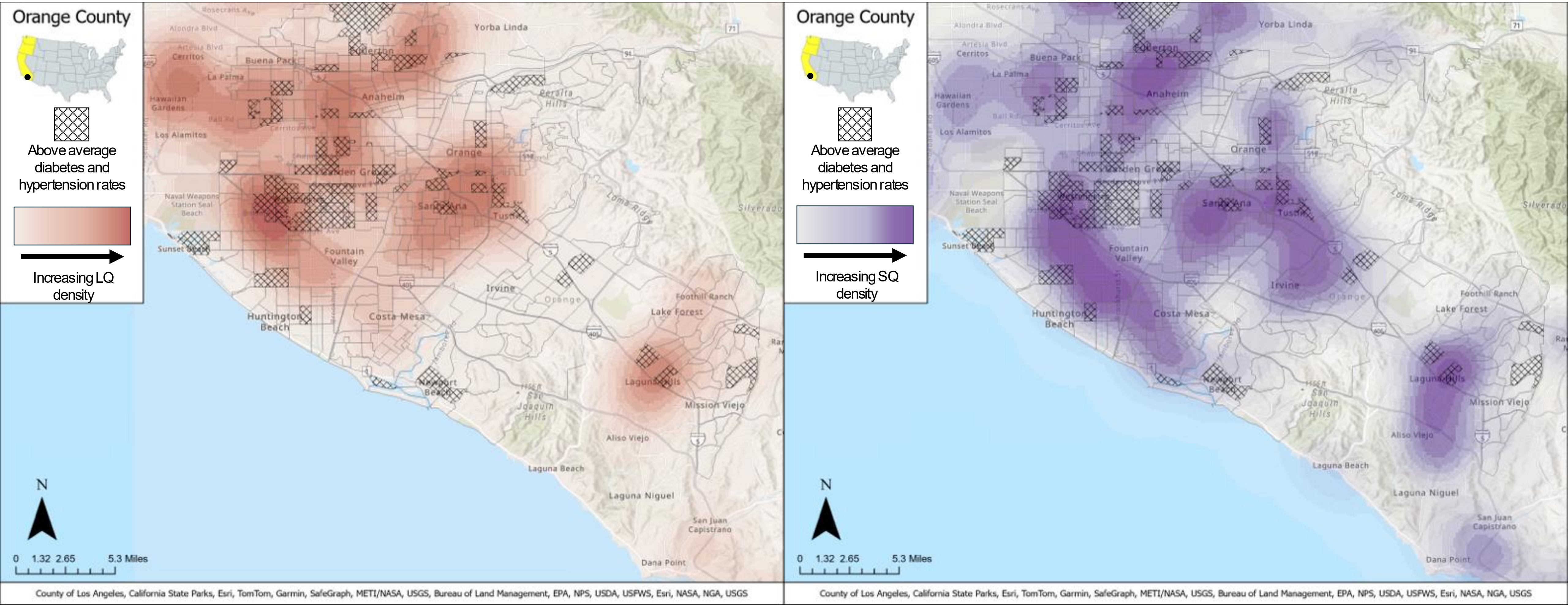

Side by side kernel analysis overlay with hatched tracts for the cities within Orange County, California. Hatched tracts represent above US average diabetes and hypertension rates. A) Red kernel shaded areas are LQ (dollar store) dense areas. B) Purple kernel shaded areas are SQ (traditional grocery store) dense areas. LQ = Low Quality, SQ = Standard Quality

Orlando, Florida (South Atlantic Region)

A) B)

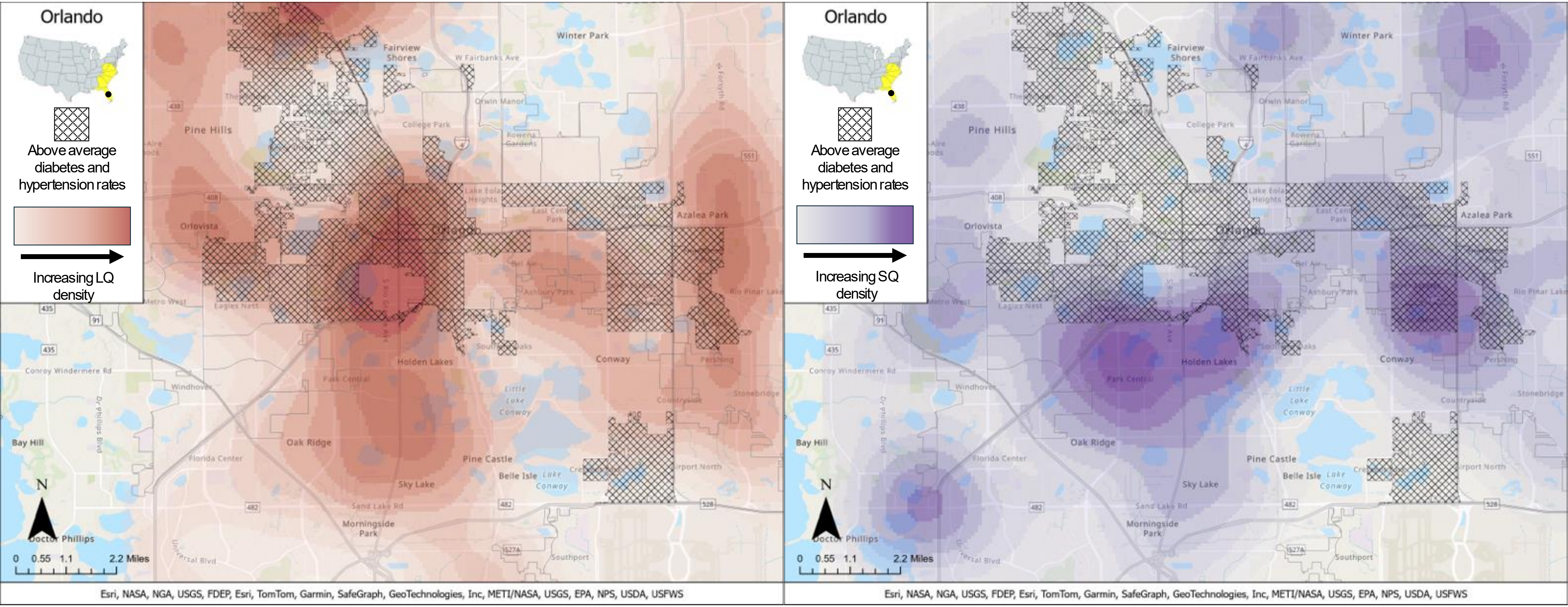

Side by side kernel analysis overlay with hatched tracts for the city of Orlando, Florida. Hatched tracts represent above US average diabetes and hypertension rates. A) Red kernel shaded areas are LQ (dollar store) dense areas. B) Purple kernel shaded areas are SQ (traditional grocery store) dense areas. LQ = Low Quality, SQ = Standard Quality

Philadelphia, Pennsylvania (Northeast Region)

A) B)

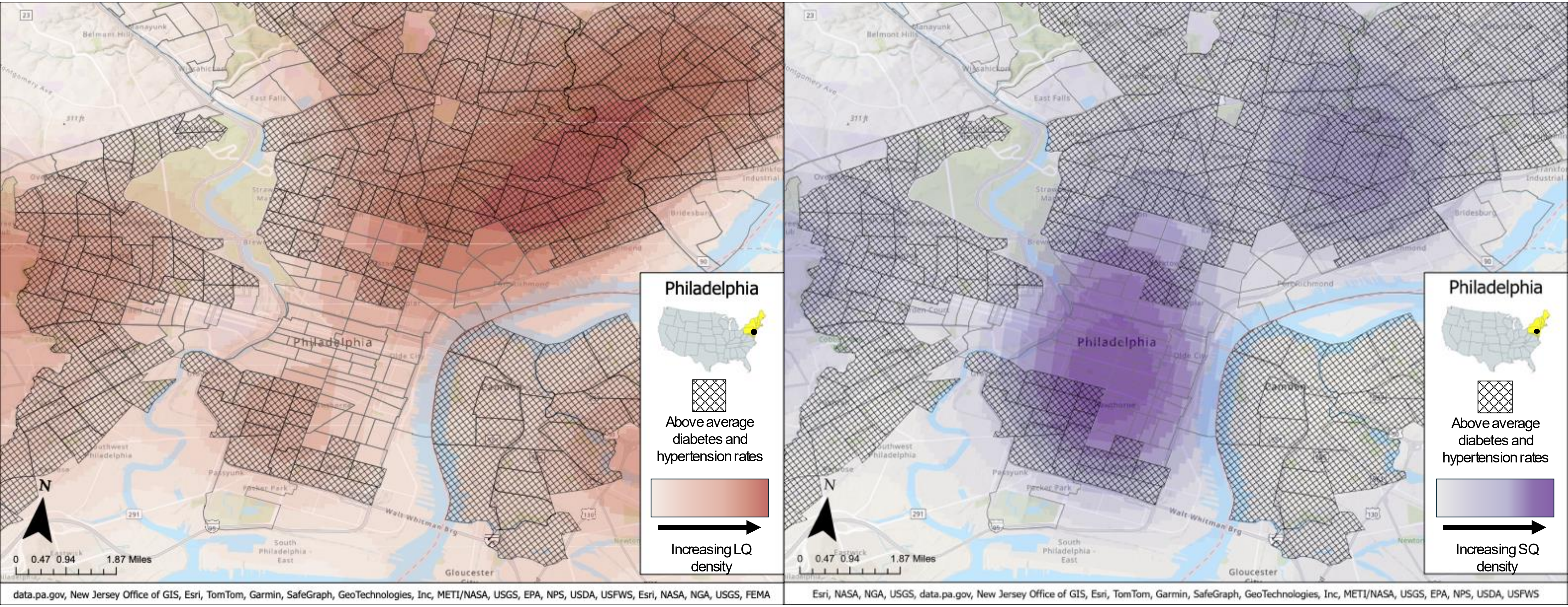

Side by side kernel analysis overlay with hatched tracts for the city of Philadelphia, Pennsylvania. Hatched tracts represent above US average diabetes and hypertension rates. A) Red kernel shaded areas are LQ (dollar store) dense areas. B) Purple kernel shaded areas are SQ (traditional grocery store) dense areas. LQ = Low Quality, SQ = Standard Quality

Pittsburgh, Pennsylvania (Northeast Region)

A)

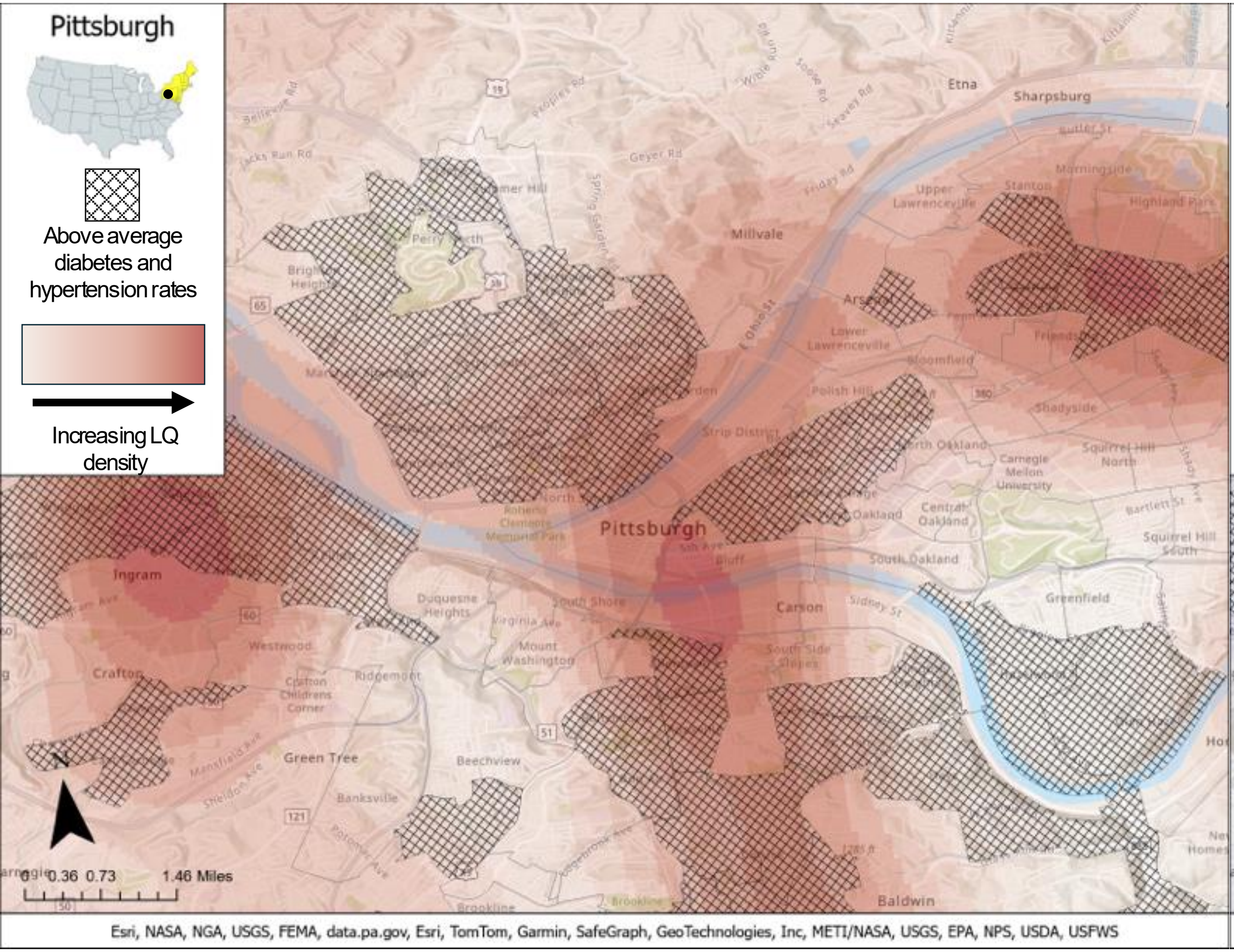

B)

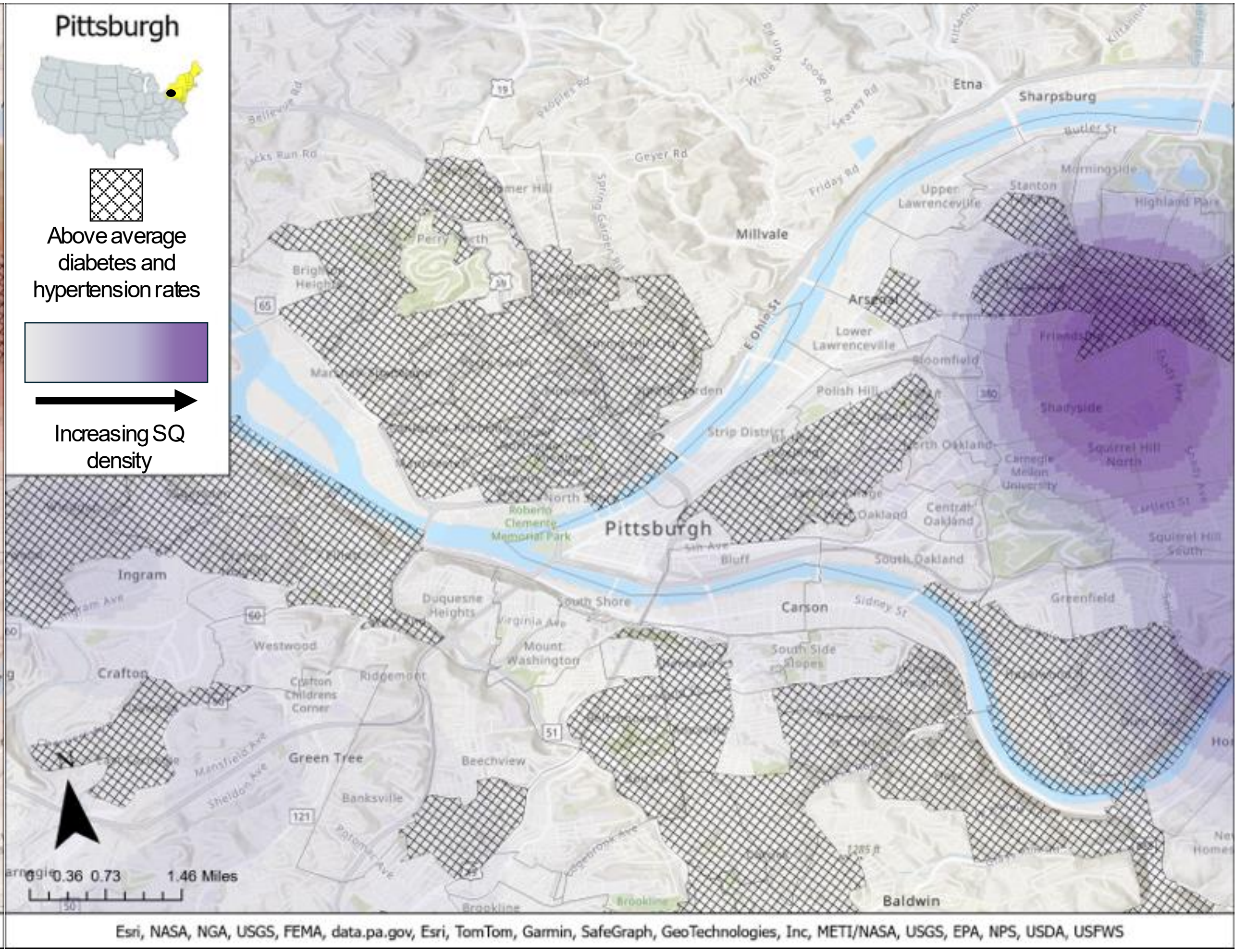

Side by side kernel analysis overlay with hatched tracts for the city of Pittsburgh, Pennsylvania. Hatched tracts represent above US average diabetes and hypertension rates. A) Red kernel shaded areas are LQ (dollar store) dense areas. B) Purple kernel shaded areas are SQ (traditional grocery store) dense areas. LQ = Low Quality, SQ = Standard Quality

Portland, Oregon (Pacific Region)

A) B)

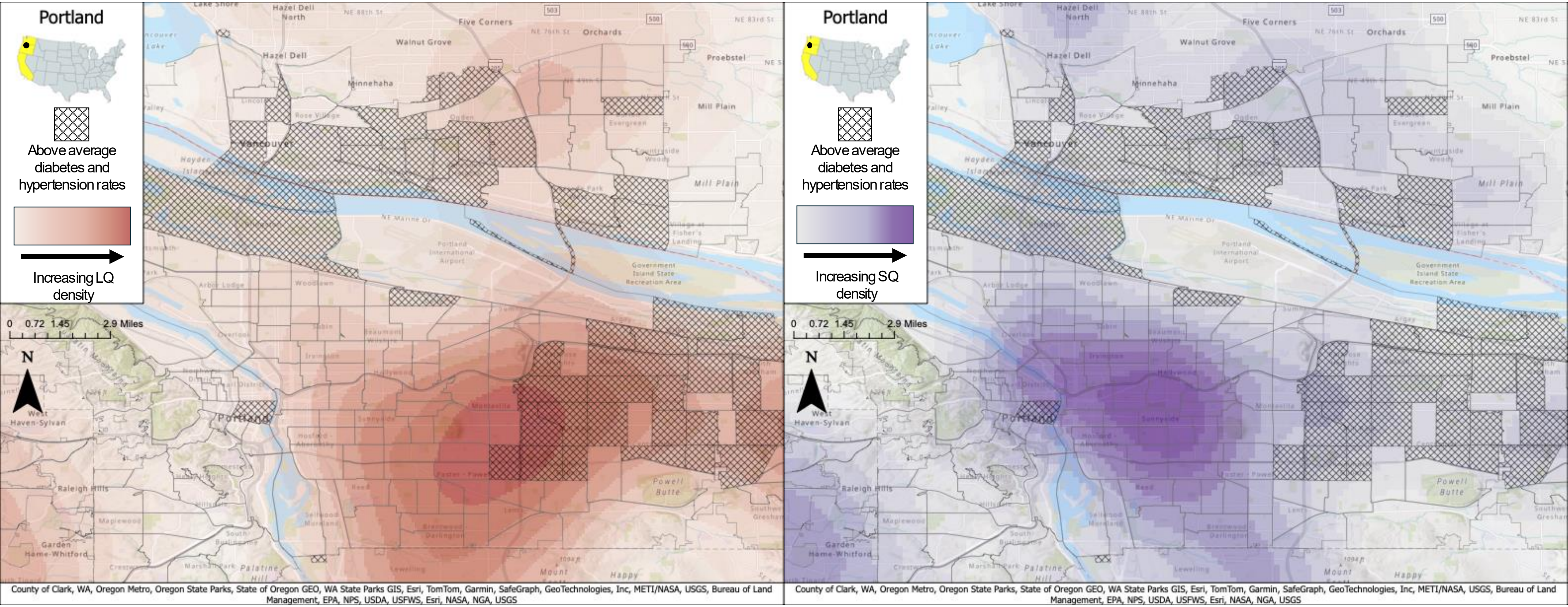

Side by side kernel analysis overlay with hatched tracts for the city of Portland, Oregon. Hatched tracts represent above US average diabetes and hypertension rates. A) Red kernel shaded areas are LQ (dollar store) dense areas. B) Purple kernel shaded areas are SQ (traditional grocery store) den se areas. LQ = Low Quality, SQ = Standard Quality

Providence, Rhode Island (Northeast Region)

A) B)

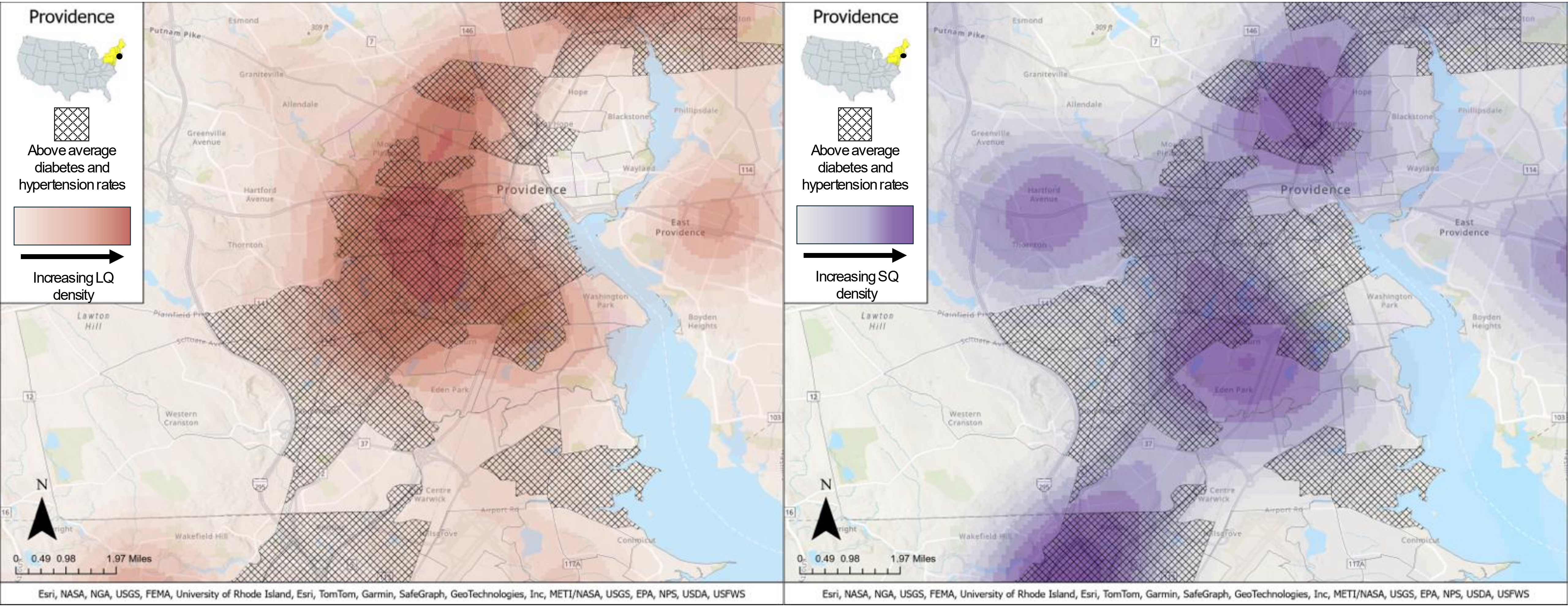

Side by side kernel analysis overlay with hatched tracts for the city of Providence, Rhode Island. Hatched tracts represent above US average diabetes and hypertension rates. A) Red kernel shaded areas are LQ (dollar store) dense areas. B) Purple kernel shaded areas are SQ (traditional grocery store) dense areas. LQ = Low Quality, SQ = Standard Quality

Raleigh, North Carolina (South Atlantic Region)

A) B)

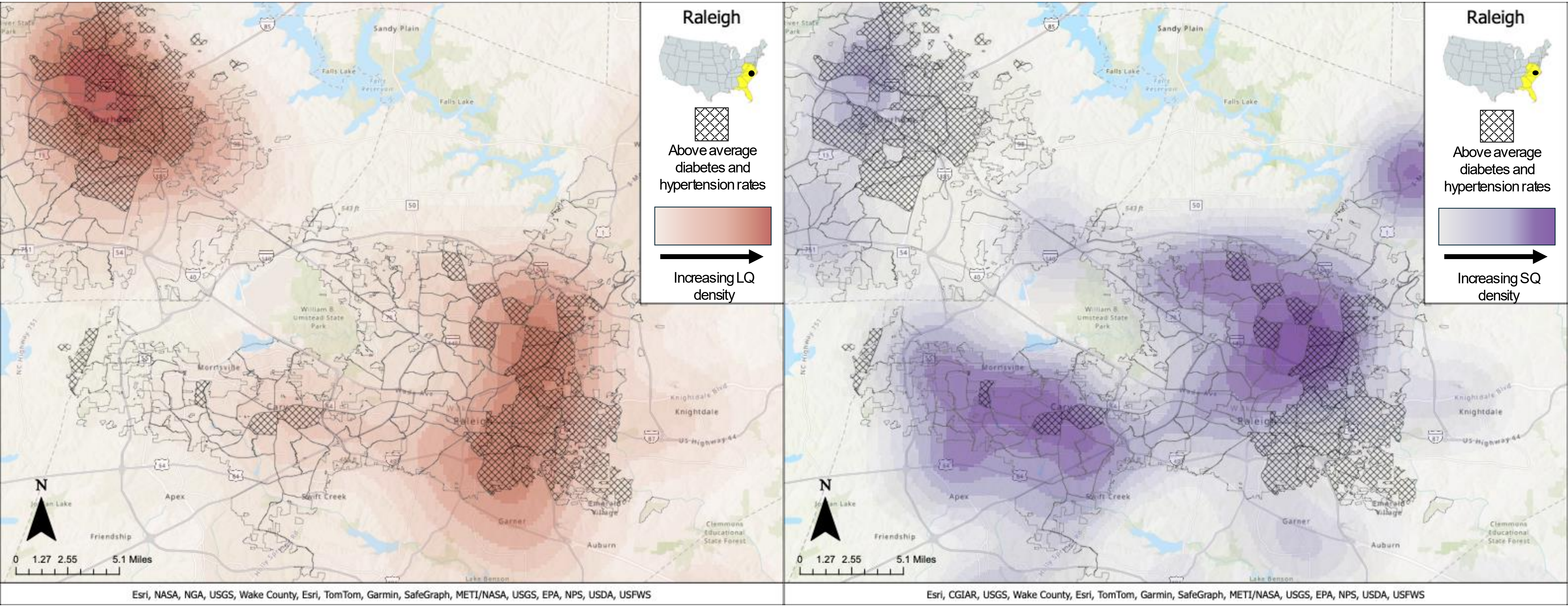

Side by side kernel analysis overlay with hatched tracts for the metro of Raleigh, North Carolina. Hatched tracts represent above US average diabetes and hypertension rates. A) Red kernel shaded areas are LQ (dollar store) dense areas. B) Purple kernel shaded areas are SQ (traditional grocery store) dense areas. LQ = Low Quality, SQ = Standard Quality

Sacramento, California (Pacific Region)

A)

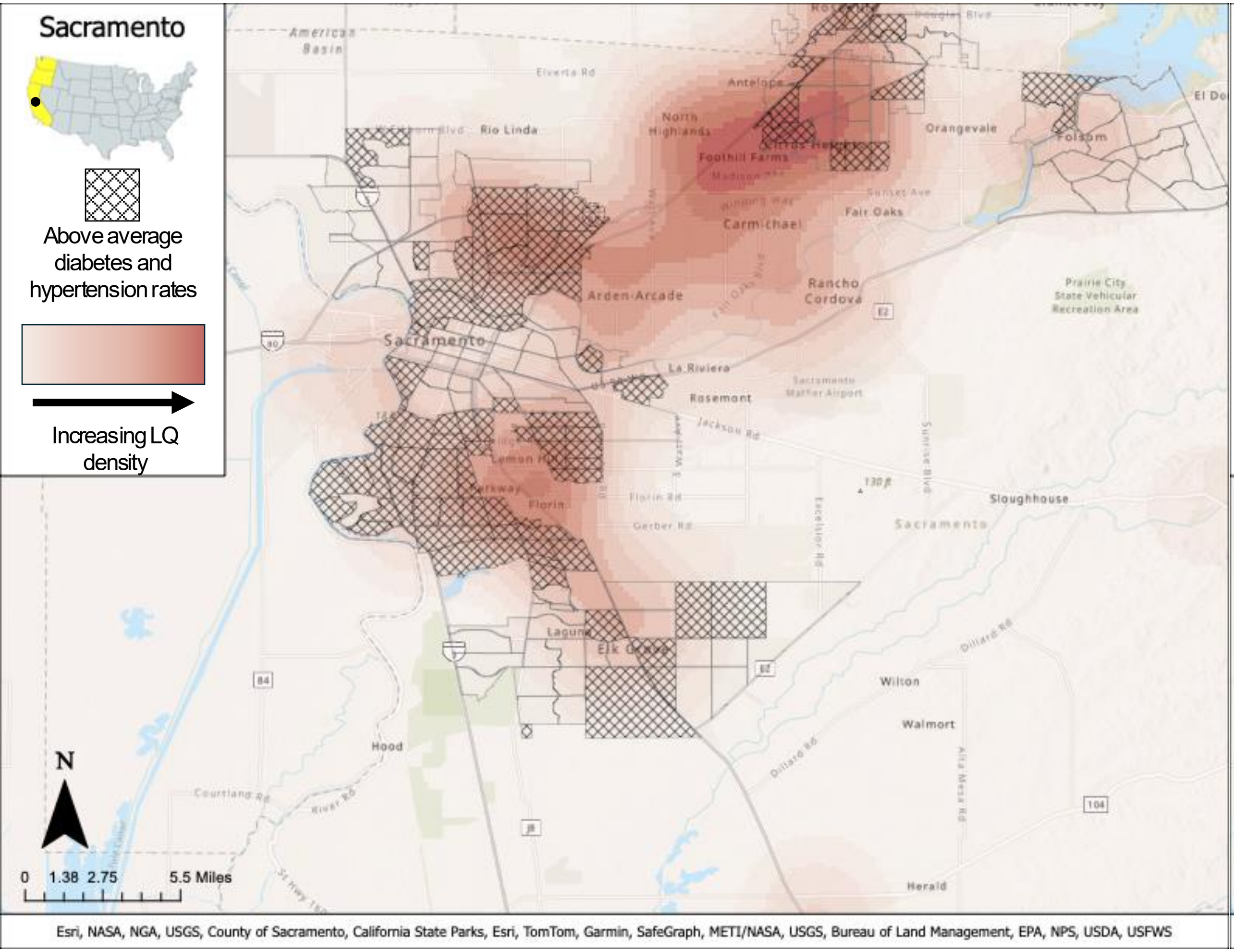

B)

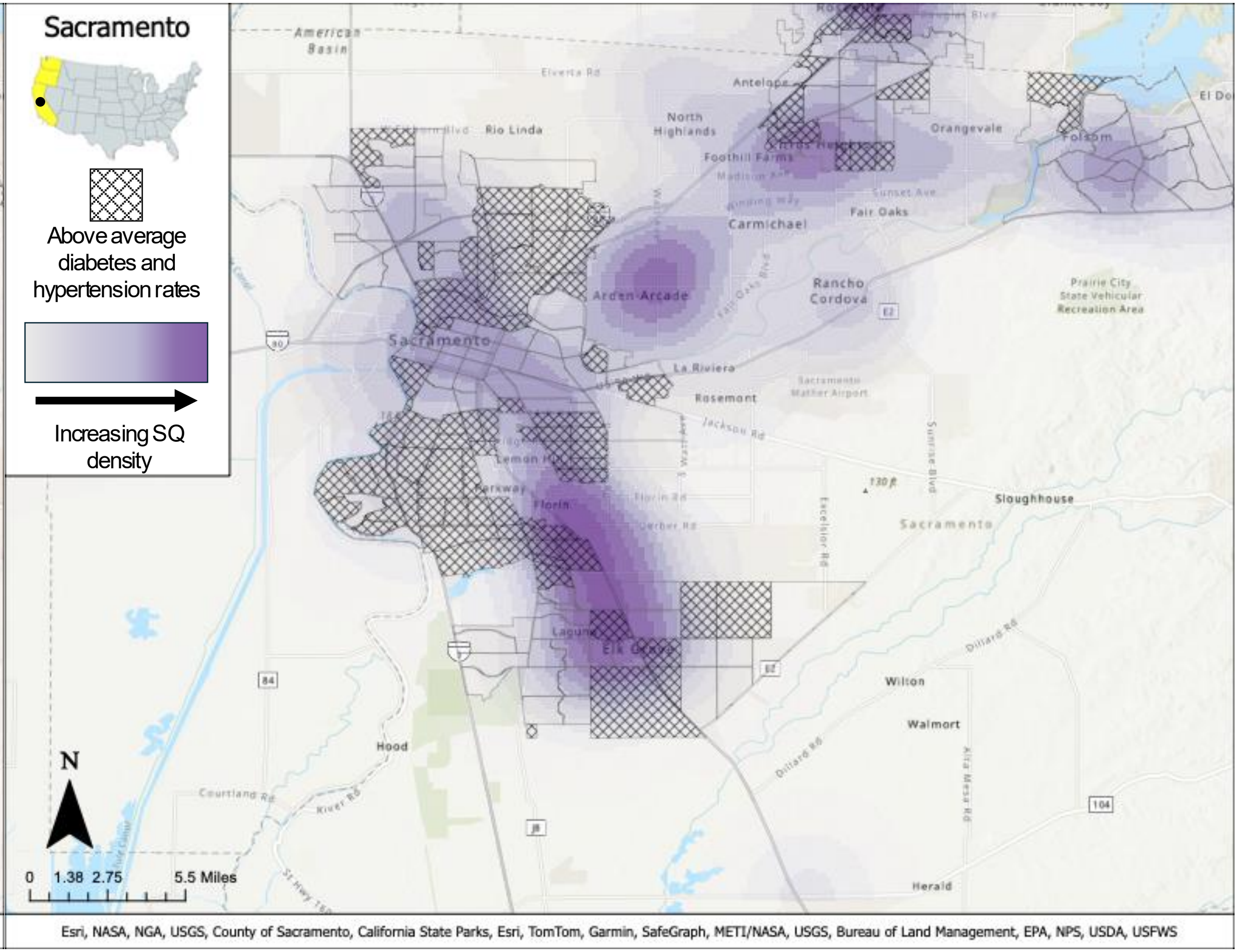

Side by side kernel analysis overlay with hatched tracts for the city of Sacramento, California. Hatched tracts represent above US average diabetes and hypertension rates. A) Red kernel shaded areas are LQ (dollar store) dense areas. B) Purple kernel shaded areas are SQ (traditional grocery store) dense areas. LQ = Low Quality, SQ = Standard Quality

Salt Lake City, Utah (Mountain Region)

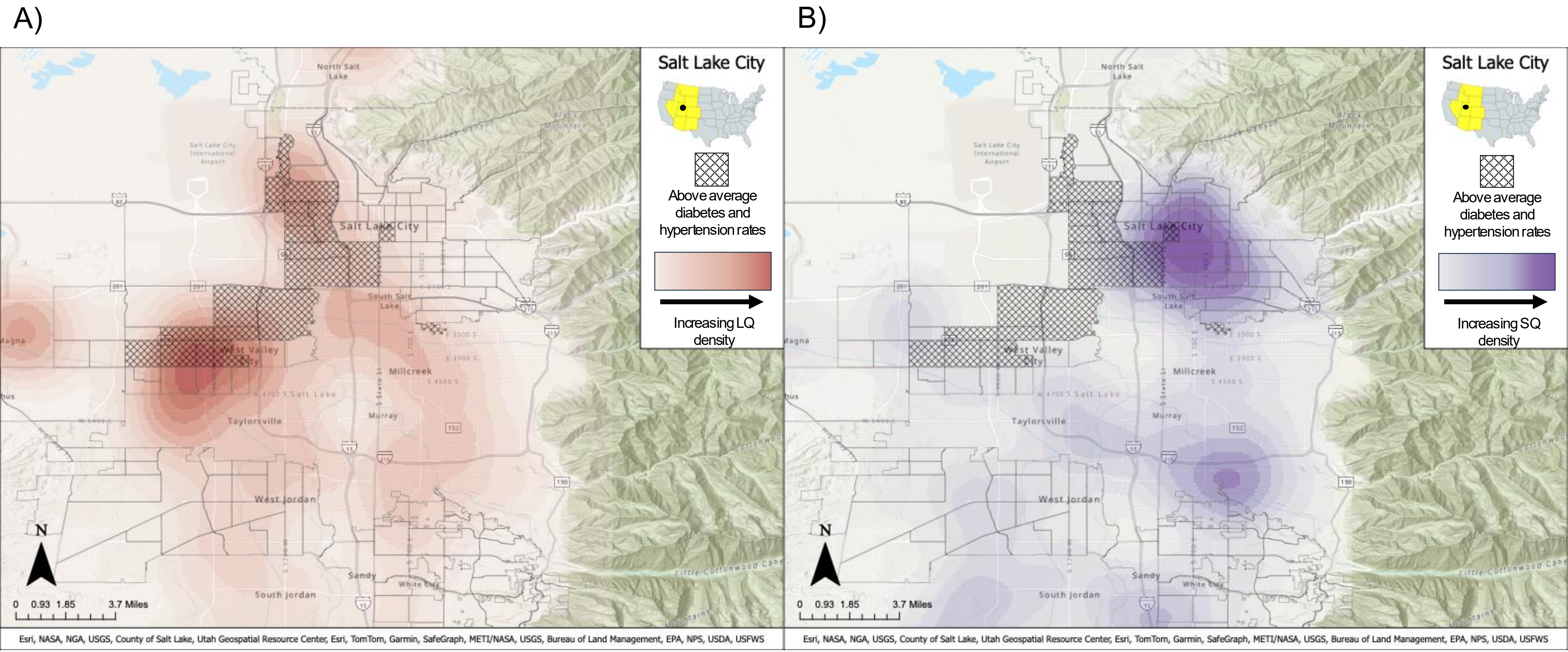

Side by side kernel analysis overlay with hatched tracts for the city of Salt Lake City, Utah. Hatched tracts represent above US average diabetes and hypertension rates. A) Red kernel shaded areas are LQ (dollar store) dense areas. B) Purple kernel shaded areas are SQ (traditional grocery store) dense areas. LQ = Low Quality, SQ = Standard Quality

San Antonio, Texas (South Region)

A)

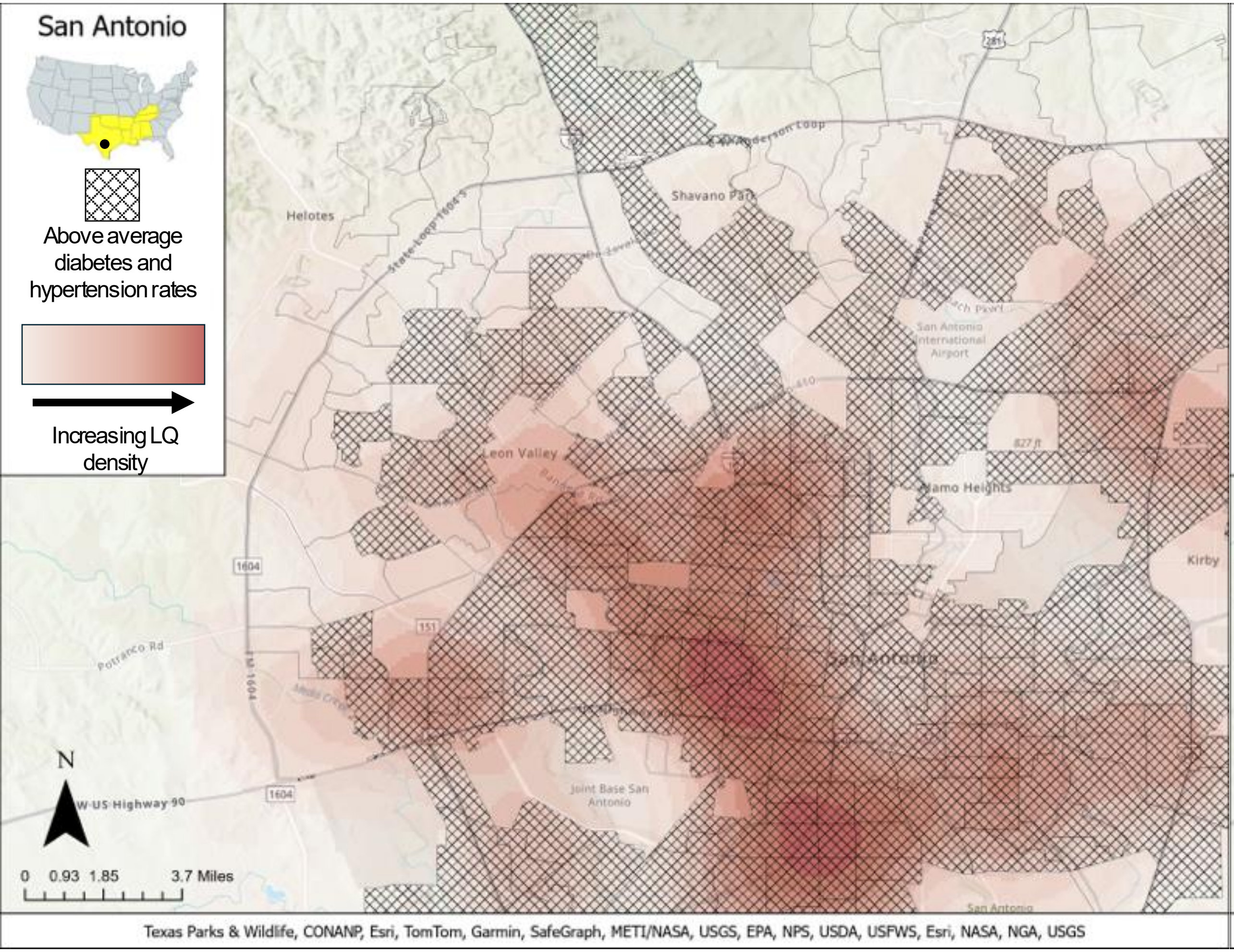

B)

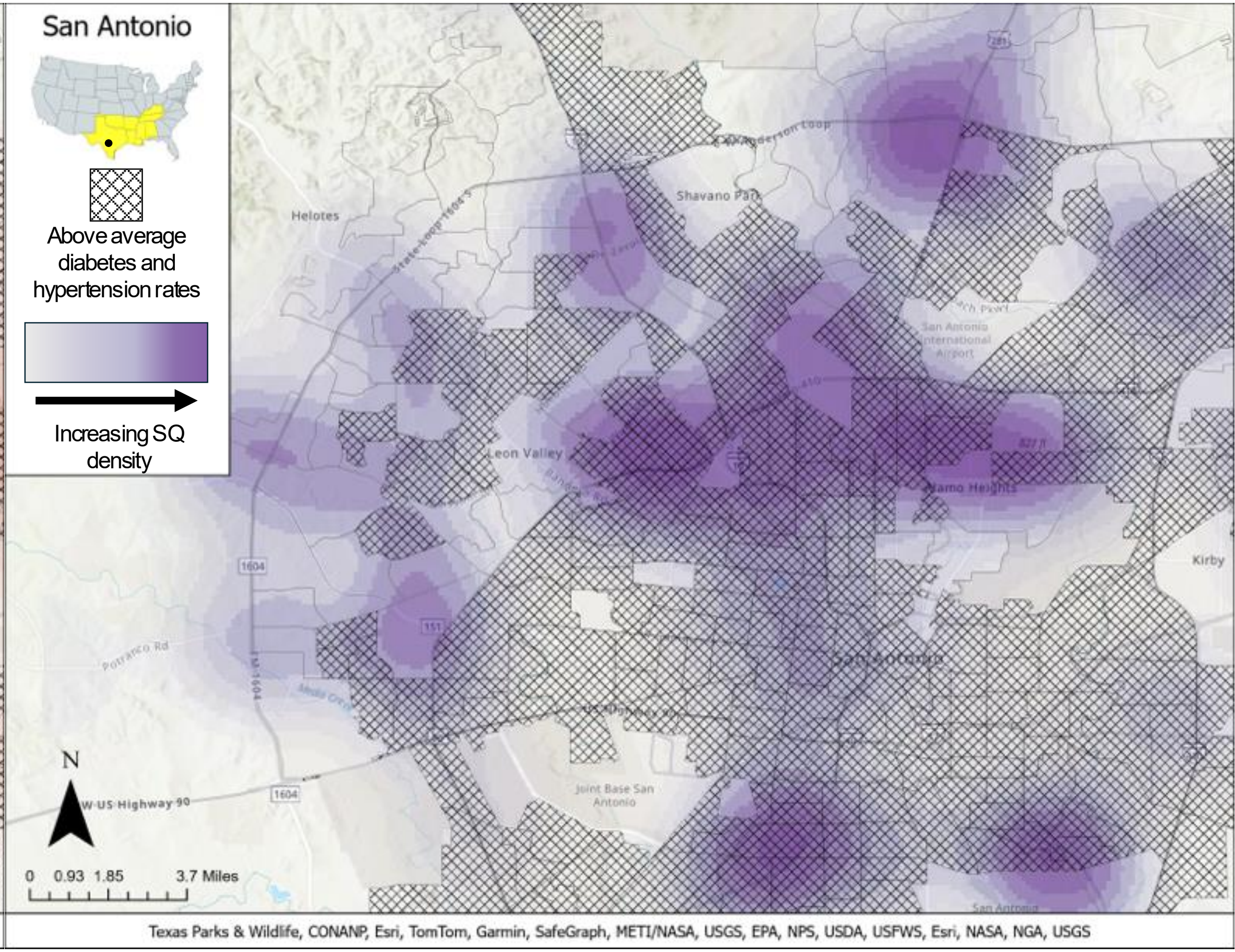

Side by side kernel analysis overlay with hatched tracts for the city of San Antonio, Texas. Hatched tracts represent above US average diabetes and hypertension rates. A) Red kernel shaded areas are LQ (dollar store) dense areas. B) Purple kernel shaded areas are SQ (traditional grocery store) den se areas. LQ = Low Quality, SQ = Standard Quality

San Diego, California (Pacific Region)

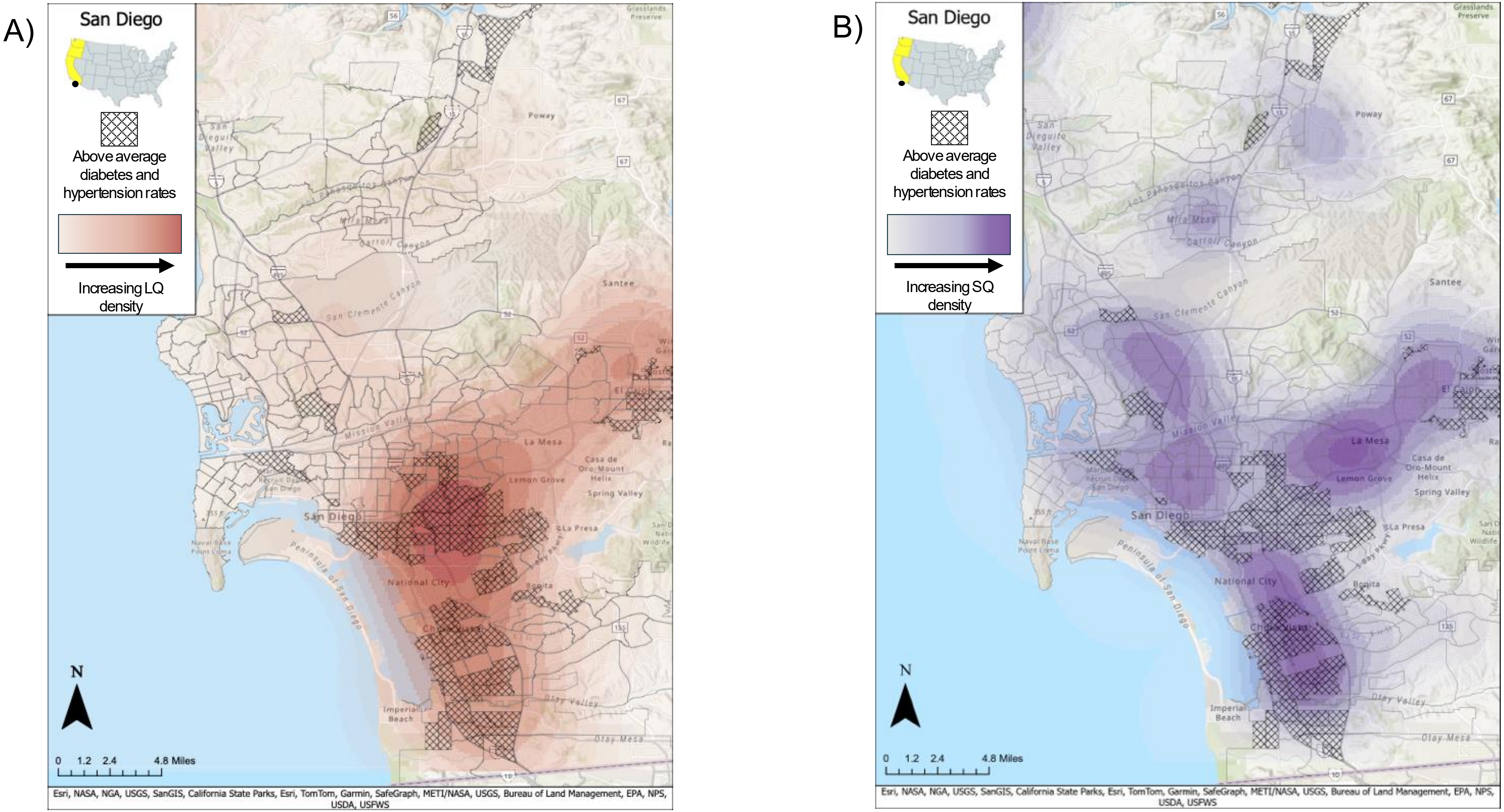

Side by side kernel analysis overlay with hatched tracts for the city of San Diego, California. Hatched tracts represent above US average diabetes and hypertension rates. A) Red kernel shaded areas are LQ (dollar store) dense areas. B) Purple kernel shaded areas are SQ (traditional grocery store) dense areas. LQ = Low Quality, SQ = Standard Quality

San Francisco Bay, California (Pacific Region)

A)

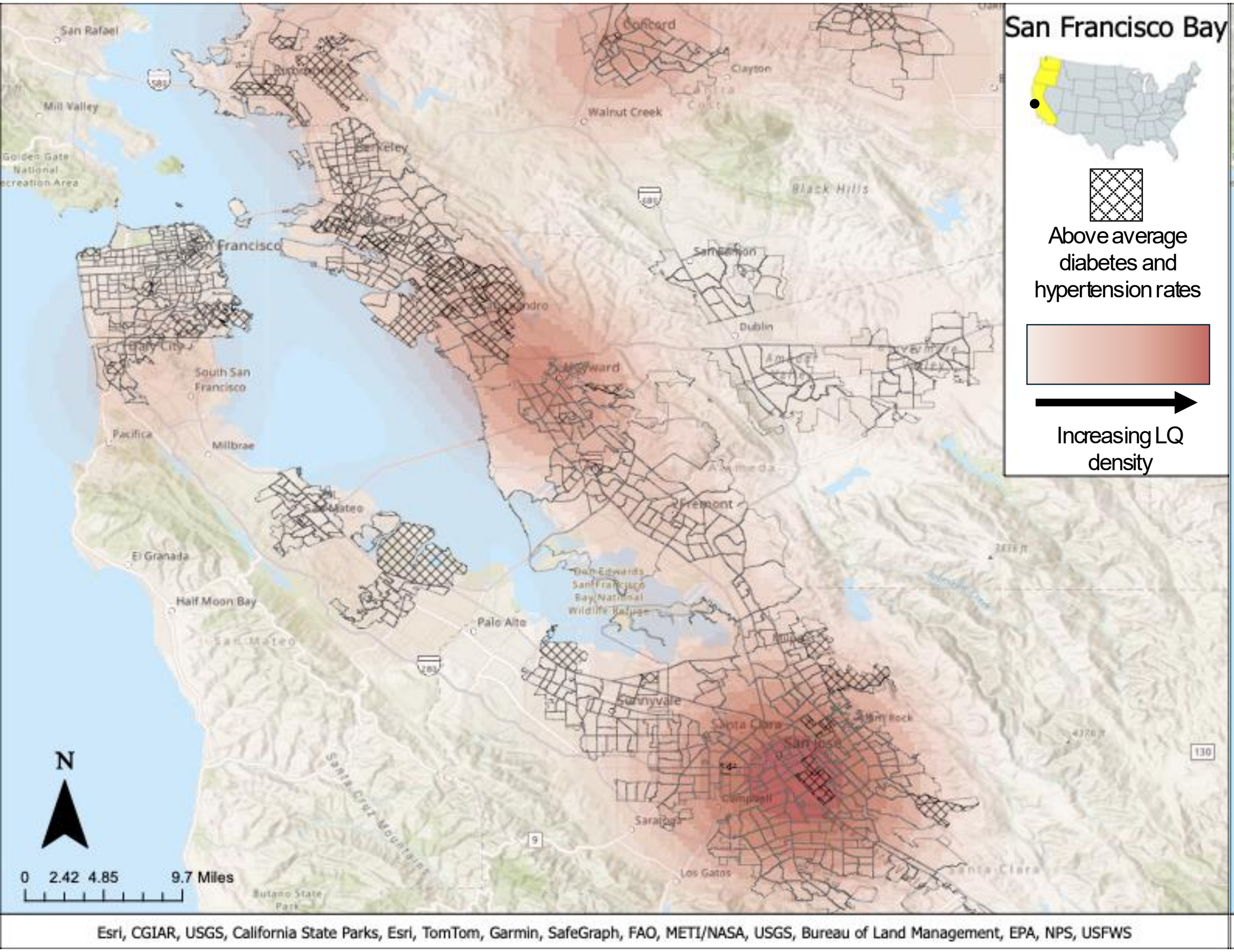

B)

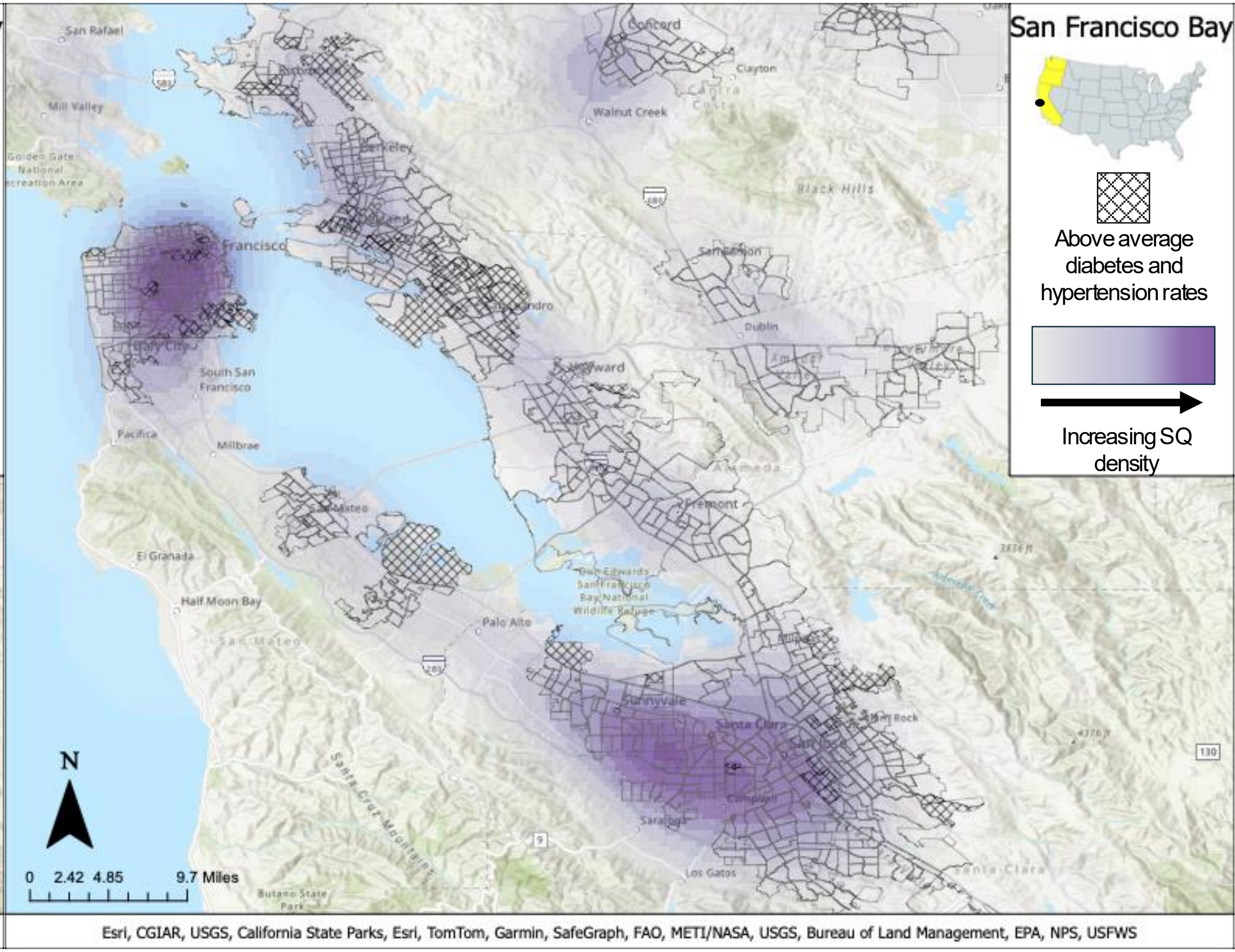

Side by side kernel analysis overlay with hatched tracts for the cities in San Francisco Bay, California (San Francisco, San Jose, Oakland). Hatched tracts represent above US average diabetes and hypertension rates. A) Red kernel shaded areas are LQ (dollar store) dense areas. B) Purple kernel shaded areas are SQ (traditional grocery store) dense areas. LQ = Low Quality, SQ = Standard Quality

Seattle, Washington (Pacific Region)

A) B)

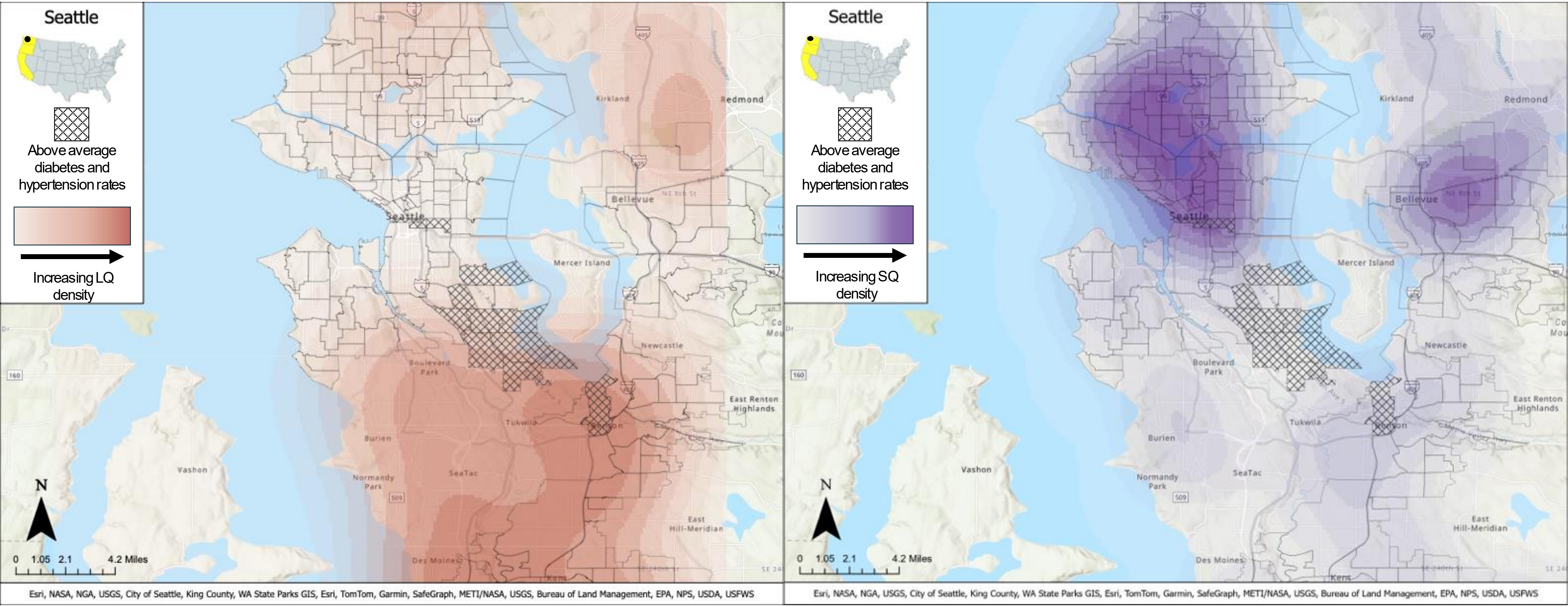

Side by side kernel analysis overlay with hatched tracts for the city of Seattle, Washington. Hatched tracts represent above US average diabetes and hypertension rates. A) Red kernel shaded areas are LQ (dollar store) dense areas. B) Purple kernel shaded areas are SQ (traditional grocery store) dense areas. LQ = Low Quality, SQ = Standard Quality

Staten Island, New York (Northeast Region)

A) B)

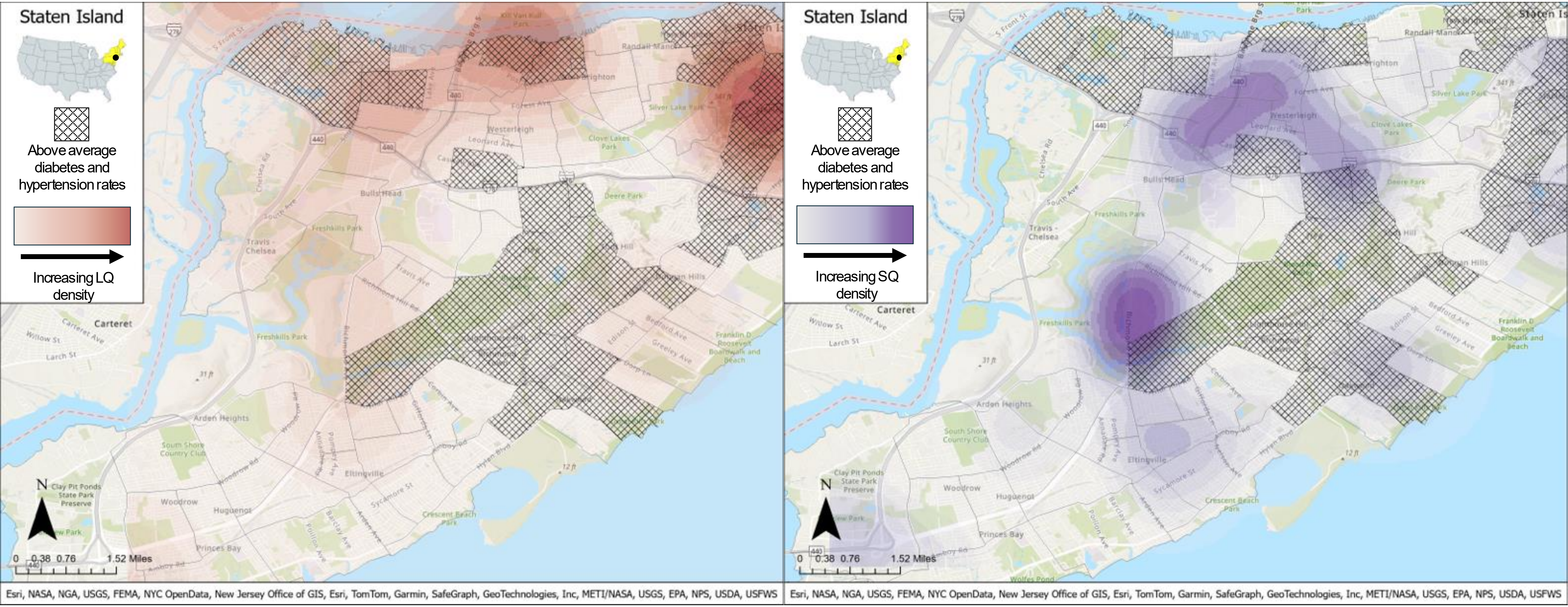

Side by side kernel analysis overlay with hatched tracts for New York City, New York (Staten Island). Hatched tracts represent above US average diabetes and hypertension rates. A) Red kernel shaded areas are LQ (dollar store) dense areas. B) Purple kernel shaded areas are SQ (traditional grocery store) dense areas. LQ = Low Quality, SQ = Standard Quality

St Louis, Missouri (Midwest Region)

A) B)

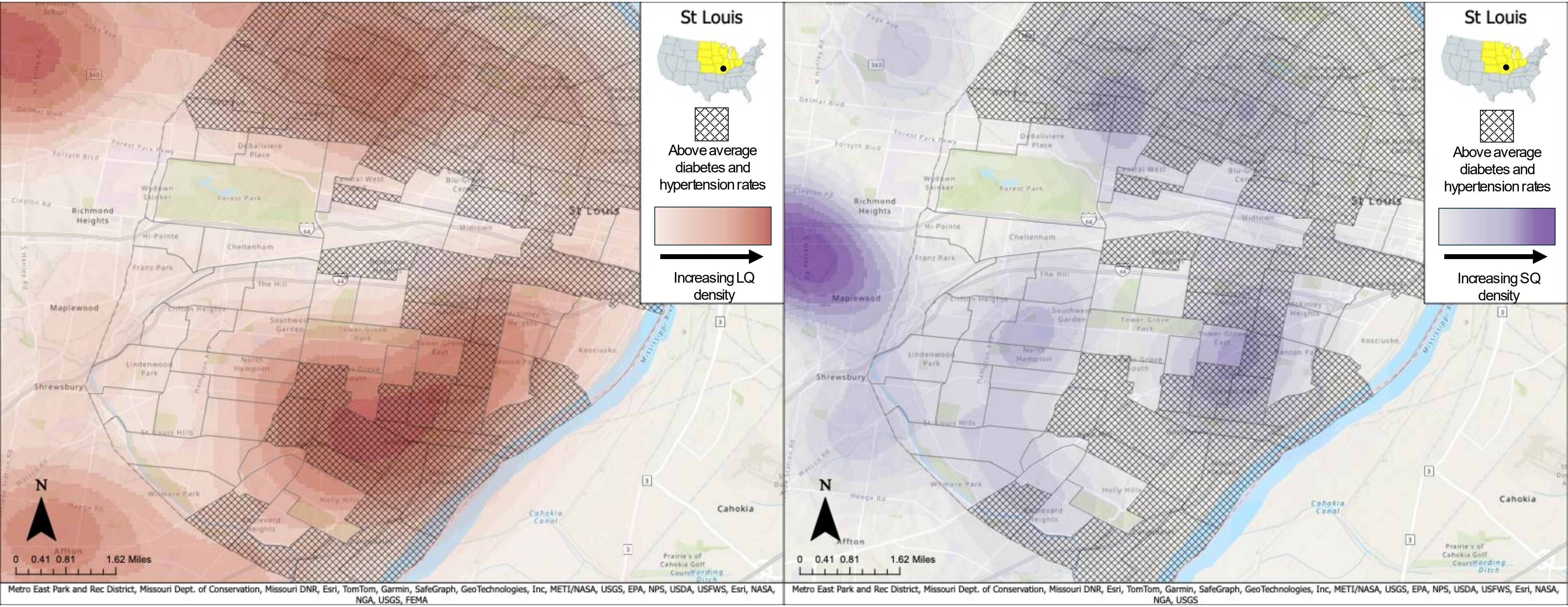

Side by side kernel analysis overlay with hatched tracts the city of St Louis, Missouri. Hatched tracts represent above US average diabetes and hypertension rates. A) Red kernel shaded areas are LQ (dollar store) dense areas. B) Purple kernel shaded areas are SQ (traditional grocery store) dense areas. LQ = Low Quality, SQ = Standard Quality

Tampa, Florida (South Atlantic Region)

A) B)

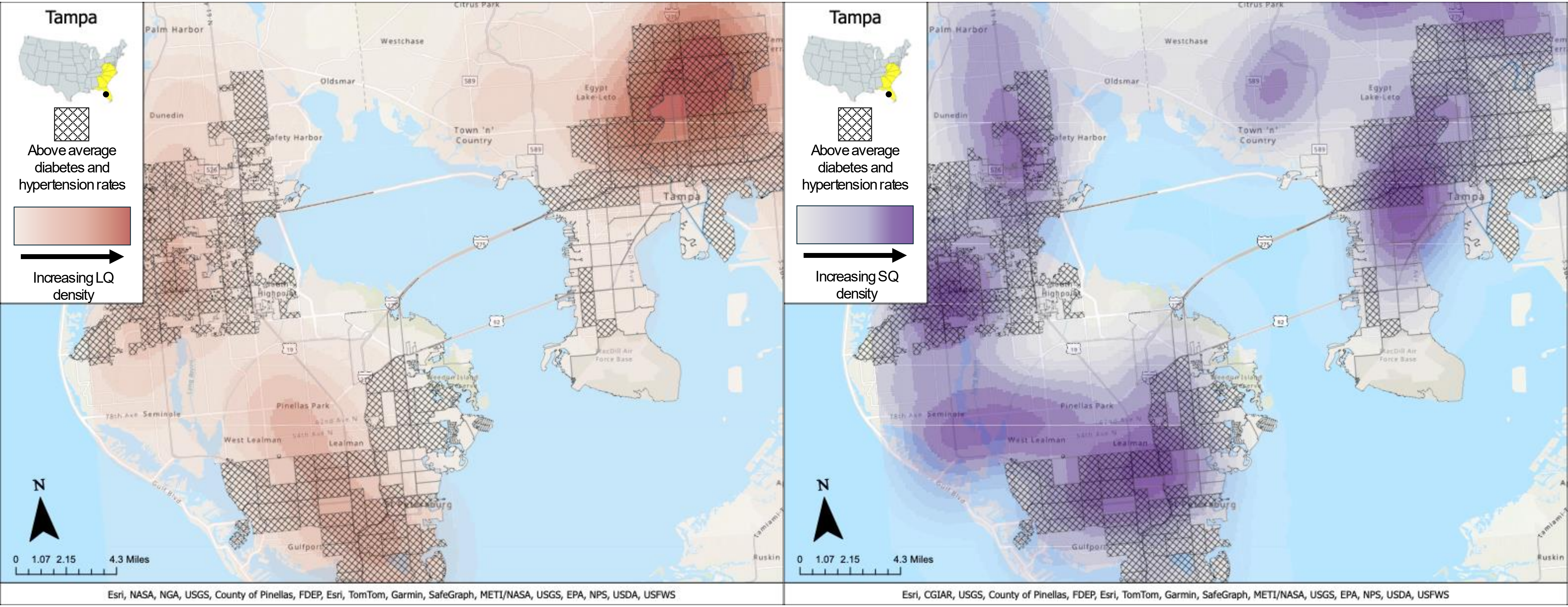

Side by side kernel analysis overlay with hatched tracts for the metro of Tampa, Florida. Hatched tracts represent above US average diabetes and hypertension rates. A) Red kernel shaded areas are LQ (dollar store) dense areas. B) Purple kernel shaded areas are SQ (traditional grocery store) dense areas. LQ = Low Quality, SQ = Standard Quality

Tucson, Arizona (Mountain Region)

A)

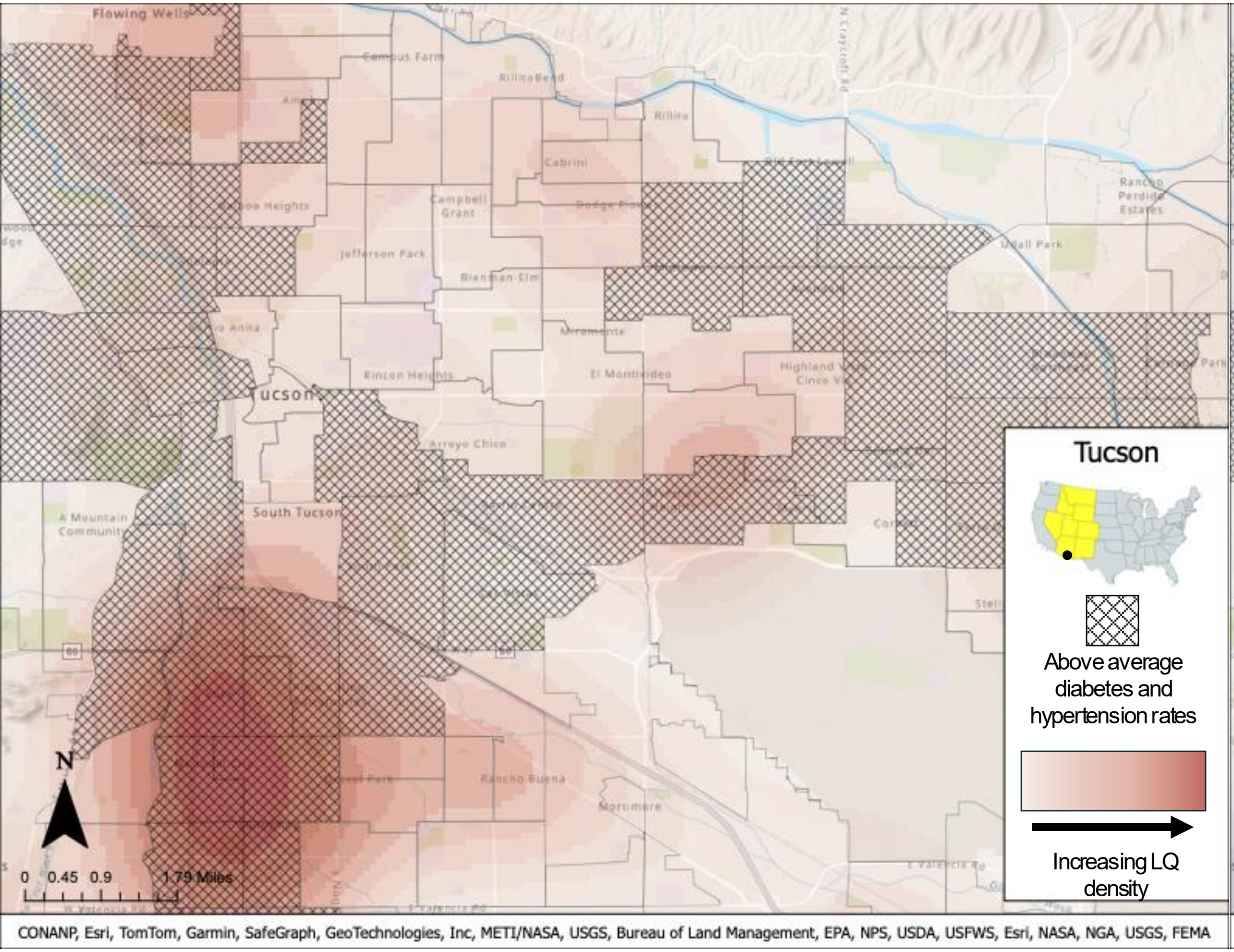

B)

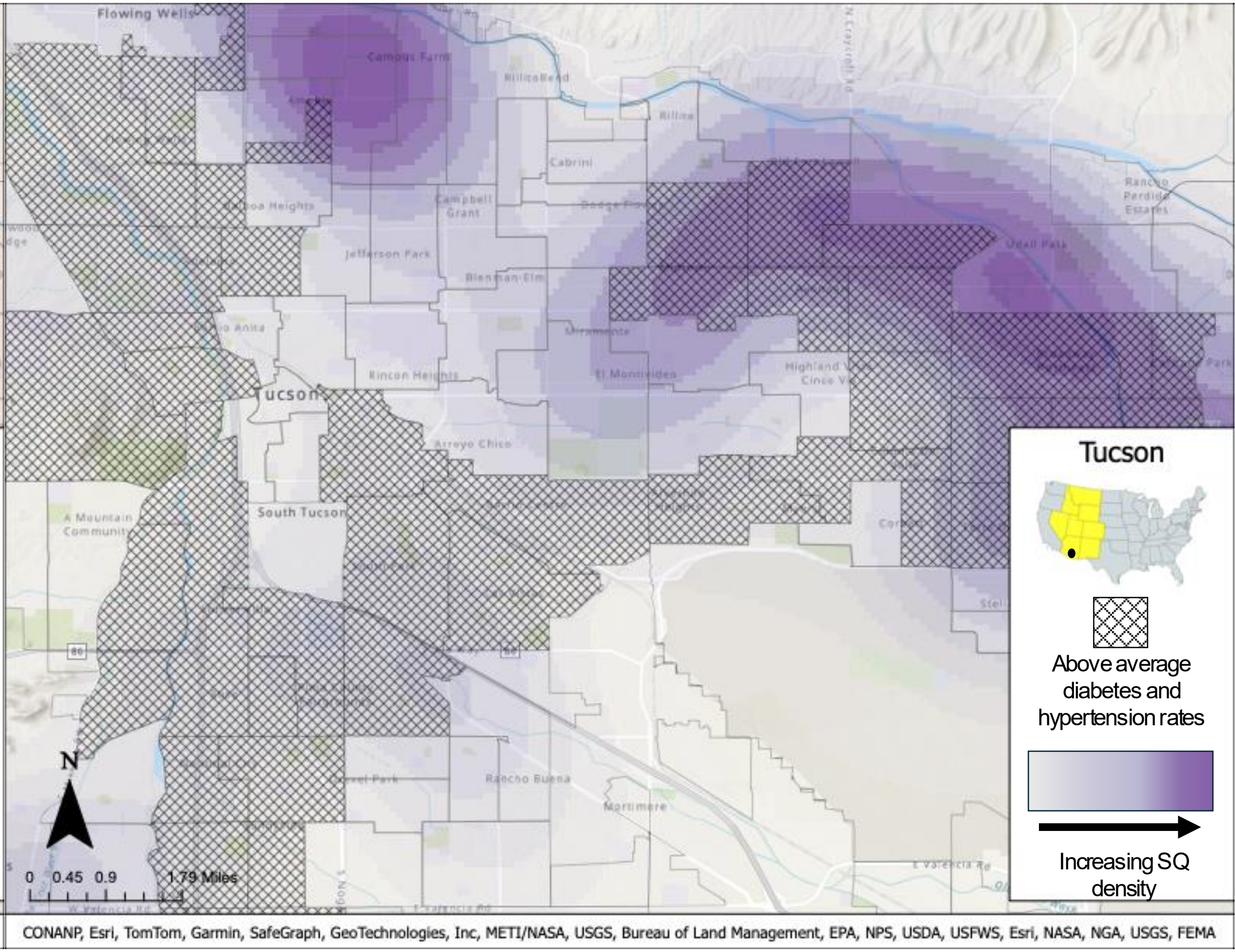

Side by side kernel analysis overlay with hatched tracts for the city of Tucson, Arizona. Hatched tracts represent above US average diabetes and hypertension rates. A) Red kernel shaded areas are LQ (dollar store) dense areas. B) Purple kernel shaded areas are SQ (traditional grocery store) dense areas. LQ = Low Quality, SQ = Standard Quality

Virginia Beach, Virginia (South Atlantic Region)

A) B)

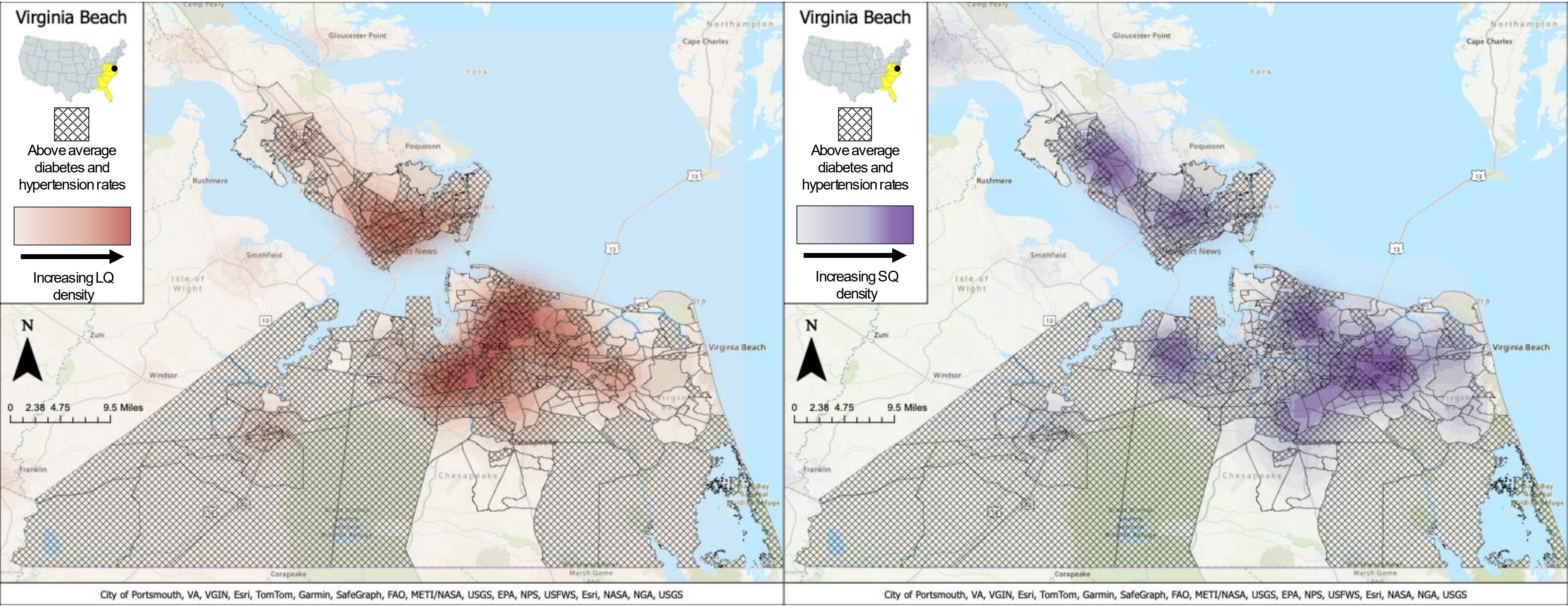

Side by side kernel analysis overlay with hatched tracts for the metro of Virginia Beach, Virginia. Hatched tracts represent above US average diabetes and hypertension rates. A) Red kernel shaded areas are LQ (dollar store) dense areas. B) Purple kernel shaded areas are SQ (traditional grocery store) dense areas. LQ = Low Quality, SQ = Standard Quality

Washington DC (Northeast Region)

A)

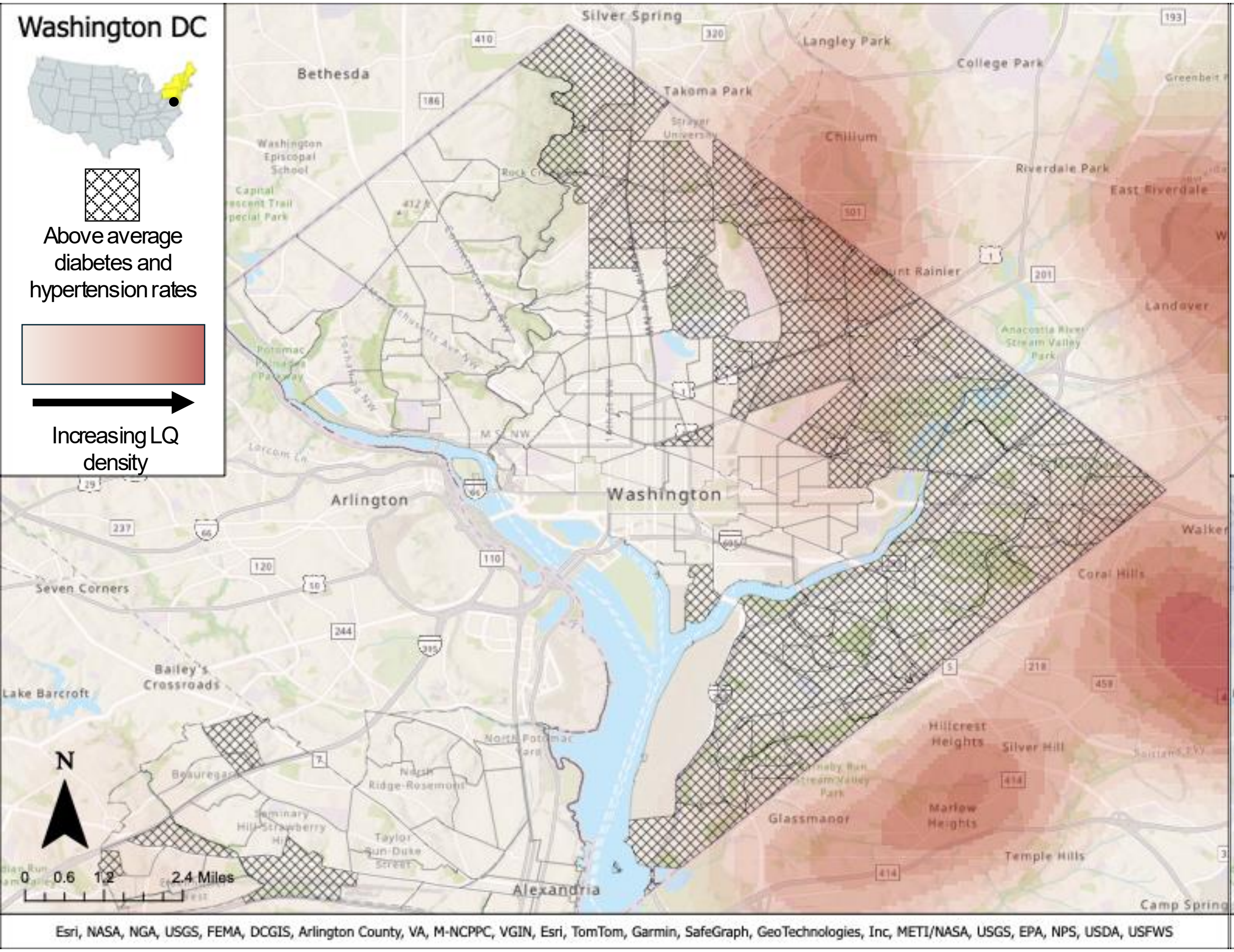

B)

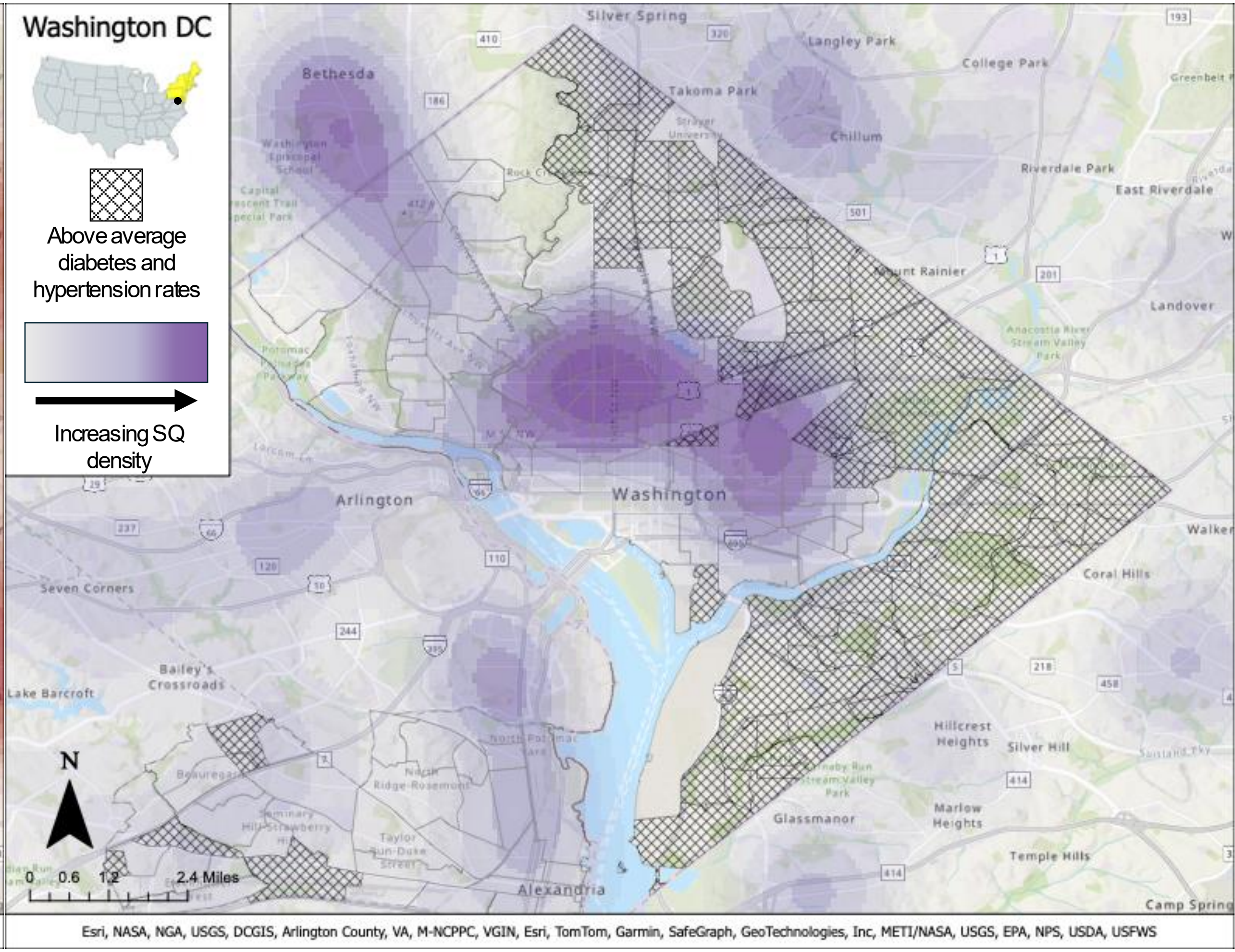

Side by side kernel analysis overlay with hatched tracts for Washington DC. Hatched tracts represent above US average diabetes and hypertension rates. A) Red kernel shaded areas are LQ (dollar store) dense areas. B) Purple kernel shaded areas are SQ (traditional grocery store) dense areas. LQ = Low Quality, SQ = Standard Quality
