# Supplementary material for: Beyond proximity: how food quality in dollar and grocery stores influences health
Source: BMC Public Health. 2026 Jun 18;26:3268. doi: 10.1186/s12889-026-28145-y (PMC13746574; doi:10.1186/s12889-026-28145-y)

Supplemental Figure 1

Visualization of regression results across contiguous United States and regions. Results are from all explanatory variables being standardized. High BP = High blood pressure; High Chol = High cholesterol, M Health = Mental Health. * = p-value <0.05, ** = p-value <0.01, *** = p-value <0.001, ns = p-value ≥ 0.05.

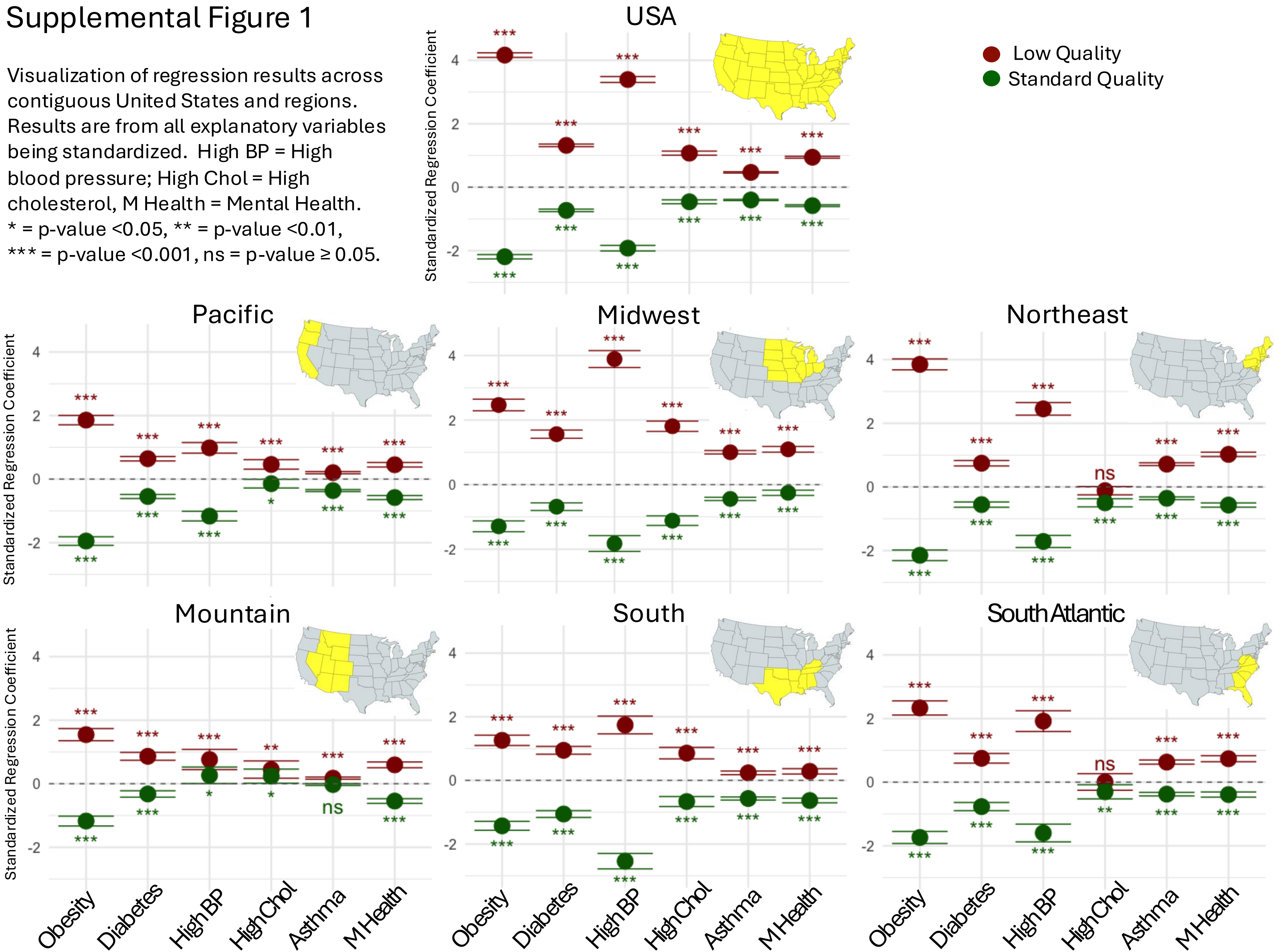

Supplement: Supplementary file 2 [file 12889_2026_28145_MOESM3_ESM.pdf]
