# Supplementary material for: Beyond proximity: how food quality in dollar and grocery stores influences health
Source: BMC Public Health. 2026 Jun 18;26:3268. doi: 10.1186/s12889-026-28145-y (PMC13746574; doi:10.1186/s12889-026-28145-y)
Supplement: Supplementary file 3 [file 12889_2026_28145_MOESM2_ESM.docx]

Supplemental Table 1

| **Grocery Store Datapoints Collected = 30,920 locations** | | | | | |
| --- | --- | --- | --- | --- | --- |
| 1st Oriental Supermarket | 888 Market | 99 Asian Market and Restaurant | 99 Ranch Market | Acme Fresh Market | Acme Market |
| AJ’s Fine Foods | Albertsons | Aldi | Andronico’s | Arteaga’s Food Center | Assi Plaza |
| Baker’s | Bashas’ | Bi-Lo | Big Saver Foods | Big Y | Big Y World Class Market |
| BJs Wholesale Club | BRAVO Supermarket | BreadBerry | Breaux Mart | Bristol Farms | Brookshire Brothers Foods |
| Brookshire’s | Broulim’s | Buehler’s | C-Market Foodtown | C-Mart | C-Town Fresh Market |
| CAM Asia Supermarket | CAM International Asian Market | Caraluzzi’s | Cardenas Market | Carrs | Cash & Carry |
| Cash Wise Foods Grocery Store | Central Market | Chavez Supermarket | Chief Supermarket | CityMarket | Clayton Straubs |
| Costco | County Market | Crest Foods | CTown Supermarkets | Cub Foods | D&W Fresh Market |
| Dagostino Supermarket | Dan’s Supermarket | Dave’s Market | Dave’s Supermarket | DG Market | Dierbergs |
| Dillons | Dominicks | Donelan’s Supermarket | Dorothy Lane Market | Earth Fare | Econofoods |
| El Ahorro Supermarket | El Rancho Supermercado | El Rey Market | El Rio Grande Latin Market | El Super | Erewhon Market |
| Evergreen | Extra Supermarket | Fairplay Foods | Fairway Market | Family Fare | Family Fare Supermarket |
| Fareway | Fei Long Market | Festival Foods | Fields Market | Fiesta Food Market | Fiesta Mart |
| Fine Fare Supermarket | Food 4 Less | Food City | Food Lion | Food Maxx International | Food Source |
| Food Town | Foodarama | Foodland Supermarkets | Foods Co | Foodtown | Fred Myer |
| Fresco y Mas | Fresh International Market | Fry’s | Fry’s Marketplace | Galleria Asian Market | Galleria Market |
| Geissler’s Supermarket | Gelsons | Gerbes | Gerrity’s Supermarket | Giant Eagle | Giant Food |
| Gleibermans | Global Food | Global Foods Market | Good Fortune Supermarket | Gourmet Glatt | Grand & Essex |
| Grand Asia Mart | Grand Asian Market | Grand Mart | Grant Mart International Foods | Great Wall Supermarket | Gristedes |
| Grocery Outlet | H Mart | H-E-B | H-mart | Haggen Food & Pharmacy | Han Nam Chain |
| Han Yang Market | Hang Yang Mart | Hannaford | Hannam | Hanyang Mart | Happy Foods |
| Harding’s | Harmons | Harps Food Stores | Harris Teeter | Harvest Foods | Harveys |
| Heinen’s Fine Foods | Hen House | Highland Park Market | Hitchcock’s Markets | Homeland Grocery Store | Hometown Foods |
| Hong Kong Food Market | Hong Kong Supermarket | Hugo’s | HyVee | IGA | India Bazaar |
| Ingles | Island Pacific Supermarket | J Mart | Jay C | Jerrys Foods | Jewel |
| Jewel/Osco | Kam Man Food | Karns Foods | Keys Foods | King Kullen | King Soopers |
| Klein’s Supermarket | Kosher Konnection | Kroger | Kuhns | La Bonita Supermarkets | La Michoacana Meat Market |
| La Perla Tapatia Supermarket | La Placita Supermarket | Lassens Natural Food and Vitamins | Lawrence Plaza Galleria Market & Mall | Lazy Acres | LiDL |
| Lotte Plaza | Lowe’s Market | Lowes Foods | Lunds & Byerlys | Magruders | Marc’s |
| Mariano’s | Market 32 | Market Basket | Market of Choice | Martins Supermarket | Marukai |
| Meijer | Met Foodmarkets | Metro Market | Mi Tienda | Mitsuwa | Morton Williams |
| Mosers | Nam Dae Mun | Natural Grocers | New India Bazar | New Seasons Market | New Way Market |
| Newday | Nijiya | Northgate Market | Nugget Markets | Numero Uno Market | Ocean Mart |
| Olivers Market | Pacific Ocean Market | Pacific Ocean Marketplace | Pacific Supermarket | Patel Brothers | Pavilions |
| Payless Supermarket | PCC Natural Markets | Pic Pac Supermarket | Pick’n Save | PigglyWiggly | Pioneer Supermarket |
| President Supermarket | Presidente Supermarket | Price Chopper | Price Cutter | PriceRite | Publix |
| QFC | R-Ranch Markets | Raley’s | Ralphs | Rancho Market | Randalls |
| Ranis World Foods | Reasor’s Foods | Red Apple Market | Redner’s | Remke | Ridley’s Family Markets |
| Riesbeck’s | Roche Bros | Rockland Kosher | Rosauers Supermarkets | Rouses | Ruler |
| Safeway | Sam’s Club | Sav A Lot | Save Mart | Save-A-Lot | Saver’s Cost Plus |
| Schnucks | Scolaris | Seabra Foods | Seafood City | Sedano’s | Sellers Brothers |
| Sendik’s Food Market | Sentry Foods | Seven Mile Market | Shamrock Foods | Sharp Shopper | Shaws |
| Shoppers Food | Shoprite | Shun Fat Supermarket | Sky Foods | Smart & Final | Smith’s |
| Smith’s Marketplace | Sparkle Market | Sprouts | Star Market | Stater Brothers Market | Stew Leonards |
| Stop & Shop | Strack & Van Til | Straubs | Sullivan’s Foods | Super A Foods | Super Foodtown |
| Super Fresh | Super G Mart | Super H Mart | Super King | Superior Grocers | Superior Super Warehouse |
| Supermercado El Rancho | Supermercados Teloloapan | SuperOne Foods | Supersaver Foods | Target | The Food Emporium |
| The Fresh Grocer | The Fresh Market | The Market Place | Times Supermarket | Trader Joe’s | Tresierras Supermarket |
| Twin City Supermarket | United Grocery Outlet | United Supermarkets | US Foods Chef Store | Uwajimaya | Vallarta |
| Viva Markets | Vons | Walmart | Walmart Neighborhood Market | Wayfield Foods | Wegmans |
| Weis | Weis Market | Wesley Kosher | Westborn Market | Western Beef | Western Kosher |
| Whole Foods | WinCo | Winn Dixie | zTao Marketplace |  |  |

| **Dollar Stores Datapoints Collected = 34,021 locations** | | | |
| --- | --- | --- | --- |
| 99 Cents Only | Dollar General | Dollar Tree | Family Dollar |

Supplemental Table 2

| Regression Results  Non-standardized Variables | | Obesity | | | Diabetes | | | High BP | | |
| --- | --- | --- | --- | --- | --- | --- | --- | --- | --- | --- |
|  |  | Coef | SE | p-value | Coef | SE | p-value | Coef | SE | p-value |
| USA | Intercept | 29.45 | 0.03 | *** | 8.06 | 0.07 | *** | 30.07 | 0.05 | *** |
|  | LQ Grocery Stores | **0.34** | **<0.01** | ******* | **0.11** | **<0.01** | ******* | **0.28** | **<0.01** | ******* |
|  | SQ Grocery Stores | **-0.17** | **<0.01** | ******* | **-0.06** | **<0.01** | ******* | **-0.15** | **<0.01** | ******* |
|  | SVI Overall | **11.78** | **0.12** | ******* | **8.06** | **0.07** | ******* | **8.95** | **0.16** | ******* |
| Midwest | Intercept | 35.25 | 0.08 | *** | 12.12 | 0.06 | *** | 33.67 | 0.12 | *** |
|  | LQ Grocery Stores | **0.19** | **0.01** | ******* | **0.12** | **0.01** | ******* | **0.30** | **0.01** | ******* |
|  | SQ Grocery Stores | **-0.13** | **0.01** | ******* | **-0.07** | **0.01** | ******* | **-0.19** | **0.01** | ******* |
|  | SVI Overall | **17.70** | **0.30** | ******* | **9.94** | **0.21** | ******* | **14.78** | **0.43** | ******* |
| Mountain | Intercept | 27.04 | 0.07 | *** | 8.54 | 0.05 | *** | 26.42 | 0.12 | *** |
|  | LQ Grocery Stores | **0.17** | **0.01** | ******* | **0.10** | **0.01** | ******* | **0.09** | **0.02** | ******* |
|  | SQ Grocery Stores | **-0.10** | **0.01** | ******* | **-0.03** | **<0.01** | ******* | **0.02** | **0.01** | ***** |
|  | SVI Overall | **9.78** | **0.29** | ******* | **4.04** | **0.19** | ******* | **3.78** | **0.48** | ******* |
| Northeast | Intercept | 27.81 | 0.08 | *** | 11.18 | 0.04 | *** | 29.94 | 0.10 | *** |
|  | LQ Grocery Stores | **0.43** | **0.01** | ******* | **0.08** | **<0.01** | ******* | **0.28** | **0.01** | ******* |
|  | SQ Grocery Stores | **-0.14** | **0.01** | ******* | **-0.04** | **<0.01** | ******* | **-0.12** | **0.01** | ******* |
|  | SVI Overall | **15.61** | **0.33** | ******* | **10.48** | **0.16** | ******* | **14.69** | **0.37** | ******* |
| Pacific | Intercept | 24.43 | 0.05 | *** | 9.40 | 0.03 | *** | 26.25 | 0.06 | *** |
|  | LQ Grocery Stores | **0.33** | **0.01** | ******* | **0.11** | **0.01** | ******* | **0.18** | **0.02** | ******* |
|  | SQ Grocery Stores | **-0.14** | **0.01** | ******* | **-0.04** | **<0.01** | ******* | **-0.08** | **0.01** | ******* |
|  | SVI Overall | **10.58** | **0.21** | ******* | **5.87** | **0.10** | ******* | **4.52** | **0.23** | ******* |
| South | Intercept | 34.10 | 0.07 | *** | 12.08 | 0.05 | *** | 33.54 | 0.12 | *** |
|  | LQ Grocery Stores | **0.09** | **0.01** | ******* | **0.07** | **<0.01** | ******* | **0.13** | **0.01** | ******* |
|  | SQ Grocery Stores | **-0.10** | **0.01** | ******* | **-0.08** | **<0.01** | ******* | **-0.18** | **0.01** | ******* |
|  | SVI Overall | **15.69** | **0.26** | ******* | **9.13** | **0.20** | ******* | **10.33** | **0.45** | ******* |
| South Atlantic | Intercept | 31.08 | 0.09 | *** | 11.01 | 0.06 | *** | 32.58 | 0.13 | *** |
|  | LQ Grocery Stores | **0.22** | **0.01** | ******* | **0.07** | **0.01** | ******* | **0.18** | **0.02** | ******* |
|  | SQ Grocery Stores | **-0.18** | **0.01** | ******* | **-0.08** | **0.01** | ******* | **-0.17** | **0.01** | ******* |
|  | SVI Overall | **12.48** | **0.35** | ******* | **8.72** | **0.24** | ******* | **12.27** | **0.51** | ******* |
|  |  |  | | |  | | |  | | |
|  |  | High Chol | | | Asthma | | | M Health | | |
|  |  | Coef | SE | p-value | Coef | SE | p-value | Coef | SE | p-value |
| USA | Intercept | 31.11 | 0.03 | *** | 9.63 | 0.01 | *** | 13.61 | 0.01 | *** |
|  | LQ Grocery Stores | **0.09** | **<0.01** | ******* | **0.04** | **<0.01** | ******* | **0.08** | **<0.01** | ******* |
|  | SQ Grocery Stores | **-0.04** | **<0.01** | ******* | **-0.03** | **<0.01** | ******* | **-0.04** | **<0.01** | ******* |
|  | SVI Overall | **2.44** | **0.11** | ******* | **2.52** | **0.03** | ******* | **7.81** | **0.05** | ******* |
| Midwest | Intercept | 31.21 | 0.07 | *** | 10.45 | 0.03 | *** | 14.70 | 0.04 | *** |
|  | LQ Grocery Stores | **0.14** | **0.01** | ******* | **0.08** | **<0.01** | ******* | **0.08** | **<0.01** | ******* |
|  | SQ Grocery Stores | **-0.12** | **0.01** | ******* | **-0.05** | **<0.01** | ******* | **-0.03** | **<0.01** | ******* |
|  | SVI Overall | **2.61** | **0.26** | ******* | **3.31** | **0.09** | ******* | **8.68** | **0.15** | ******* |
| Mountain | Intercept | 30.15 | 0.10 | *** | 9.96 | 0.01 | *** | 13.14 | 0.03 | *** |
|  | LQ Grocery Stores | **0.05** | **0.02** | ****** | **0.02** | **<0.01** | ******* | **0.07** | **0.01** | ******* |
|  | SQ Grocery Stores | **0.02** | **0.01** | ***** | < -0.01 | <0.01 | 0.071 | **-0.05** | **<0.01** | ******* |
|  | SVI Overall | 0.17 | 0.41 | 0.678 | **1.67** | **0.05** | ******* | **7.03** | **0.14** | ******* |
| Northeast | Intercept | 30.78 | 0.06 | *** | 10.24 | 0.02 | *** | 13.84 | 0.03 | *** |
|  | LQ Grocery Stores | -0.01 | 0.01 | 0.088 | **0.08** | **<0.01** | ******* | **0.12** | **<0.01** | ******* |
|  | SQ Grocery Stores | **-0.03** | **<0.01** | ******* | **-0.02** | **<0.01** | ******* | **-0.04** | **<0.01** | ******* |
|  | SVI Overall | **5.93** | **0.25** | ******* | **3.03** | **0.08** | ******* | **8.38** | **0.13** | ******* |
| Pacific | Intercept | 30.13 | 0.05 | *** | 8.92 | 0.01 | *** | 12.50 | 0.03 | *** |
|  | LQ Grocery Stores | **0.08** | **0.01** | ******* | **0.04** | **<0.01** | ******* | **0.08** | **0.01** | ******* |
|  | SQ Grocery Stores | **-0.01** | **<0.01** | ***** | **-0.03** | **<0.01** | ******* | **-0.04** | **<0.01** | ******* |
|  | SVI Overall | **1.40** | **0.21** | ******* | **1.74** | **0.05** | ******* | **7.60** | **0.10** | ******* |
| South | Intercept | 32.98 | 0.08 | *** | 9.25 | 0.02 | *** | 14.28 | 0.04 | *** |
|  | LQ Grocery Stores | **0.06** | **0.01** | ******* | **0.02** | **<0.01** | ******* | **0.02** | **<0.01** | ******* |
|  | SQ Grocery Stores | **-0.05** | **0.01** | ******* | **-0.04** | **<0.01** | ******* | **-0.04** | **<0.01** | ******* |
|  | SVI Overall | **2.99** | **0.29** | ******* | **2.22** | **0.09** | ******* | **8.58** | **0.14** | ******* |
| South Atlantic | Intercept | 32.07 | 0.11 | *** | 9.23 | 0.03 | *** | 13.64 | 0.04 | *** |
|  | LQ Grocery Stores | <0.01 | 0.01 | 0.960 | **0.06** | **<0.01** | ******* | **0.07** | **<0.01** | ******* |
|  | SQ Grocery Stores | **-0.03** | **0.01** | ****** | **-0.04** | **<0.01** | ******* | **-0.04** | **<0.01** | ******* |
|  | SVI Overall | **3.62** | **0.41** | ******* | **1.95** | **0.10** | ******* | **7.50** | **0.15** | ******* |

Notes: BP = blood pressure; Chol = cholesterol; M Health = mental health; Coef = coefficient, SE = standard error; LQ = low-quality; SQ = standard-quality; SVI = Social Vulnerability Index; * = p-value <0.05, ** = p-value <0.01, *** = p-value <0.001

Supplemental Table 3

| Obesity Rate Correlation | 2016-2017 |  | High Chol Rate Correlation | 2016-2017 |
| --- | --- | --- | --- | --- |
| 2013-2014 | 0.96 |  | 2013-2014 | 0.92 |
| 2019-2020 | 0.95 |  | 2019-2020 | 0.89 |
| 2021-2022 | 0.93 |  | 2021-2022 | 0.81 |
|  |  |  |  |  |
| Diabetes Rate Correlation | 2016-2017 |  | Asthma Rate Correlation | 2016-2017 |
| 2013-2014 | 0.97 |  | 2013-2014 | 0.92 |
| 2019-2020 | 0.97 |  | 2019-2020 | 0.94 |
| 2021-2022 | 0.90 |  | 2021-2022 | 0.89 |
|  |  |  |  |  |
| High BP Rate Correlation | 2016-2017 |  | M Health Rate Correlation | 2016-2017 |
| 2013-2014 | 0.96 |  | 2013-2014 | 0.93 |
| 2019-2020 | 0.96 |  | 2019-2020 | 0.92 |
| 2021-2022 | 0.91 |  | 2021-2022 | 0.88 |
| Notes: High BP = High blood pressure, High Chol = High cholesterol, M Health = Mental health. | | | | |
